# α-Carbonyl Rh-Carbenoid Initiated Cascade Assembly of Diazobarbiturates with Alkylidene Pyrazolones for Synthesis of Spirofuropyrimidines

**DOI:** 10.3390/molecules29133178

**Published:** 2024-07-03

**Authors:** Yue Zhang, Yu-Hang Mi, Kuo Wang, Hong-Wu Zhao

**Affiliations:** College of Life Science and Bio-Engineering, Beijing University of Technology, No. 100 Pingleyuan, Chaoyang District, Beijing 100124, China; zhangyueyue@emails.bjut.edu.cn (Y.Z.); migenggeng@emails.bjut.edu.cn (Y.-H.M.); wk1632748255@emails.bjut.edu.cn (K.W.)

**Keywords:** cascade assembly, metal carbenoid, diazobarbiturate, alkylidene pyrazolone, spirofuropyrimidine

## Abstract

Catalyzed by Rh_2_(esp)_2_ (10 mol%) and (±)-BINAP (20 mol%) in DCE at 80 °C, the cascade assembly between diazobarbiturates and alkylidene pyrazolones proceeded readily and produced spiro-furopyrimidines in 38–96% chemical yields. The chemical structure of the prepared spirofuro-pyrimidines was firmly confirmed by X-ray diffraction analysis.

## 1. Introduction

Furopyrimidines constitute a family of privileged drug scaffolds and their analogs have a wide range of bioactivities such as antifungal, antitumor, antifolate, antimicrobial, antivirus, and antihuman cytomegalovirus properties [1,2,3]. Since the significant bioactivities with furopyrimidine skeletons, numerous efficient and facile synthetic protocols have been accomplished to approach highly functionalized furopyrimidine analogs [4,5,6,7,8,9,10,11,12]. Moreover, in this context, mechanistically diverse synthetic methodologies have been invented to deliver structurally unique and potentially bioactive spirofuropyrimidines [13,14,15,16,17,18]. Presently, the metal carbenoid-initiated cascade assembly of multiple molecules has barely been applied in the preparation of spirofuropyrimidines [19]. So, the development of metal carbenoid-based three-molecule cascades is highly desirable for preparing spirofuropyrimidines efficiently and facilely.

**Scheme 1:**
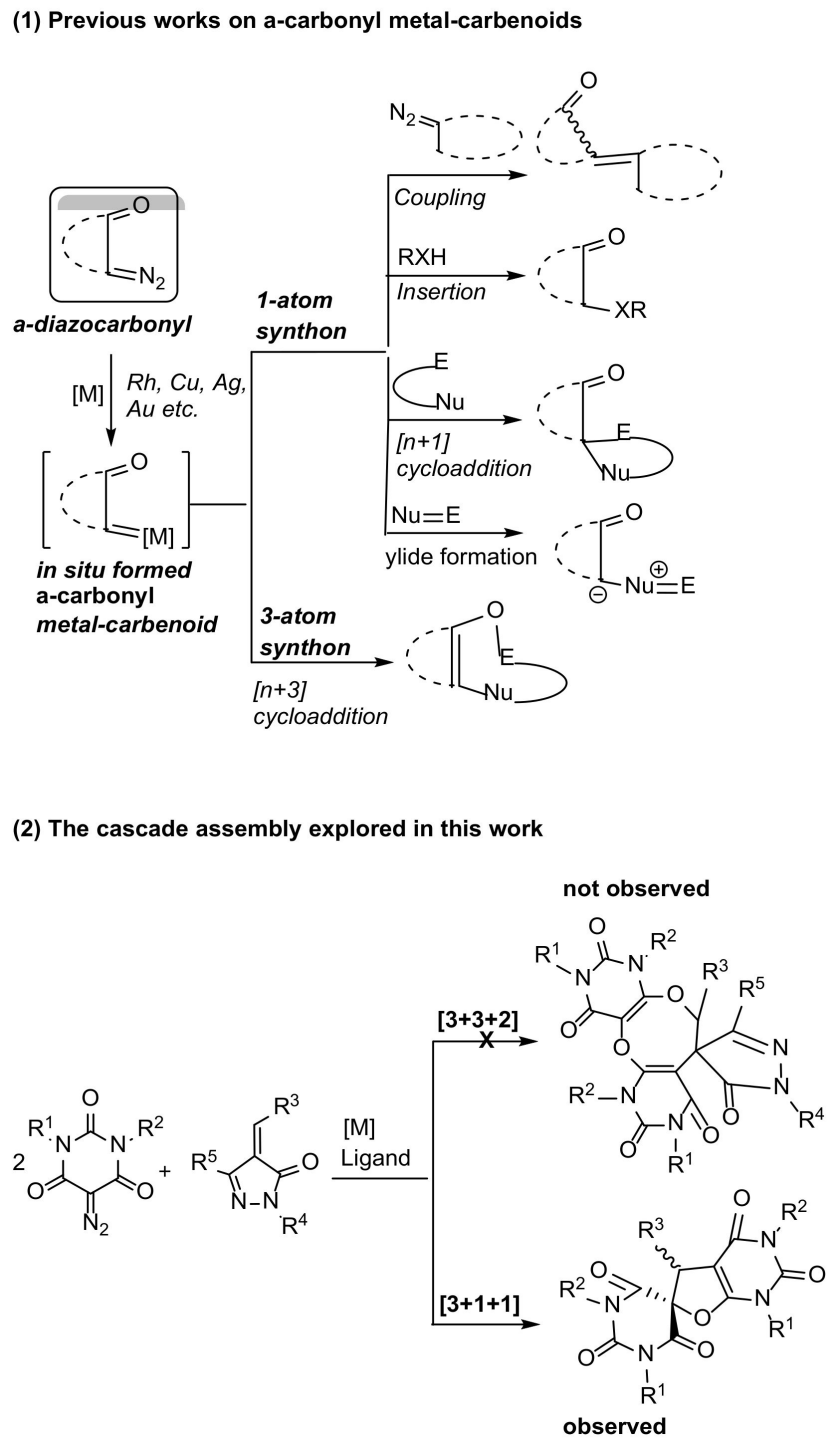
Representative synthetic methodologies of α-carbonyl metal carbenoids.

α-Diazocarbonyls represent a class of synthetically robust and versatile building blocks and by acting as typical metallocarbene precursors, they have found a broad spectrum of applications in preparing drug-like scaffolds and biologically active natural products [20,21,22,23,24,25,26,27]. Normally, treated with transition metal catalysts, α-diazocarbonyls can readily decompose into highly reactive metal carbenoids by removing N_2_ [28,29,30,31,32,33,34,35,36,37,38]. Regarding these highly reactive metal carbenoids generated in situ, they normally can behave as 1- or 3-atom synthons to perform numerous mechanistically different synthetic methodologies which often feature high chemo-, regio- and stereoselectivities Scheme 1 (1) [39,40,41,42,43,44,45,46,47,48,49,50,51]. Particularly, the in situ formed heterocycle-derived cyclic metal carbenoids are capable of functioning as 1- or 3-atom synthons to undergo the highly efficient and concise [n + 1] or [n + 3] cycloaddition with a wide range of structurally diverse and reactive organic synthons, thus delivering drug-like spiro or fused multiheterocyclic skeletons bearing structural complexities and diversities [52,53,54,55,56,57,58,59,60].

Diazobarbiturates belong to a type of structurally unique α-carbonyl diazoheterocycles and in the presence of Rh-catalysts, they can readily conduct insertion reaction, [2 + 1] cycloaddition, and [3 + 2] cycloaddition through the in situ formed α-carbonyl Rh-carbenoid synthons [61,62,63]. Nevertheless, the three-molecule assembly of barbiturate-derived Rh-carbenoids with heterocycle-based conjugated olefins has never been touched for the construction of spiro/fused furopyrimidine scaffolds. Therefore, the design and exploration of the mechanistically new three-molecule cascade assembly by employing diazobarbiturates and heterocycle-based olefins as reactants are highly urgent and needed for preparing potentially bioactive spiro/fused multiheterocyclic skeletons.

Herein, we designed and explored the transition metal-catalyzed cascade assembly of diazobarbiturates with alkylidene pyrazolones for the construction of spiro/fused multiheterocyles Scheme 1 (2). Typically, alkylidene pyrazolones exhibit plenty of chemical properties and have found a variety of applications in the preparation of structurally diverse and unique spiropyrazole analogs [64,65,66]. We found that the transition metal-catalyzed cascade assembly did not occur via the expected [3 + 3 + 2] pathway; on the contrary, it performed the unexpected [3 + 1 + 1] cascade and furnished potential bioactive spirofuropyrimidines in reasonable chemical yields. To the best of our knowledge, such a work has not been reported in the literature to date.

## 2. Results and Discussion

Initially, along with Rh_2_(OAc)_4_ (10 mol) and ligand (±)-**L1** (20 mol%) in DCE at 80 °C, we checked the ratio effects of **1a**/**2a** on the cascade assembly of diazobarbiturate **1a** with alkylidene pyrazolone **2a** (Table 1, entries 1–3). The variable ratios of **1a**/**2a** largely influenced the chemical yield of the cascade assembly. The ratio of 0.1 mmol/0.15 mmol proved to be most suitable (entries 1–2 vs. 3). Together with ligand (±)-**L1** (20 mol%) and 0.1 mmol/0.15 mmol ratio of **1a**/**2a** in DCE at 80 °C, we examined numerous structurally varying transition metal catalysts for their effects on the cascade assembly cascade (Table 1, entries 4–14). Ph_3_PAuCl, (CH_3_CN)_4_·CuBF_4_, DPPE·NiCl_2_, DPPE·PdCl_2_, and Pd(DPPE)_2_ failed to facilitate the cascade assembly (entries 4–8). Both Pd_2_(dba)_3_ and (F_3_CSO_2_)NAg afforded product **3a** in trace amounts (entries 9–10). Ru(OAc)_3_ provided **3a** in lower chemical yield (entry 11). Regarding Rh(I) and Rh(III) complexes, they were unable to catalyze the cascade assembly (entries 12–13). Delightfully, we found that Rh_2_(esp)_2_ performed efficiently to give **3a** in excellent chemical yield (entries 3–13 vs. 14). Moreover, we optimized the catalytic loading of Rh_2_(esp)_2_ and discovered that the 10 mol% loading of Rh_2_(esp)_2_ was most suitable for the cascade assembly (entries 14 vs. 15–18).

Next, in combination with Rh_2_(esp)_2_ in DCE at 80 °C, we explored several ligands for their effects on the cascade assembly of diazobarbiturate **1a** with alkylidene pyrazolone **2a** (Table 2, entries 1–7). The examined ligands significantly affected the chemical yield of the cascade assembly. Without a ligand, the cascade assembly produced product **3a** in trace amounts (entry 1). Moreover, ligand (±)-**L2** inhibited the cascade assembly (entry 2). In the case of ligands (±)-**L3**, (±)-**L4**, dppf, dppb, and (±)-**L5**, they provided product **3a** in moderate to high chemical yields (entries 3–7). Pleasantly, ligand (±)-**L1** behaved the most efficiently and delivered product **3a** in the highest chemical yield (entry 8). In the presence of Rh_2_(esp)_2_ (10 mol%) and (±)-**L1** (20 mol%) in DCE at 80 °C, we scrutinized several organic solvents for their effects on the cascade assembly and found that these organic solvents inhibited the cascade assembly from taking place (entries 9–12). Therefore, for the cascade assembly, we determined the optimal reaction conditions as below: 0.1 mmol/0.15 mmol ratio of **1a**/**2a**, 10 mol% of Rh_2_(esp)_2_, and 20 mol% of (±)-**L1** in 1,2-DCE at 80 °C. In addition, we checked several chiral ligands for their asymmetric inductions in the cascade between **1a** and **2a**, and found that in all the tested cases, product **3a** was formed without enantioselectivity (entry 13–15, see details in Supplementary Materials).

Under the well-established reaction conditions, we extended the reaction scope of the cascade assembly by diversifying diazobarbiturate **1** and alkylidene pyrazolone **2** (Table 3). The screened substrates **1** and **2** differed substantially in their reactivities and efficiencies and influenced the chemical yield of the cascade assembly significantly. In the cascade assembly with diazobarbiturate **1a**, the substrates **2a**–**2c** behaved efficiently to provide product **3a** in excellent chemical yields (entries 1–3). In contrast, the substrates **2d**, **2e**, and **2i** performed poorly and yielded their products **3** in lower chemical yields (entries 4–5 and 9). Even badly, the substrates **2f**, **2g**, and **2h** failed to react with the substrate **1a** (entries 6–8). The substrate **2a** performed the cascade assembly more efficiently than the substrates **2e**–**2i**, presumably because it utilized a phenyl as an R^3^ group (entries 1 vs. 5–9). Moreover, the substrates **2a**–**2c** containing a phenyl as the R^3^ group endured the structural variations in the R^4^ and R^5^ groups (entries 1–3). Lastly, the substrate **2d** bearing a phenyl as the R^5^ group provided product **3a** in the decreased chemical yield (entries 1 vs. 4).

Moreover, by utilizing structurally variable diazobarbiturates **1**, we checked their cascade assemblies with alkylidene pyrazolone **2c** (Table 3, entries 10–15). The substrates **1b** (R^1^ = R^2^ = cyclohexyl), **1f** (R^1^ = R^2^ = Bu^tert^), and **1g** (R^1^ = R^2^ = MeC_6_H_4_) behaved poorly and failed to deliver their product **3** (entries 10, 14–15). The substrate **1e** furnished product **3f** in a lower chemical yield (entry 13). The substrates **1c** and **1d** increased the chemical yield of their product **3** significantly (entries 11–12 vs. 10, 13–15). The substrates **1c** and **1d** with sterically less hindered the R^1^ and R^2^ groups performed more efficiently than the substrates **1b** and **1e**–**1g** with sterically more crowded R^1^ and R^2^ groups in their cascade assemblies (entry 11–12 vs. 10, 13–15). So, in the cascade assembly with alkylidene pyrazolone **2c**, the substrates **1a** and **1d** using Me or Et as the R^1^ and R^2^ groups often performed efficiently to provide products **3a** and **3e** in excellent chemical yields (entries 3 and 12).

**Figure 1:**
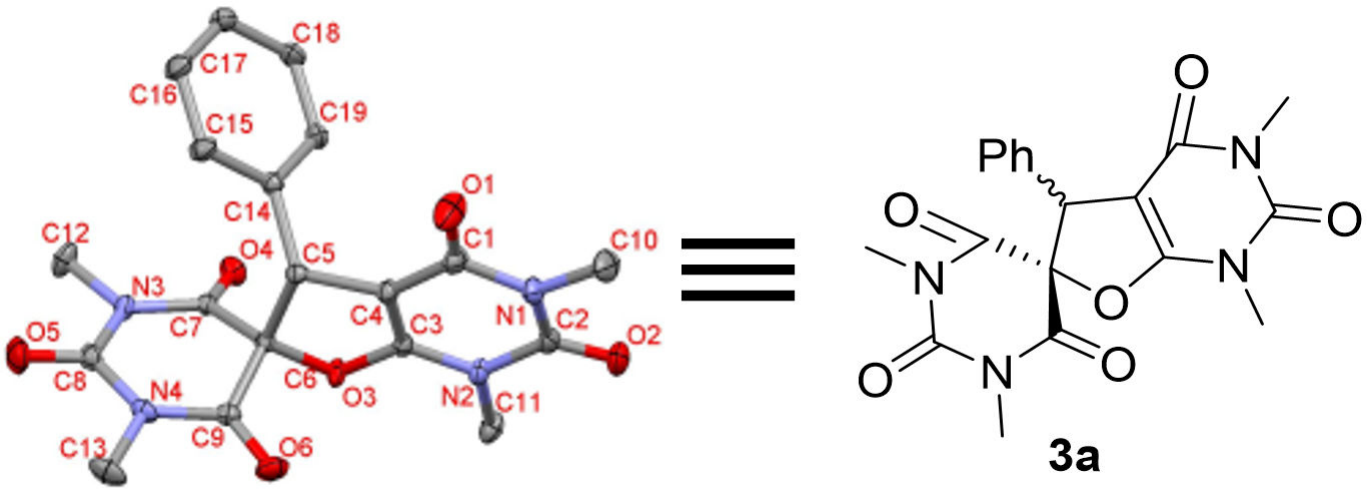
X-ray single crystal structure of **3a** (with thermal ellipsoids shown at the 50% probability level).

Meanwhile, to enrich the structural variation in product **3**, we accomplished the cascade assemblies of the substrates **1a** and **1d** with the substrates **2j**–**2r** (Table 3, 16–32). All the tested cascade assemblies exhibited desirable reactivities, thus producing their product **3** in moderate to excellent chemical yields (entries 16–32). In the cascade assemblies with the substrates **1a** and **1d**, the substrate **2** allowed the wide variation in the R^3^ group from electron-poor to electron-rich aryls. Significantly, we observed that the substitution pattern and electronic property of R^3^ affected the chemical yield of the crossed cascade assemblies (e.g., entries 16 vs. 24; 26 vs. 29). In addition, we explored the cascade assemblies of the substrate **1h** (R^1^ = Me, R^2^ = Bn) with the substrates **2a** and **2c** and these cascade assemblies were unable to take place (entries 33–34). Moreover, we determined the chemical structure of **3a** by single crystal X-ray analysis and assigned that of all the other obtained spirobarbiturates by analogy as shown in Figure 1 (CCDC 2309187) [67].

**Scheme 2:**
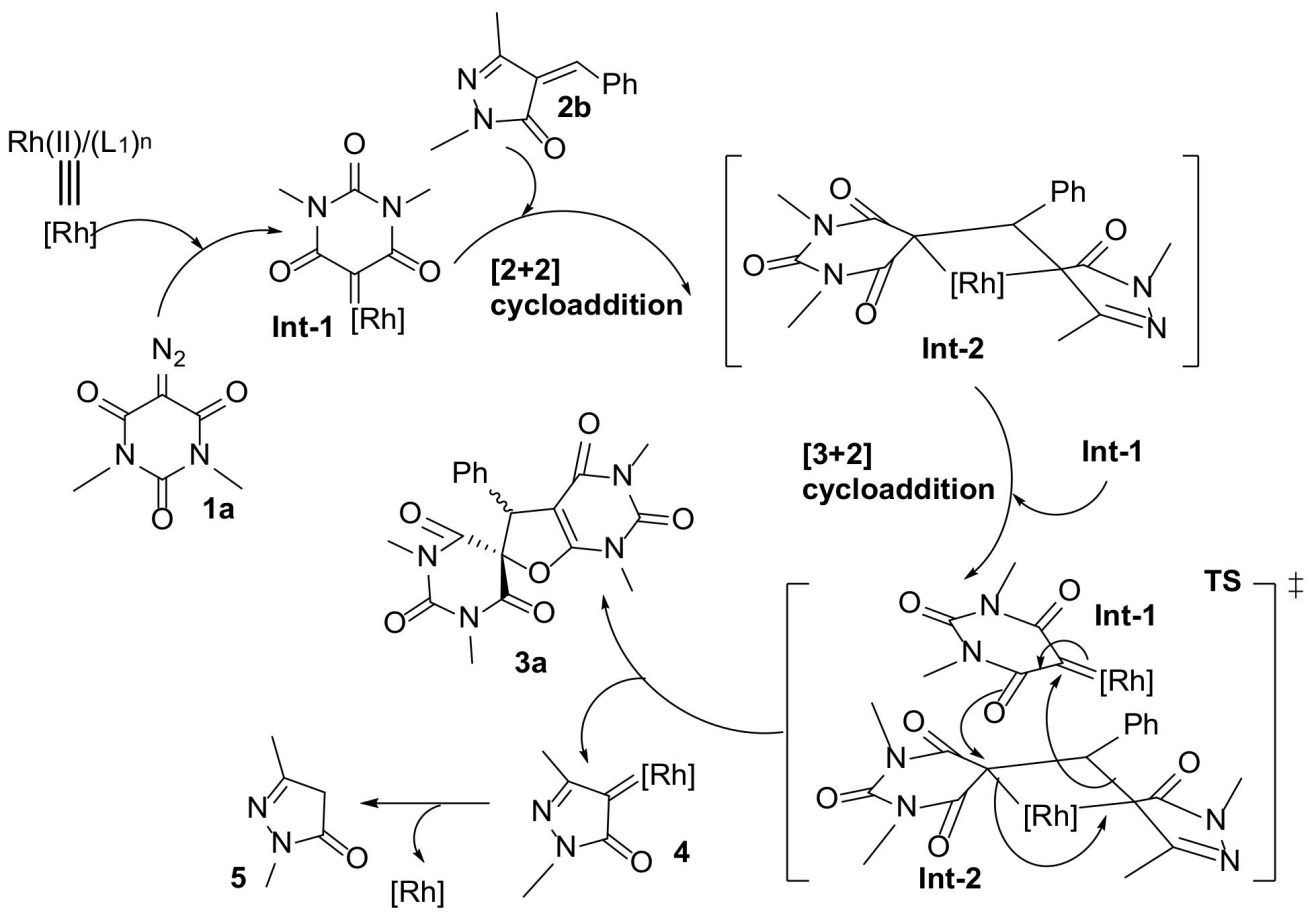
Proposed mechanism for the formation of product **3a**.

To shed light on the formation of product **3a**, on the basis of the works in the literature [68,69,70] and the LC-HRMS analysis carried out by us (see details in Supplementary Materials), we proposed the reaction mechanism for the cascade assembly of diazobarbiturate **1a** with alkylidene pyrazolone **2b** as illustrated in Scheme 2. Treated with the Rh(II)/(**L1**)n complex formed in situ, diazobarbiturate **1a** transforms into its Rh-carbenoid **Int-1**. Then, the intermediate **Int-1** performs the [**2** + **2**] cycloaddition with alkylidene pyrazolone **2b** to yield intermediate **Int-2**. Finally, via the transition state **TS**, Rh-carbenoid **Int-1** undergoes the **[3 + 2]** cycloaddition with **Int-2** to afford **3a** along with liberating Rh-carbenoid **4** which further transforms into **5**.

## 3. Materials and Methods

Proton (^1^H), carbon (^13^C), and fluorine (^19^F) NMR spectra were recorded on the Bruker (Billerica, MD, USA) Avance HD III spectrometer (400 MHz for ^1^H NMR, 100 MHz for ^13^C NMR and 376 MHz for ^19^F NMR) and calibrated using tetramethylsilane (TMS) as the internal reference. High-resolution mass spectra (HRMS) were obtained on an Agilent Technologies (Santa Clara, CA, USA) LC/MSD TOF spectrometer under electrospray ionization (ESI) conditions. The melting point of the compounds was determined by a melting point instrument. Flash column chromatography was performed on silica gel (0.035–0.070 mm) using compressed air. X-ray single crystals were obtained on the Rigaku (Wilmington, MA, USA) 002 Saturn 944 spectrometer. Thin layer chromatography (TLC) was carried out on 0.25 mm SDS silica gel-coated glass plates (60F254). Eluted plates were visualized using a 254 nm UV lamp. Unless otherwise indicated, all the reagents were commercially available and used without further purification. All the solvents were distilled from the appropriate drying agents immediately before use. Diazobarbiturates **1a**–**1g** were synthesized according to the reported procedures [61,62,71,72,73], and alkylidene pyrazolones **2a**–**2r** were prepared according to the literature procedures [74,75,76,77,78,79,80,81,82].

### 3.1. General Procedure for Cascade Assembly Reaction

A mixture of diazobarbiturate **1** (1.0 equiv, 0.1 mmol), alkylidene pyrazolone **2** (1.5 equiv, 0.15 mmol), Rh_2_(esp)_2_ (10.0 mmol%), and (±)-**L1** (20.0 mmol%) in DCE (1.5 mL) was stirred at 80 °C. After the reaction was completed as indicated by the TLC plate, the solvent was removed under reduced pressure. The resulting crude product was purified by flash column chromatography on silica gel (petroleum ether/ethyl acetate = 1:1~5:1) to afford product **3** (38–96% yields).

### 3.2. Gram-Scale Synthesis of Compound ***3a***


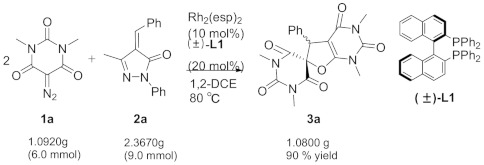


A mixture of diazobarbiturate **1a** (1.0 equiv, 6.0 mmol, 1.0920 g), alkylidene pyrazolone **2a** (1.5 equiv, 9.0 mmol, 2.3670 g), Rh_2_(esp)_2_ (10.0 mmol%, 0.4550 g), and (±)-**L1** (20.0 mmol%, 0.7440 g) in DCE (15 mL) was stirred at 80 °C. After the reaction was completed as indicated by the TLC plate, the solvent was concentrated under reduced pressure. The resulting crude product was purified by flash column chromatography on silica gel (petroleum ether/ethyl acetate = 1:1) to afford product **3a** as a white solid (1.0800 g, 90% yield).

### 3.3. Characterization of Product ***3***

1,1′,3,3′-tetramethyl-5-phenyl-1,5-dihydro-2H,2′H-spiro[furo[2,3-d]pyrimidine-6,5′-pyrimidine]-2,2′,4,4′,6′(1′H,3H,3′H)-pentaone (**3a**) [13]: white solid (yield: 18.6 mg, 93%). M.P. = 240.0–240.3 °C; ^1^H NMR (400 MHz, CDCl_3_): *δ* 7.32 (dd, *J* = 5.12, 1.72 Hz, 3H), 7.05 (dd, *J* = 5.20, 3.64 Hz, 2H), 4.91 (s, 1H), 3.50 (s, 3H), 3.40 (s, 3H), 3.28 (s, 3H), and 2.53 (s, 3H) ppm; ^13^C NMR (100 MHz, CDCl_3_): *δ* 165.5, 163.0, 162.7, 158.6, 151.2, 149.6, 132.8, 129.5, 128.9, 128.2, 90.3, 85.5, 59.2, 30.0, 29.5, 28.4, and 28.2 ppm; HRMS (ESI-TOF) *m*/*z*: [M + H]^+^ Calcd for C_19_H_19_N_4_O_6_ 399.12976; found 399.12991.

1,1′,3,3′,5-pentamethyl-1,5-dihydro-2H,2′H-spiro[furo[2,3-d]pyrimidine-6,5′-pyrimidine]-2,2′,4,4′,6′(1′H,3H,3′H)-pentaone (**3b**): white solid (yield: 6.4 mg, 38%). M.P. = 202.5–202.8 °C; ^1^H NMR (400 MHz, CDCl_3_): *δ* 3.81 (q, *J* = 6.92 Hz, 1H), 3.44 (s, 3H), 3.38 (s, 3H), 3.37 (s, 3H), 3.30 (s, 3H), and 1.33 (d, *J* = 6.88 Hz, 3H) ppm; ^13^C NMR (100 MHz, CDCl_3_): *δ* 165.8, 163.4, 161.2, 159.2, 151.1, 149.9, 88.9, 87.9, 47.8, 29.8, 29.5, 29.0, 28.0, and 15.0 ppm; HRMS (ESI-TOF) *m*/*z*: [M + H]^+^ Calcd for C_14_H_17_N_4_O_6_ 337.11435; found 337.11426.

5-(2,3-dihydrobenzofuran-5-yl)-1,1′,3,3′-tetramethyl-1,5-dihydro-2H,2′H-spiro[furo[2,3-d]pyrimidine-6,5′-pyrimidine]-2,2′,4,4′,6′(1′H,3H,3′H)-pentaone (**3c**): white solid (yield: 12.3 mg, 56%). M.P. = 265.1–265.3 °C; ^1^H NMR (400 MHz, CDCl_3_): *δ* 6.86 (s, 1H), 6.77 (d, *J* = 8.20 Hz, 1H), 6.67 (d, *J* = 8.20 Hz, 1H), 4.84 (s, 1H), 4.52 (t, *J* = 8.84 Hz, 2H), 3.48 (s, 3H), 3.37 (s, 3H), 3.26 (s, 3H), 3.13 (t, *J* = 8.64 Hz, 2H), and 2.63 (s, 3H) ppm; ^13^C NMR (100 MHz, CDCl_3_): *δ* 165.6, 163.2, 162.5, 161.1, 158.7, 151.2, 149.7, 128.3, 128.0, 124.7, 124.6, 109.5, 90.5, 85.9, 71.6,59.1, 29.9, 29.4, 29.4, 28.6, and 28.2 ppm; HRMS (ESI-TOF) *m*/*z*: [M + H]^+^ Calcd for C_21_H_21_N_4_O_7_ 441.14014; found 441.14048.

1,1′,3,3′-tetrabenzyl-5-phenyl-1,5-dihydro-2H,2′H-spiro[furo[2,3-d]pyrimidine-6,5′-pyrimidine]-2,2′,4,4′,6′(1′H,3H,3′H)-pentaone (**3d**): white solid (yield: 24.1 mg, 72%). M.P. = 112.2–112.5 °C; ^1^H NMR (400 MHz, CDCl_3_): *δ* 7.63–7.20 (m, 25H), 5.24–4.99 (m, 6H), 4.74 (s, 1H), and 4.09(q, *J* = 14.16 Hz, 2H) ppm; ^13^C NMR (100 MHz, CDCl_3_): *δ* 158.4, 150.8, 149.2, 136.9, 135.4, 134.9, 134.8, 132.2, 129.8, 129.5, 129.2, 129.1, 129.0, 128.9, 128.8, 128.6, 128.4, 128.2, 127.7, 90.4, 85.7, 59.2, 47.3,45.8, 45.7, and 44.7 ppm; HRMS (ESI-TOF) *m*/*z*: [M + H]^+^ Calcd for C_43_H_35_N_4_O_6_ 703.25720; found 703.25511.

1,1′,3,3′-tetraethyl-5-phenyl-1,5-dihydro-2H,2′H-spiro[furo[2,3-d]pyrimidine-6,5′-pyrimidine]-2,2′,4,4′,6′(1′H,3H,3′H)-pentaone (**3e**) [13]: white solid (yield: 20.7 mg, 91%). M.P. = 164.9–165.1 °C; ^1^H NMR (400 MHz, CDCl_3_): *δ* 7.35–7.31 (m, 3H), 7.10 (d, *J* = 1.88 Hz, 1H), 7.09 (d, *J* = 4.08 Hz, 1H), 4.89 (s, 1H), 4.14–3.94 (m, 6H), 3.34 (q, *J* = 7.08 Hz, 1H), 3.08 (q, *J* = 7.12 Hz, 1H), 1.44 (t, *J* = 7.12 Hz, 3H), 1.36 (t, *J* = 7.08 Hz, 3H), 1.20 (t, *J* = 7.04 Hz, 3H), and 0.68 (t, *J* = 7.12 Hz, 3H) ppm; ^13^C NMR (100 MHz, CDCl_3_): *δ* 165.5, 162.7, 162.4, 158.4, 150.4, 148.9, 132.9, 129.4, 128.9, 128.6, 89.6, 86.0, 59.1, 39.1, 38.2,37.9, 36.7, 13.8, 13.3, 13.1, and 12.4 ppm; HRMS (ESI-TOF) *m*/*z*: [M + H]^+^ Calcd for C_23_H_27_N_4_O_6_ 455.19257; found 455.19251.

1,1′,3,3′-tetraisopropyl-5-phenyl-1,5-dihydro-2H,2′H-spiro[furo[2,3-d]pyrimidine-6,5′-pyrimidine]-2,2′,4,4′,6′(1′H,3H,3′H)-pentaone (**3f**): white solid (yield: 12.0 mg, 47%). M.P. = 170.1–170.4 °C; ^1^H NMR (400 MHz, CDCl_3_): *δ* 7.31 (t, *J* = 2.12 Hz, 3H), 7.11–7.10 (m, 2H), 5.16–5.01 (m, 3H), 4.72 (s, 1H), 4.46–4.41 (m, 1H), 1.60 (dd, *J* = 7.88,4.68 Hz, 6H), 1.53 (t, *J* = 4.28 Hz, 6H), 1.41 (dd, *J* = 10.36, 4.60 Hz, 6H), and 0.83 (d, *J* = 4.36 Hz, 5H) ppm; ^13^C NMR (100 MHz, CDCl_3_): *δ* 165.9, 162.5, 158.9, 150.4, 148.8, 133.4, 129.3, 129.0, 128.8, 90.0, 87.1, 57.9, 48.5, 48.5, 48.0, 20.4, 20.4, 20.3, 19.4, 19.4, 19.1, 18.8, and 18.2 ppm; HRMS (ESI-TOF) *m*/*z*: [M + H]^+^ Calcd for C_27_H_35_N_4_O_6_ 511.25552; found 511.25511.

1,1′,3,3′-tetramethyl-5-(p-tolyl)-1,5-dihydro-2H,2′H-spiro[furo[2,3-d]pyrimidine-6,5′-pyrimidine]-2,2′,4,4′,6′(1′H,3H,3′H)-pentaone (**3g**) [13]: white solid (yield: 14.0 mg, 68%). M.P. = 141.1–141.3 °C; ^1^H NMR (400 MHz, CDCl_3_): *δ* 7.14 (d, *J* = 7.84 Hz, 2H), 6.95 (d, *J* = 8.0 Hz, 2H), 4.90 (s, 1H), 3.53 (s, 3H), 3.43 (s, 3H), 3.30 (s, 3H), 2.60 (s, 3H), and 2.32 (s, 3H) ppm; ^13^C NMR (100 MHz, CDCl_3_): *δ* 165.6, 163.1, 162.6, 158.7, 151.2, 149.6, 139.4, 129.7, 129.6, 128.1, 90.3, 85.7, 59.0, 30.0, 29.5, 28.4, 28.2, and 21.1 ppm; HRMS (ESI-TOF) *m*/*z*: [M + H]^+^ Calcd for C_20_H_21_N_4_O_6_413.14566; found 413.14556.

5-(4-bromophenyl)-1,1′,3,3′-tetramethyl-1,5-dihydro-2H,2′H-spiro[furo[2,3-d]pyrimidine-6,5′-pyrimidine]-2,2′,4,4′,6′(1′H,3H,3′H)-pentaone (**3h**): white solid (yield: 15.7 mg, 66%). M.P. = 262.0–262.4 °C; ^1^H NMR (400 MHz, CDCl_3_): *δ* 7.47(d, *J* = 8.48 Hz, 2H), 6.95 (d, *J* = 8.4 Hz, 2H), 4.88 (s, 1H), 3.51 (s, 3H), 3.42 (s, 3H), 3.29 (s, 3H), and 2.65 (s, 3H) ppm; ^13^C NMR (100 MHz, CDCl_3_): *δ* 165.3, 162.8, 162.8, 158.6, 151.1, 149.5, 132.1, 132.0, 130.0, 123.7, 89.8, 85.3, 58.3, 30.0, 29.6, 28.5, and 28.2 ppm; HRMS (ESI-TOF) *m*/*z*: [M + H]^+^ Calcd for C_19_H_18_BrN_4_O_6_ 477.04028; found 477.04042.

5-(3-chlorophenyl)-1,1′,3,3′-tetramethyl-1,5-dihydro-2H,2′H-spiro[furo[2,3-d]pyrimidine-6,5′-pyrimidine]-2,2′,4,4′,6′(1′H,3H,3′H)-pentaone (**3i**) [13]: white solid (yield: 15.6 mg, 72%). M.P. = 146.3–146.5 °C; ^1^H NMR (400 MHz, CDCl_3_): *δ* 7.35–7.33 (m, 1H), 7.29–7.26 (m, 1H), 7.06 (t, *J* = 1.76 Hz, 1H), 6.97 (d, *J* =7.56 Hz, 1H), 4.88 (s, 1H), 3.52 (s, 3H), 3.42 (s, 3H), 3.30 (s, 3H), and 2.67 (s, 3H) ppm; ^13^C NMR (100 MHz, CDCl_3_): *δ* 165.2, 163.0, 162.7, 158.5, 151.1, 149.4, 135.1, 135.0, 130.1, 129.6, 128.4, 126.5, 89.9, 85.2, 58.3, 30.0, 29.6, 28.5, and 28.2 ppm; HRMS (ESI-TOF) *m*/*z*: [M + H]^+^ Calcd for C_19_H_18_ClN_4_O_6_ 433.09106; found 433.09094.

1,1′,3,3′-tetramethyl-5-(4-(naphthalen-2-yl)phenyl)-1,5-dihydro-2H,2′H-spiro[furo[2,3-d]pyrimidine-6,5′-pyrimidine]-2,2′,4,4′,6′(1′H,3H,3′H)-pentaone (**3j**): white solid (yield: 17.5 mg, 78%). M.P. = 149.8–150.1 °C; ^1^H NMR (400 MHz, CDCl_3_): *δ* 7.82–7.78 (m, 3H), 7.57 (d, *J* = 0.68 Hz, 1H), 7.51–7.49 (m, 2H), 7.14 (q, 1H), 5.11 (s, 1H), 3.56 (s, 3H), 3.46 (s, 3H), 3.33 (s, 3H), and 2.35 (s, 3H) ppm; ^13^C NMR (100 MHz, CDCl_3_): *δ* 165.5, 163.0, 162.7, 158.7, 151.3, 149.6, 133.5, 133.0, 130.2, 128.8, 128.1, 128.0, 127.7, 127.0, 126.8, 125.1, 90.3, 85.7, 59.3, 30.0, 29.6, 28.4, and 28.3 ppm; HRMS (ESI-TOF) *m*/*z*: [M + H]^+^ Calcd for C_23_H_21_N_4_O_6_ 449.14536; found 449.14556.

1,1′,3,3′-tetramethyl-5-(4-(trifluoromethyl)phenyl)-1,5-dihydro-2H,2′H-spiro[furo[2,3-d]pyrimidine-6,5′-pyrimidine]-2,2′,4,4′,6′(1′H,3H,3′H)-pentaone (**3k**): white solid (yield: 18.6 mg, 80%). M.P. = 249.6–249.9 °C; ^1^H NMR (400 MHz, CDCl_3_): *δ* 7.61(d, *J* = 5.36 Hz, 2H), 7.23 (d, *J* = 5.36 Hz, 2H), 4.98 (s, 1H), 3.54 (s, 3H), 3.45 (s, 3H), 3.31 (s, 3H), and 2.59 (s, 3H) ppm; ^13^C NMR (100 MHz, CDCl_3_): *δ* 165.1, 162.9, 162.6, 158.6, 151.1, 149.4, 137.0, 131.8, 131.6, 128.9, 125.9, 125.9, 124.5, 122.7, 89.7, 85.1, 58.4, 30.0, 29.6, 28.4, and 28.2 ppm; ^19^F NMR (376 MHz, CDCl_3_): δ −62.9 ppm; HRMS (ESI-TOF) *m*/*z*: [M + H]^+^ Calcd for C_20_H_18_F_3_N_4_O_6_ 467.11768; found 467.11730.

5-(4-methoxyphenyl)-1,1′,3,3′-tetramethyl-1,5-dihydro-2H,2′H-spiro[furo[2,3-d]pyrimidine-6,5′-pyrimidine]-2,2′,4,4′,6′(1′H,3H,3′H)-pentaone (**3l**) [13]: white solid (yield: 10.3 mg, 48%). M.P. = 137.3–137.6 °C; ^1^H NMR (400 MHz, CDCl_3_): *δ* 6.96 (d, *J* = 5.64 Hz, 2H), 6.82 (d, *J* = 5.76 Hz, 2H), 4.87 (s, 1H), 3.76 (s, 3H), 3.49 (s, 3H), 3.39 (s, 3H), 3.27 (s, 3H), and 2.61 (s, 3H) ppm; ^13^C NMR (100 MHz, CDCl_3_): *δ* 165.6, 163.1, 162.6, 160.4, 158.7, 151.2, 149.7, 129.4, 124.6, 114.3, 90.3, 85.7, 58.8, 55.4, 29.9, 29.5, 28.5, and 28.2, ppm; HRMS (ESI-TOF) *m*/*z*: [M + H]^+^ Calcd for C_20_H_21_N_4_O_7_ 429.14038; found 429.14048.

1,1′,3,3′-tetramethyl-5-(4-nitrophenyl)-1,5-dihydro-2H,2′H-spiro[furo[2,3-d]pyrimidine-6,5′-pyrimidine]-2,2′,4,4′,6′(1′H,3H,3′H)-pentaone (**3m**) [13]: white solid (yield: 13.7 mg, 62%). M.P. = 299.5–299.7 °C; ^1^H NMR (400 MHz, DMSO): *δ* 8.16 (d, J = 5.36 Hz, 2H), 7.52 (d, *J* = 5.44 Hz, 2H), 5.36 (s, 1H), 3.42 (s, 3H), 3.23 (s, 3H), 3.13 (s, 3H), and 2.51 (s, 3H) ppm; ^13^C NMR (100 MHz, DMSO): *δ* 165.6, 163.3, 163.2, 158.6, 151.3, 150.4, 148.0, 142.9, 130.8, 123.5, 90.0, 85.8, 55.0, 30.3, 29.6, 28.3, and 28.2 ppm; HRMS (ESI-TOF) *m*/*z*: [M + H]^+^ Calcd for C_19_H_18_N_5_O_8_ 444.11469; found 444.11499.

5-(2-bromophenyl)-1,1′,3,3′-tetramethyl-1,5-dihydro-2H,2′H-spiro[furo[2,3-d]pyrimidine-6,5′-pyrimidine]-2,2′,4,4′,6′(1′H,3H,3′H)-pentaone (**3n**): white solid (yield: 15.7 mg, 66%). M.P. = 254.4–254.8 °C; ^1^H NMR (400 MHz, CDCl_3_): *δ* 7.57 (d, *J* = 5.32 HZ, 1H), 7.33 (t, *J* = 5.04 HZ,1H), 7.22–7.16 (m, 2H), 5.58 (s, 1H), 3.52 (s, 3H), 3.40 (s, 3H), 3.32 (s, 3H), and 2.71 (s, 3H) ppm; ^13^C NMR (100 MHz, CDCl_3_): *δ* 165.1, 163.1, 162.7, 158.4, 151.2, 149.5, 132.9, 132.4, 131.0, 130.8, 128.0, 124.4, 88.8, 86.1, 56.8, 30.0, 29.5, 28.5, and 28.2 ppm; HRMS (ESI-TOF) *m*/*z*: [M + H]^+^ Calcd for C_19_H_18_BrN_4_O_6_ 477.04086; found 477.04042.

1,1′,3,3′-tetramethyl-5-(m-tolyl)-1,5-dihydro-2H,2′H-spiro[furo[2,3-d]pyrimidine-6,5′-pyrimidine]-2,2′,4,4′,6′(1′H,3H,3′H)-pentaone (**3o**): white solid (yield: 18.5 mg, 90%). M.P. = 135.7–135.9 °C; ^1^H NMR (400 MHz, CDCl_3_): *δ* 7.25–7.15 (m, 2H), 6.86 (d, *J* = 6.32 Hz, 2H), 4.89 (s, 1H), 3.54 (s, 3H), 3.44 (s, 3H), 3.32 (s, 3H), 2.58 (s, 3H), and 2.32 (s, 3H) ppm; ^13^C NMR (100 MHz, CDCl_3_): *δ* 165.5, 163.0, 162.6, 158.6, 151.2, 149.6, 138.8, 132.7, 130.2, 128.8, 128.8, 125.3, 90.4, 85.6, 59.3, 30.0, 29.7, 29.5, 28.4, 28.2, and 21.3 ppm; HRMS (ESI-TOF) *m*/*z*: [M + H]^+^ Calcd for C_20_H_21_N_4_O_6_ 413.14529; found 413.14556.

1,1′,3,3′-tetraethyl-5-(p-tolyl)-1,5-dihydro-2H,2′H-spiro[furo[2,3-d]pyrimidine-6,5′-pyrimidine]-2,2′,4,4′,6′(1′H,3H,3′H)-pentaone (**3p**) [13]: white solid (yield: 19.2 mg, 82%). M.P. = 134.9–135.2 °C; ^1^H NMR (400 MHz, CDCl_3_): *δ* 7.12 (d, *J* = 5.08 Hz, 2H), 6.97 (d, *J* = 5.08 Hz, 2H), 4.85 (s, 1H), 4.12–3.94 (m, 6H), 3.36 (q, *J* = 4.40 Hz, 1H), 3.10 (q, *J* = 4.40 Hz, 1H), 2.31 (s, 3H), 1.43 (t, *J* = 4.72 Hz, 3H), 1.34 (t, *J* = 4.72 Hz, 3H), 1.20 (t, *J* = 4.64 Hz, 3H), and 0.69 (t, *J* = 4.72 Hz, 3H) ppm; ^13^C NMR (100 MHz, CDCl_3_): *δ* 165.5, 162.8, 162.3, 158.4, 150.4, 148.9, 139.4, 129.8, 129.6, 128.4, 89.7, 86.1, 58.9, 39.0, 38.2, 37.9, 36.7, 29.7, 21.1, 13.8, 13.3, 13.1, and 12.3 ppm; HRMS (ESI-TOF) *m*/*z*: [M + H]^+^ Calcd for C_24_H_29_N_4_O_6_ 469.20752; found 469.20816.

5-(4-bromophenyl)-1,1′,3,3′-tetraethyl-1,5-dihydro-2H,2′H-spiro[furo[2,3-d]pyrimidine-6,5′-pyrimidine]-2,2′,4,4′,6′(1′H,3H,3′H)-pentaone (**3q**): white solid (yield: 25.3 mg, 95%). M.P. = 150.1–150.2 °C; ^1^H NMR (400 MHz, CDCl_3_): *δ* 7.46 (d, *J* = 8.48 Hz, 2H), 6.97 (d, *J* = 8.40 Hz, 2H), 4.82 (s, 1H), 4.12–3.91 (m, 6H), 3.42 (q, *J* = 7.08 Hz, 1H), 3.16 (q, *J* = 7.12 Hz, 1H), 1.42 (t, *J* = 7.12 Hz, 3H), 1.33 (t, *J* = 7.04 Hz, 3H), 1.19 (t, *J* = 7.04 Hz, 3H), and 0.73 (t, *J* = 7.12 Hz, 3H) ppm; ^13^C NMR (100 MHz, CDCl_3_): *δ* 165.2, 162.5, 158.3, 150.3, 148.8, 132.1, 132.0, 130.2, 123.7, 89.2, 85.7, 58.3, 39.1, 38.3, 38.0, 36.8, 13.8, 13.3, 13.0, and 12.4 ppm; HRMS (ESI-TOF) *m*/*z*: [M + H]^+^ Calcd for C_23_H_26_BrN_4_O_6_ 533.10266; found 533.10302.

5-(3-chlorophenyl)-1,1′,3,3′-tetraethyl-1,5-dihydro-2H,2′H-spiro[furo[2,3-d]pyrimidine-6,5′-pyrimidine]-2,2′,4,4′,6′(1′H,3H,3′H)-pentaone (**3r**): white solid (yield: 22.7 mg, 93%). M.P. = 179.6–179.8 °C; ^1^H NMR (400 MHz, CDCl_3_): *δ* 7.34–7.25 (m, 2H), 7.08 (t, *J* = 1.72 Hz, 1H), 7.00 (d, *J* = 7.52 Hz, 1H), 4.84 (s, 1H), 4.14–3.94 (m, 6H), 3.44 (q, *J* = 7.08 Hz, 1H), 3.20 (q, *J* = 7.12 Hz, 1H), 1.44 (t, *J* = 7.12 Hz, 3H), 1.35 (t, *J* = 7.04 Hz, 3H), 1.21 (t, *J* = 7.04 Hz, 3H), and 0.73 (t, *J* = 7.12 Hz, 3H) ppm; ^13^C NMR (100 MHz, CDCl_3_): *δ* 165.2, 162.6, 162.4, 158.3, 150.3, 148.8, 135.1, 135.0, 130.2, 129.6, 128.8, 126.8, 89.3, 85.6, 58.4, 39.1, 38.3, 38.0, 36.8, 13.8, 13.3, 13.0, and 12.4 ppm; HRMS (ESI-TOF) *m*/*z*: [M + H]^+^ Calcd for C_23_H_26_ClN_4_O_6_ 489.15314; found 489.15354.

1,1′,3,3′-tetraethyl-5-(4-(naphthalen-2-yl)phenyl)-1,5-dihydro-2H,2′H-spiro[furo[2,3-d]pyrimidine-6,5′-pyrimidine]-2,2′,4,4′,6′(1′H,3H,3′H)-pentaone (**3s**): white solid (yield: 20.2 mg, 80%). M.P. = 189.5–189.7 °C; ^1^H NMR (400 MHz, CDCl_3_): *δ* 7.82–7.77 (m, 3H), 7.58 (s, 1H), 7.52–7.47 (m, 2H), 7.18 (dd, *J* = 8.44, 1.60 Hz, 1H), 5.08 (s, 1H), 4.18–3.95 (m, 6H), 3.15 (q, *J* = 7.08 Hz, 1H), 2.91 (q, *J* = 7.04 Hz, 1H), 1.47 (t, *J* = 7.08 Hz, 3H), 1.40 (t, *J* = 7.08 Hz, 3H), 1.22 (t, *J* = 7.04 Hz, 3H), and 0.40 (t, *J* = 7.04 Hz, 3H) ppm; ^13^C NMR (100 MHz, CDCl_3_): *δ* 165.5, 162.8, 162.5, 158.5, 150.5, 148.9, 133.6, 133.1, 130.3, 128.8, 128.3, 128.0, 127.6, 126.8, 126.7, 125.4, 89.7, 86.1, 59.3, 39.1, 38.3, 37.8, 36.8, 13.8, 13.3, 13.1, and 12.1 ppm; HRMS (ESI-TOF) *m*/*z*: [M + H]^+^ Calcd for C_27_H_29_N_4_O_6_ 505.20953; found 505.20816.

1,1′,3,3′-tetraethyl-5-(4-methoxyphenyl)-1,5-dihydro-2H,2′H-spiro[furo[2,3-d]pyrimidine-6,5′-pyrimidine]-2,2′,4,4′,6′(1′H,3H,3′H)-pentaone (**3t**): white solid (yield: 15.7 mg, 65%). M.P. = 119.2–119.6 °C; ^1^H NMR (400 MHz, (CD_3_)_2_CO): *δ* 7.11 (d, *J* = 5.68 Hz, 2H), 6.87 (d, *J* = 5.84 Hz, 2H), 4.94 (s, 1H), 4.06–3.86 (m, 6H), 3.79 (s, 3H), 3.39 (q, *J* = 4.28 Hz, 1H), 3.09 (q, *J* = 4.24 Hz, 1H), 1.38 (t, *J* = 4.72 Hz, 3H), 1.31 (t, *J* = 1.76 Hz, 3H), 1.12 (t, *J* = 4.68 Hz, 3H), and 0.71 (t, *J* = 4.72 Hz, 3H) ppm; ^13^C NMR (100 MHz, CDCl_3_): *δ* 165.5, 162.9, 162.2, 160.4, 158.4, 150.4, 148.9, 129.7, 124.7, 114.3, 89.8, 86.2, 58.6, 55.3, 39.0, 38.2, 37.9, 36.7, 13.8, 13.3, 13.1, and 12.5 ppm; HRMS (ESI-TOF) *m*/*z*: [M + H]^+^ Calcd for C_24_H_29_N_4_O_7_ 485.20370; found 485.20308.

1,1′,3,3′-tetraethyl-5-(4-nitrophenyl)-1,5-dihydro-2H,2′H-spiro[furo[2,3-d]pyrimidine-6,5′-pyrimidine]-2,2′,4,4′,6′(1′H,3H,3′H)-pentaone (**3u**) [13]: yellow solid (yield: 22.5 mg, 90%). M.P. = 207.7–207.8 °C; ^1^H NMR (400 MHz, CDCl_3_): *δ* 8.18 (d, *J* = 8.64Hz, 2H), 7.30 (d, *J* = 8.56 Hz, 2H), 4.94 (s, 1H), 4.12–3.91 (m, 6H), 3.40 (q, *J* = 6.32 Hz, 1H), 3.13 (q, *J* = 6.32 Hz, 1H), 1.43 (t, *J* = 7.12 Hz, 3H), 1.35 (t, *J* = 7.04 Hz, 3H), 1.19 (t, *J* = 6.96 Hz, 3H), and 0.68 (t, *J* = 7.04 Hz, 3H) ppm; ^13^C NMR (100 MHz, CDCl_3_): *δ* 164.9, 162.8, 162.2, 158.3, 150.2, 148.5, 148.4, 140.3, 129.9, 123.9, 88.8, 85.5, 57.8, 39.2, 38.5, 38.0, 36.8, 13.8, 13.3, 13.0, and 12.4 ppm; HRMS (ESI-TOF) *m*/*z*: [M + H]^+^ Calcd for C_23_H_26_N_5_O_8_ 500.17752; found 500.17759.

5-(2-bromophenyl)-1,1′,3,3′-tetraethyl-1,5-dihydro-2H,2′H-spiro[furo[2,3-d]pyrimidine-6,5′-pyrimidine]-2,2′,4,4′,6′(1′H,3H,3′H)-pentaone (**3v**): white solid (yield: 14.9 mg, 56%). M.P. = 164.8–164.9 °C; ^1^H NMR (400 MHz, CDCl_3_): *δ* 7.52 (d, *J* = 7.64 Hz, 1H), 7.34–7.28 (m, 1H), 7.18 (d, *J* = 6.92 Hz, 2H), 5.52 (s, 1H), 4.08–3.93 (m, 6H), 3.58 (q, *J* = 6.32 Hz, 1H), 3.26 (q, *J* = 6.32 Hz, 1H), 1.42 (t, *J* =7.00 Hz, 3H), 1.33 (t, *J* = 7.16 Hz, 3H), 1.21 (t, *J* = 7.00 Hz, 3H), and 0.73 (t, *J* = 7.08 Hz, 3H) ppm; ^13^C NMR (100 MHz, CDCl_3_): *δ* 165.2, 162.6, 162.4, 158.2, 150.4, 148.9, 132.8, 132.8, 131.5, 130.6, 127.9, 124.8, 88.1, 86.8, 56.3, 39.1, 38.6, 37.9, 36.8, 13.8, 13.1, and 12.3 ppm; HRMS (ESI-TOF) *m*/*z*: [M + H]^+^ Calcd for C_23_H_26_BrN_4_O_6_ 533.10181; found 533.10302.

1,1′,3,3′-tetraethyl-5-(m-tolyl)-1,5-dihydro-2H,2′H-spiro[furo[2,3-d]pyrimidine-6,5′-pyrimidine]-2,2′,4,4′,6′(1′H,3H,3′H)-pentaone (**3w**): white solid (yield: 17.1 mg, 73%). M.P. = 169.3–169.6 °C; ^1^H NMR (400 MHz, CDCl_3_): *δ* 7.22–7.12 (m, 2H), 6.88 (d, *J* = 6.80 Hz, 2H), 4.84 (s, 1H), 4.11–3.93 (m, 6H), 3.34 (q, *J* = 6.44 Hz, 1H), 3.09 (q, *J* = 6.40 Hz, 1H), 2.30 (s, 3H), 1.43 (t, *J* = 7.08 Hz, 3H), 1.35 (t, *J* = 7.00 Hz, 3H), 1.20 (t, *J* = 6.96 Hz, 3H), and 0.68 (t, *J* = 7.04 Hz, 3H) ppm; ^13^C NMR (100 MHz, CDCl_3_): *δ* 165.5, 162.8, 162.4, 158.4, 150.4, 148.9, 138.6, 132.7, 130.2, 129.2, 128.8, 125.6, 89.8, 86.0, 59.1, 39.1, 38.2, 37.9, 36.7, 21.3, 13.8, 13.3, 13.1, and 12.3 ppm; HRMS (ESI-TOF) *m*/*z*: [M + H]^+^ Calcd for C_24_H_29_N_4_O_6_ 469.20792; found 469.20816.

## 4. Conclusions

Conclusively, under the catalysis of Rh_2_(esp)_2_ and (±)-BINAP in DCE at 80 °C, the cascade assembly between diazobarbiturates and alkylidene pyrazolones proceeds readily and delivers the spirofuropyrimidines in reasonable chemical yields. Moreover, the design and exploration of the other cascade assemblies of α-diazocarbonyl metal carbenoids are ongoing in our organic lab and will be reported in due course.

## Figures and Tables

**Table 1:** Screening of **1a**/**2a** ratios and transition metal catalysts ^[a]^.

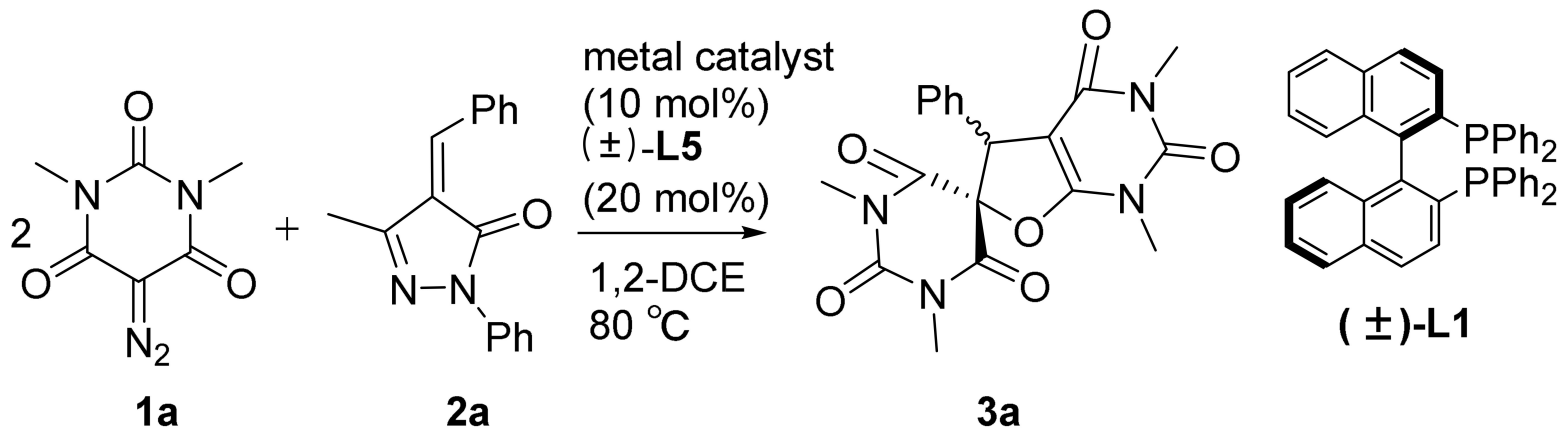			
Entry	[M] ^[f]^	Time (h)	Yield ^[b]^ (%)
1 ^[c]^	Rh_2_(OAc)_4_	6	40
2 ^[d]^	Rh_2_(OAc)_4_	6	65
3	Rh_2_(OAc)_4_	6	75
4	Ph_3_PAuCl	6	NR ^[e]^
5	(CH_3_CN)_4_·CuBF_4_	6	NR ^[e]^
6	DPPE·NiCl_2_	8	NR ^[e]^
7	DPPE·PdCl_2_	8	NR ^[e]^
8	Pd(DPPE)_2_	8	NR ^[e]^
9	Pd_2_(dba)_3_	6	trace
10	(F_3_CSO_2_)NAg	6	trace
11	Ru(OAc)_3_	6	21
12	[Rh(COD)_2_]BF_4_	6	NR ^[e]^
13	[Rh_3_O(OAc)_6_(H_2_O)_3_]OAc	6	NR ^[e]^
14	Rh_2_(esp)_2_	6	93
15 ^[g]^	Rh_2_(esp)_2_	6	25
16 ^[h]^	Rh_2_(esp)_2_	6	16
17 ^[i]^	Rh_2_(esp)_2_	6	trace
18 ^[j]^	Rh_2_(esp)_2_	6	NR ^[e]^

^[a]^ reactions were carried out with **1a** (0.1 mmol), **2a** (0.15 mmol), catalyst (10 mol%, 0.01 mmol), and (±)-**L1** (20 mol%, 0.02 mmol) in 1,2-DCE (1.5 mL) at 80 °C. ^[b]^ isolated yield. ^[c]^ **1a**/**2a** = 0.15 mmol/0.1 mmol. ^[d]^ **1a**/**2a** = 0.1 mmol/0.1 mmol. ^[e]^ no reaction. ^[f]^ Rh_2_(OAc)_4_: Bis[rhodium(α,α,α′,α′-tetramethyl-1,3-benzenedipropionic acid); Ph_3_PAuCl: Chloro(triphenylphosphine)gold; (CH_3_CN)_4_·CuBF_4_: Tetrakis(acetonitrile)copper(I) tetrafluoroborate; DPPE·NiCl_2_: 1,2-Bis(diphenylphosphino)ethane nickel(II) chloride; DPPE·PdCl_2_: [1,2-Bis(diphenylphosphino)ethane]dichloropalladium(II); Pd(DPPE)_2_: Bis[1,2-bis(diphenylphosphino)ethane]palladium(0); Pd_2_(dba)_3_: Tris(dibenzylideneacetone)dipalladium; (F_3_CSO_2_)NAg: Sliver bis(trifluoromethane sulfonimide); Ru(OAc)_3_: Ruthenium acetate; [Rh(COD)_2_]BF_4_: Bis(1,5-cyclooctadiene)rhodium(I) tetrafluoroborate; [Rh_3_O(OAc)_6_(H_2_O)_3_]OAc: Rhodium acetate; Rh_2_(esp)_2_: Bis[rhodium(α,α,α′,α′-tetramethyl-1,3-benzenedipropionic acid). ^[g]^ 20 mol% Rh_2_(esp)_2_ and 40 mol% (±)-**L1**. ^[h]^ 5 mol% Rh_2_(esp)_2_ and 10 mol% (±)-**L1**. ^[i]^ 2.5 mol% Rh_2_(esp)_2_ and 5 mol% (±)-**L1**. ^[j]^ 1.25 mol% Rh_2_(esp)_2_ and 2.5 mol% (±)-**L1**.

**Table 2:** Screening of ligands and solvents ^[a]^.

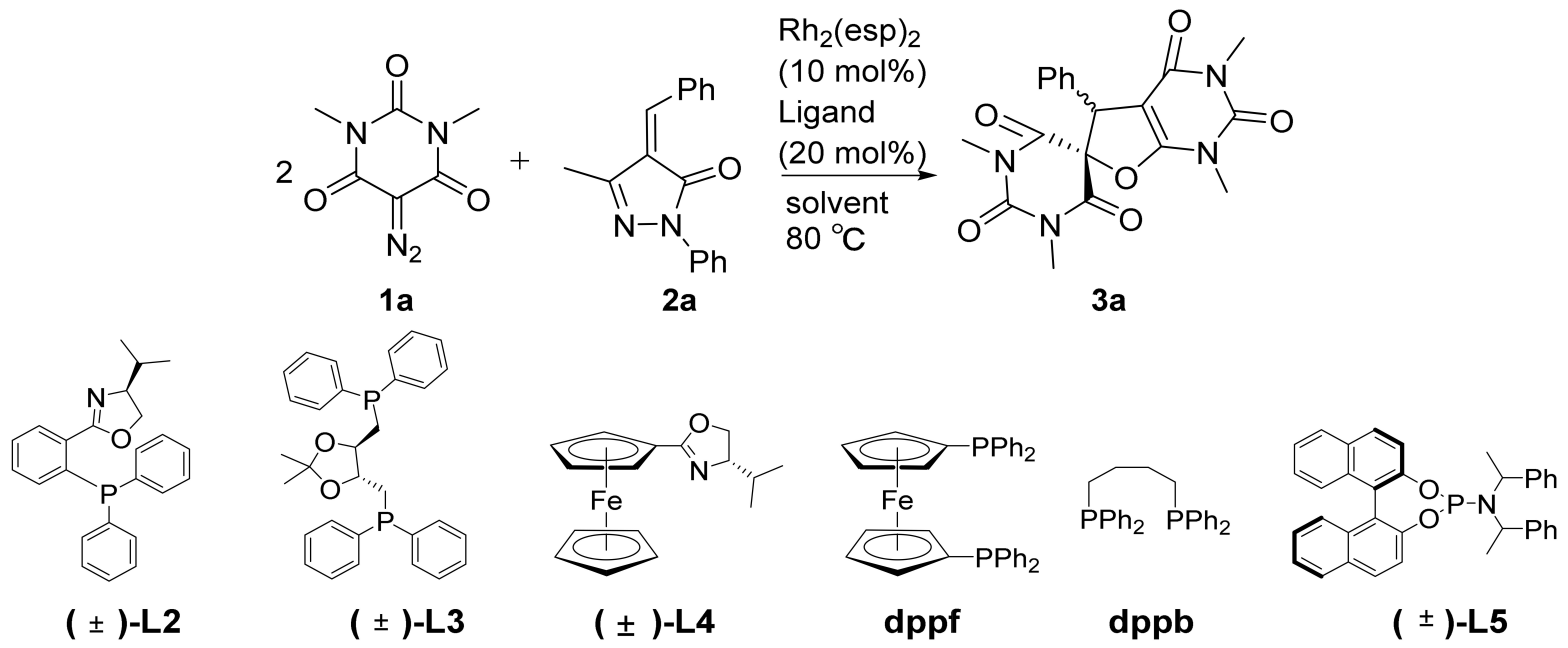					
Entry	Ligand	Solvent	Time (h)	Yield ^[b]^ (%)	ee ^[d]^ (%)
1	**-**	1,2-DCE	6	trace	-
2	(±)**-L2**	1,2-DCE	6	NR ^[c]^	-
3	(±)**-L3**	1,2-DCE	6	45	-
4	(±)**-L4**	1,2-DCE	6	67	-
5	dppf	1,2-DCE	6	68	-
6	dppb	1,2-DCE	6	77	-
7	(±)**-L5**	1,2-DCE	8	82	-
8	(±)**-L1**	1,2-DCE	6	93	-
9	(±)**-L1**	PhCF_3_	6	NR ^[c]^	-
10	(±)**-L1**	HFIP	6	NR ^[c]^	-
11	(±)**-L1**	C_6_F_6_	6	NR ^[c]^	-
12	(±)**-L1**	CHCl_3_	6	NR ^[c]^	-
13	(*S*)**-L2**	1,2-DCE	6	45	0
14	(*R*, *R*)**-L3**	1,2-DCE	6	67	0
15	(*R*)**-L5**	1,2-DCE	6	93	0

^[a]^ reactions were carried out with **1a** (0.1 mmol), **2a** (0.15 mmol), Rh_2_(esp)_2_ (10 mol%, 0.01 mmol), and ligand (20 mol%, 0.02 mmol) in solvent (1.5 mL) at 80 °C. ^[b]^ isolated yield. ^[c]^ no reaction. ^[d]^ Determined by chiral HPLC analysis.

**Table 3:** Substrate scope ^[a]^.

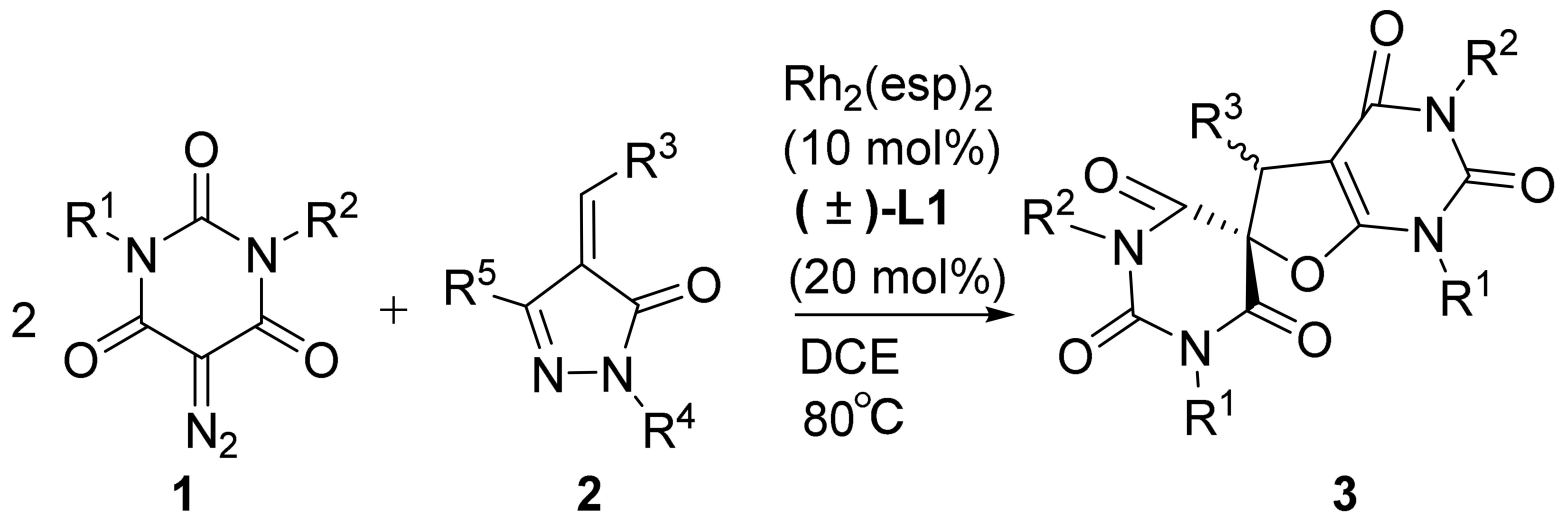					
Entry	1 (R^1^, R^2^)	2 (R^3^, R^4^, R^5^)	3	Time (h)	Yield ^[b]^ (%)
1	**1a** (Me, Me)	**2a** (Ph, Ph, Me)	**3a**	6	93
2	**1a** (Me, Me)	**2b** (Ph, Me, Me)	**3a**	6	86
3	**1a** (Me, Me)	**2c** (Ph, Me, H)	**3a**	6	96
4	**1a** (Me, Me)	**2d** (Ph, Ph, Ph)	**3a**	6	57
5	**1a** (Me, Me)	**2e** (Me, Ph, Me)	**3b**	6	38
6	**1a** (Me, Me)	**2f** (Pr*^iso^*, Ph, Me)	-	6	NR ^[c]^
7	**1a** (Me, Me)	**2g** 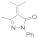	-	6	NR ^[c]^
8	**1a** (Me, Me)	**2h** (2-thiophenyl, Ph, Me)	-	6	NR ^[c]^
9	**1a** (Me, Me)	**2i** (5-benzofuranyl, Ph, Me)	**3c**	6	56
10	**1b** (cyclohexyl, cyclohexyl)	**2c** (Ph, Me, H)	-	6	NR ^[c]^
11	**1c** (Bn, Bn)	**2c** (Ph, Me, H)	**3d**	6	88
12	**1d** (Et, Et)	**2c** (Ph, Me, H)	**3e**	6	91
13	**1e** (Pr*^iso^*, Pr*^iso^*)	**2c** (Ph, Me, H)	**3f**	6	47
14	**1f** (Bu*^tert^*, Bu*^tert^*)	**2c** (Ph, Me, H)	-	6	NR ^[c]^
15	**1g** (4-MeC_6_H_4_, 4-MeC_6_H_4_)	**2c** (Ph, Me, H)	-	6	NR ^[c]^
16	**1a** (Me, Me)	**2j** (4-MeC_6_H_4_, Ph, Me)	**3g**	3	68
17	**1a** (Me, Me)	**2k** (4-BrC_6_H_4_, Ph, Me)	**3h**	3	66
18	**1a** (Me, Me)	**2l** (3-ClC_6_H_4_, Ph, Me)	**3i**	3	72
19	**1a** (Me, Me)	**2m** (2-naphthyl, Ph, Me)	**3j**	6	78
20	**1a** (Me, Me)	**2n** (4- F_3_CC_6_H_4_, Ph, Me)	**3k**	6	80
21	**1a** (Me, Me)	**2o** (4-MeOC_6_H_4_, Ph, Me)	**3l**	3	48
22	**1a** (Me, Me)	**2p** (4-O_2_NC_6_H_4_, Ph, Me)	**3m**	6	62
23	**1a** (Me, Me)	**2q** (2-BrC_6_H_4_, Ph, Me)	**3n**	6	66
24	**1a** (Me, Me)	**2r** (3-MeC_6_H_4_, Ph, Me)	**3o**	6	90
25	**1d** (Et, Et)	**2j** (4-MeC_6_H_4_, Ph, Me)	**3p**	6	82
26	**1d** (Et, Et)	**2k** (4-BrC_6_H_4_, Ph, Me)	**3q**	6	95
27	**1d** (Et, Et)	**2l** (3-ClC_6_H_4_, Ph, Me)	**3r**	6	93
28	**1d** (Et, Et)	**2m** (2-naphthyl, Ph, Me)	**3s**	6	80
29	**1d** (Et, Et)	**2o** (4-MeOC_6_H_4_, Ph, Me)	**3t**	6	65
30	**1d** (Et, Et)	**2p** (4-O_2_NC_6_H_4_, Ph, Me)	**3u**	6	90
31	**1d** (Et, Et)	**2q** (2-BrC_6_H_4_, Ph, Me)	**3v**	6	63
32	**1d** (Et, Et)	**2r** (3-MeC_6_H_4_, Ph, Me)	**3w**	6	73
33	**1h** (Me,Bn)	**2a** (Ph, Ph, Me)	-	6	NR ^[c]^
34	**1h** (Me, Bn)	**2c** (Ph, Me, H)	-	6	NR ^[c]^

^[a]^ reactions were carried out with **1a** (0.1 mmol), **2a** (0.15 mmol), Rh_2_(esp)_2_ (10 mol%,0.01 mmol), and (±)-**L1**(20 mol%, 0.02 mmol) in 1,2-DCE (1.5 mL) at 80 °C. ^[b]^ isolated chemical yield. ^[c]^ no reaction.

## Data Availability

The original contributions presented in the study are included in the article.

## Supplementary Materials

The following supporting information can be downloaded at: https://www.mdpi.com/article/10.3390/molecules29133178/s1, NMR spectral copies of products **3**. X-ray crystal data of product **3a**.
