# Supplementary material for: α-Carbonyl Rh-Carbenoid Initiated Cascade Assembly of Diazobarbiturates with Alkylidene Pyrazolones for Synthesis of Spirofuropyrimidines
Source: Molecules. 2024 Jul 3;29(13):3178. doi: 10.3390/molecules29133178 (PMC11243257; doi:10.3390/molecules29133178)
Supplement: Supplementary file 1 [file molecules-29-03178-s001.zip › molecules-3081957-supplementary.pdf]

Supporting Information

α-Carbonyl Rh-Carbenoid Initiated Cascade Assembly of Diazobarbiturates with Alkylidene Pyrazolones for Synthesis of Spirofuropyrimidines

Yue Zhang, Yu-Hang Mi, Kuo Wang and Hong-Wu Zhao

Table of Contents

Supporting Information S1

Table of Contents S1

1. NMR Spectra of Products **3** S2

2. X-Ray Crystal Data of Product **3a** S28

3. Copies of HPLC Spectra of Product **3a** S30

4. Reaction Mechanism Investigation by LC-HRMS Analysis S32

1. NMR Spectra of Products 3

1,1',3,3'-tetramethyl-5-phenyl-1,5-dihydro-2H,2'H-spiro[furo[2,3-d]pyrimidine-6, 5'-pyrimidine]-2,2',4,4',6'(1'H,3H,3'H)-pentaone (3a):

^{1}H NMR (400MHz,$CDCl_3$) of 3a

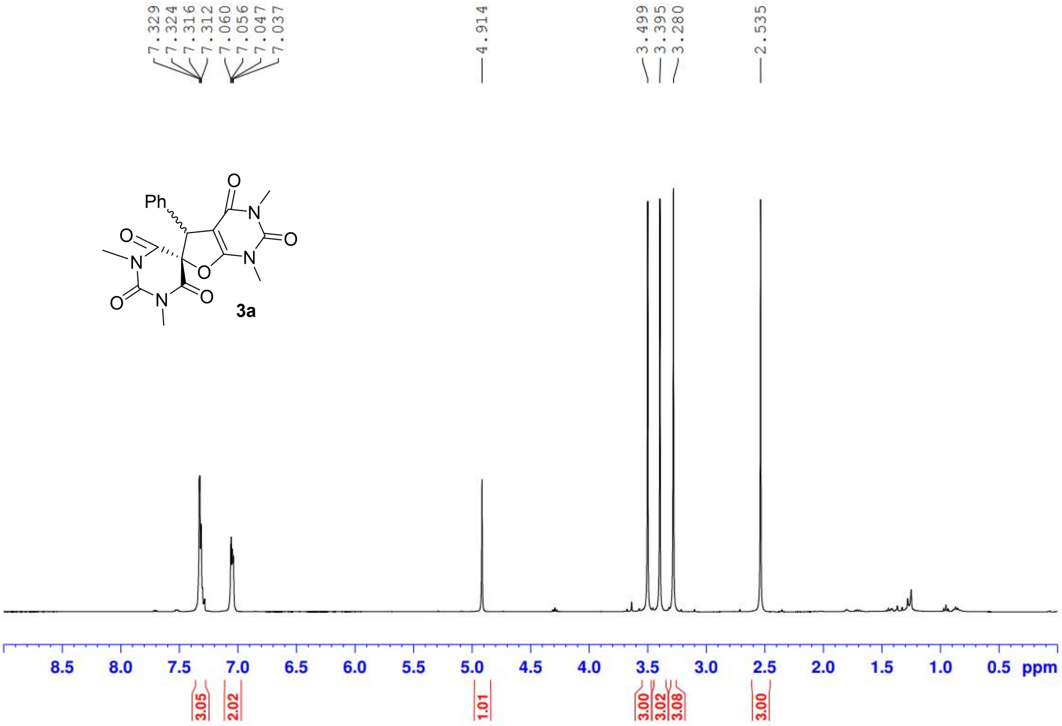

^{13}C NMR (100MHz,$CDCl_3$) of 3a

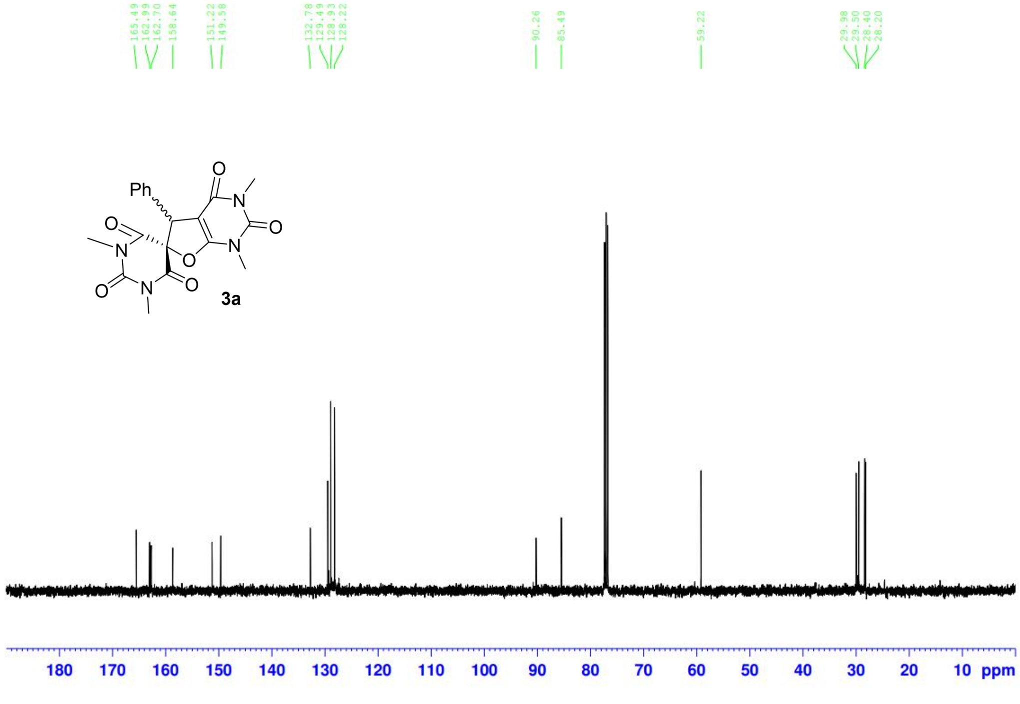

1,1',3,3',5-pentamethyl-1,5-dihydro-2H,2'H-spiro[furo[2,3-d]pyrimidine-6,5'-pyrimidine]-2,2',4,4',6'(1'H,3H,3'H)-pentaone(3b):

^{1}H NMR (400MHz,$CDCl_3$) of 3b

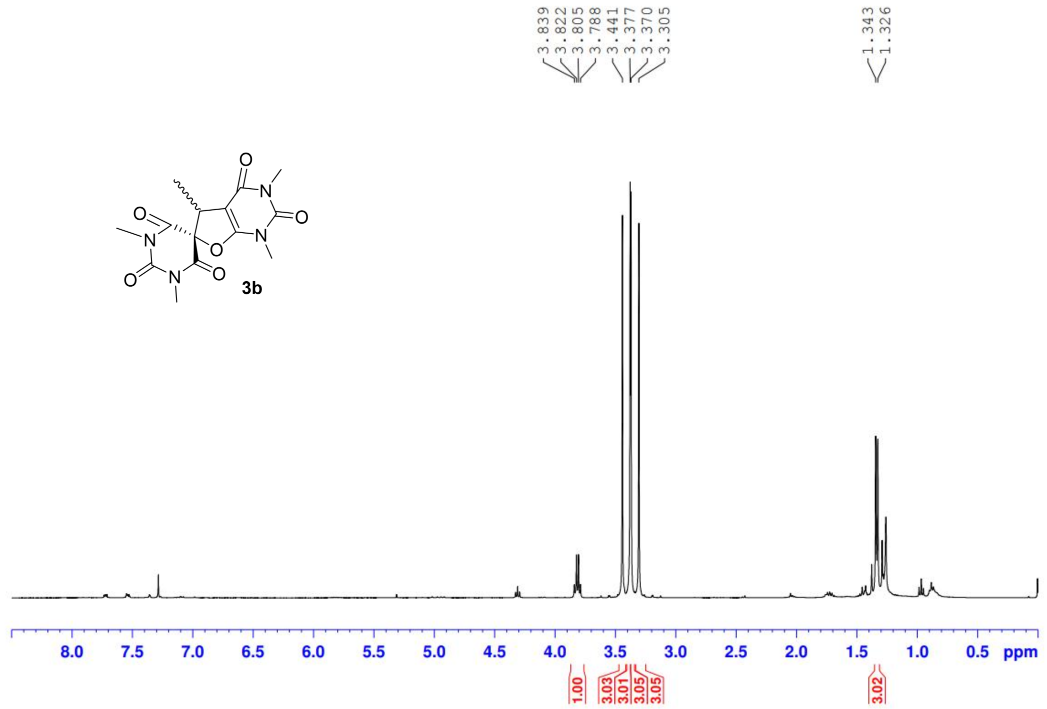

^{13}C NMR (100MHz,$CDCl_3$) of 3b

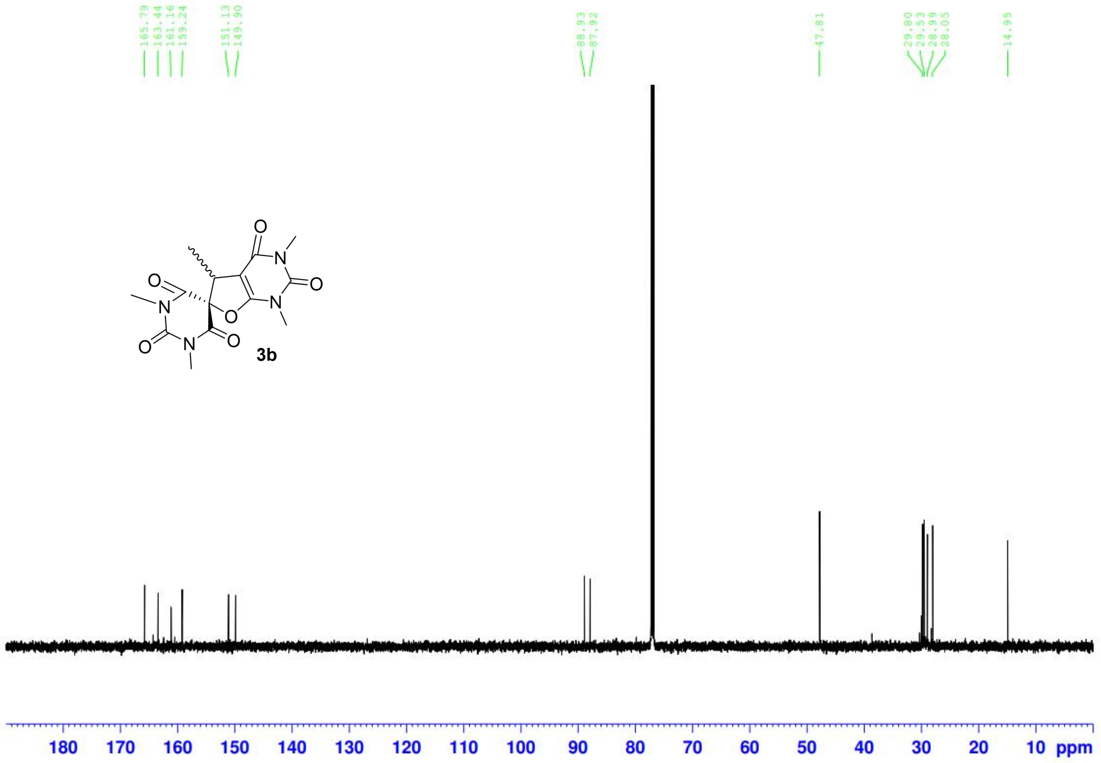

5-(2,3-dihydrobenzofuran-5-yl)-1,1',3,3'-tetramethyl-1,5-dihydro-2H,2'H-spiro[furo[2,3-d]pyrimidine-6,5'-pyrimidine]-2,2',4,4',6'(1'H,3H,3'H)-pentaone(3c):

^{1}H NMR (400MHz,$CDCl_3$) of 3c

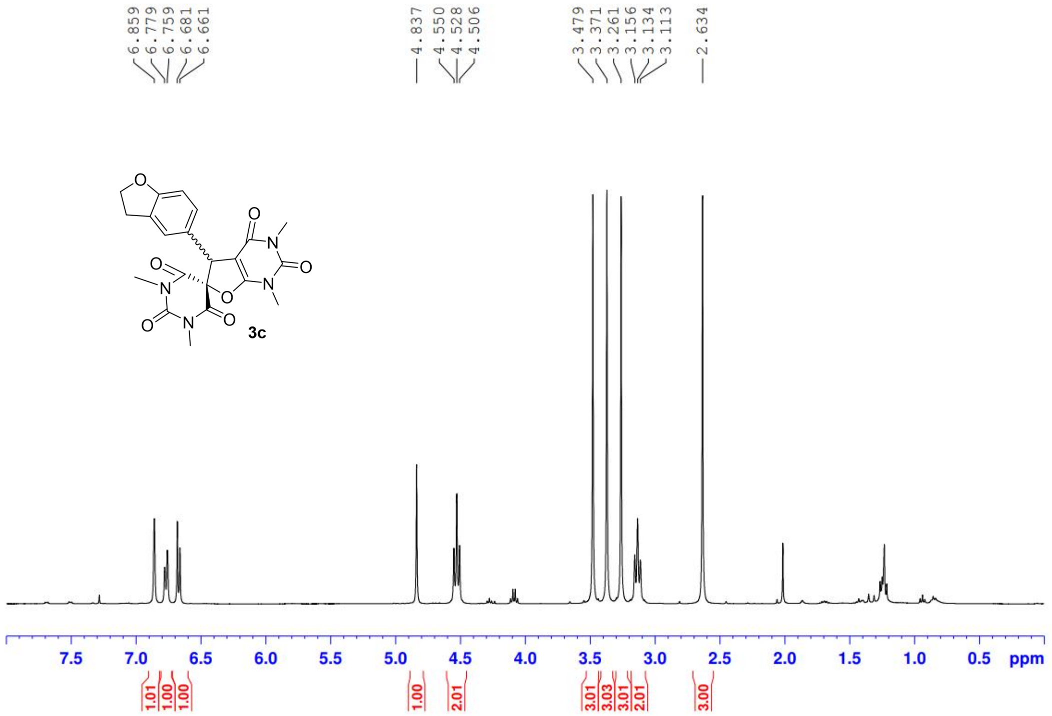

^{13}C NMR (100MHz,$CDCl_3$) of 3c

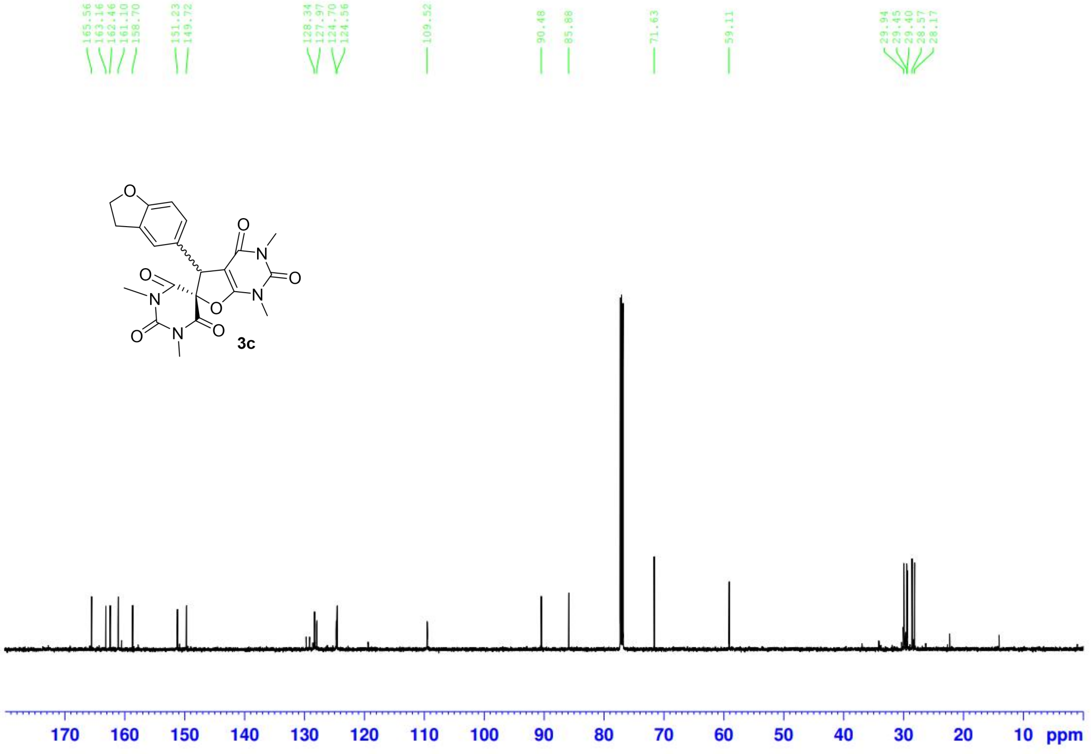

1,1',3,3'-tetrabenzyl-5-phenyl-1,5-dihydro-2H,2'H-spiro[furo[2,3-d]pyrimidine-6,5'-pyrimidine]-2,2',4,4',6'(1'H,3H,3'H)-pentaone (3d):

^{1}H NMR (400MHz,CDCl$_3$) of 3d

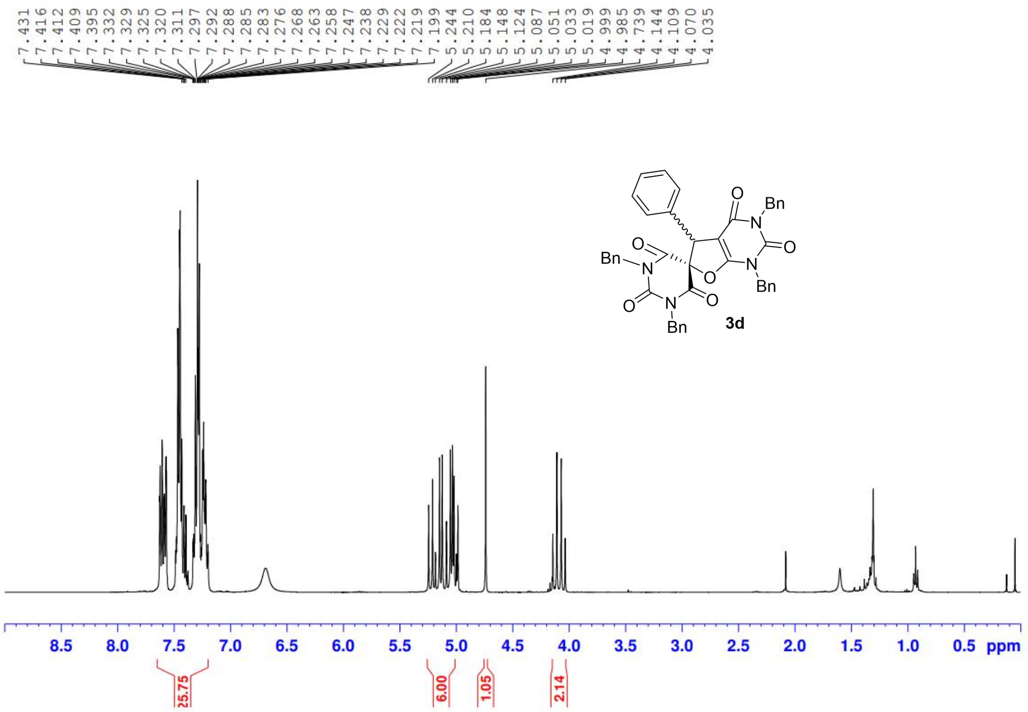

^{13}C NMR (100MHz,CDCl$_3$) of 3d

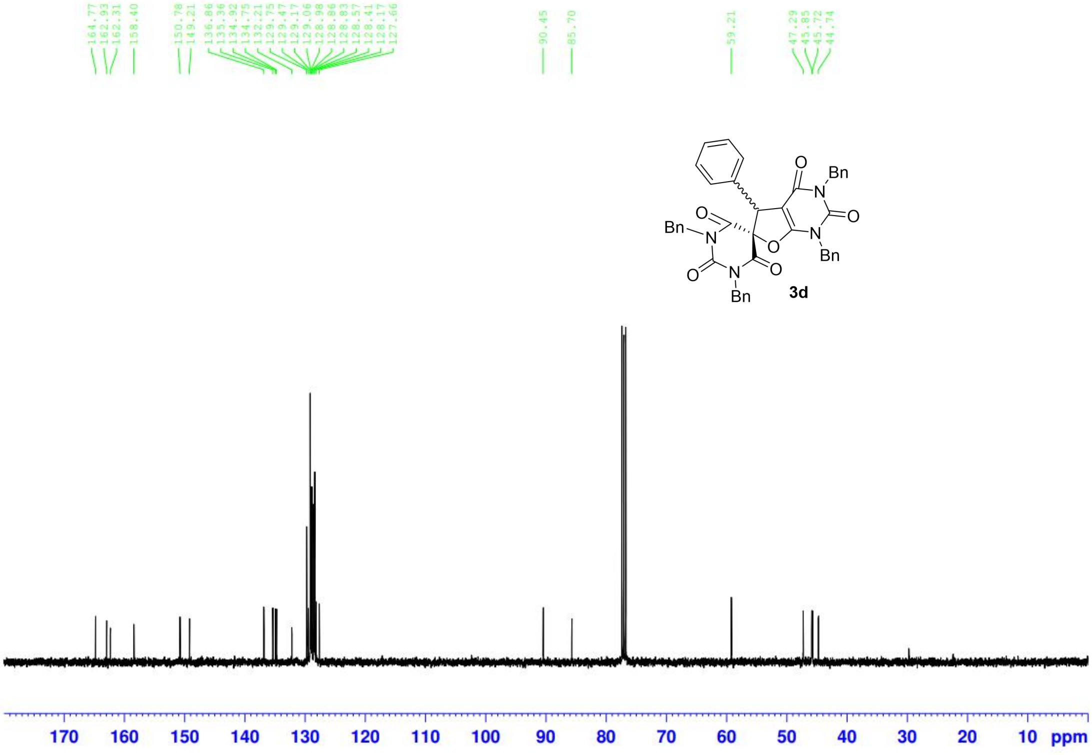

1,1',3,3'-tetraethyl-5-phenyl-1,5-dihydro-2H,2'H-spiro[furo[2,3-d]pyrimidine-6,5'-pyrimidine]-2,2',4,4',6'(1'H,3H,3'H)-pentaone (3e):

^{1}H NMR (400MHz,CDCl₃) of 3e

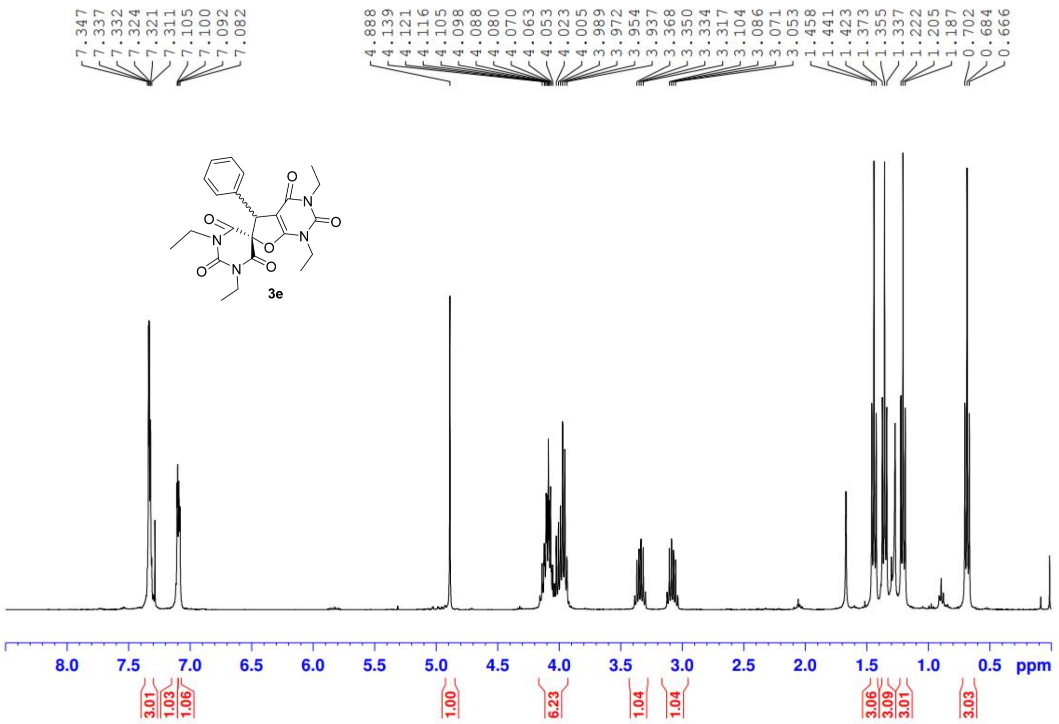

^{13}C NMR (100MHz,CDCl₃) of 3e

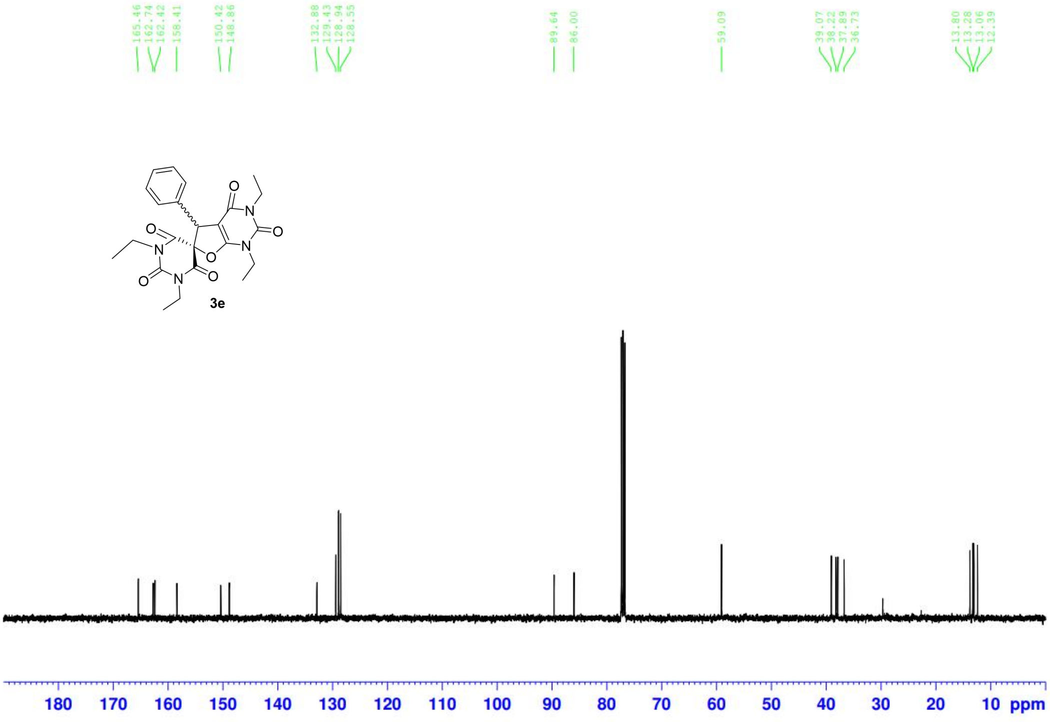

1,1',3,3'-tetraisopropyl-5-phenyl-1,5-dihydro-2H,2'H-spiro[furo[2,3-d]pyrimidine-6,5'-pyrimidine]-2,2',4,4',6'(1'H,3H,3'H)-pentaone(3f):

^{1}H NMR (400MHz,$CDCl_3$) of 3f

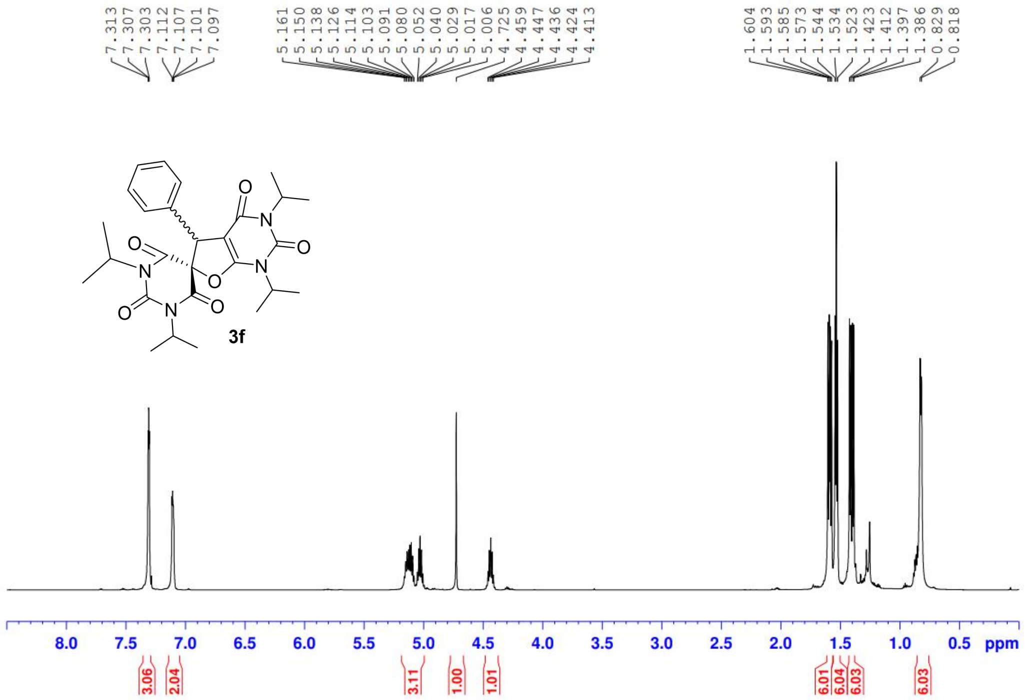

^{13}C NMR (100MHz,$CDCl_3$) of 3f

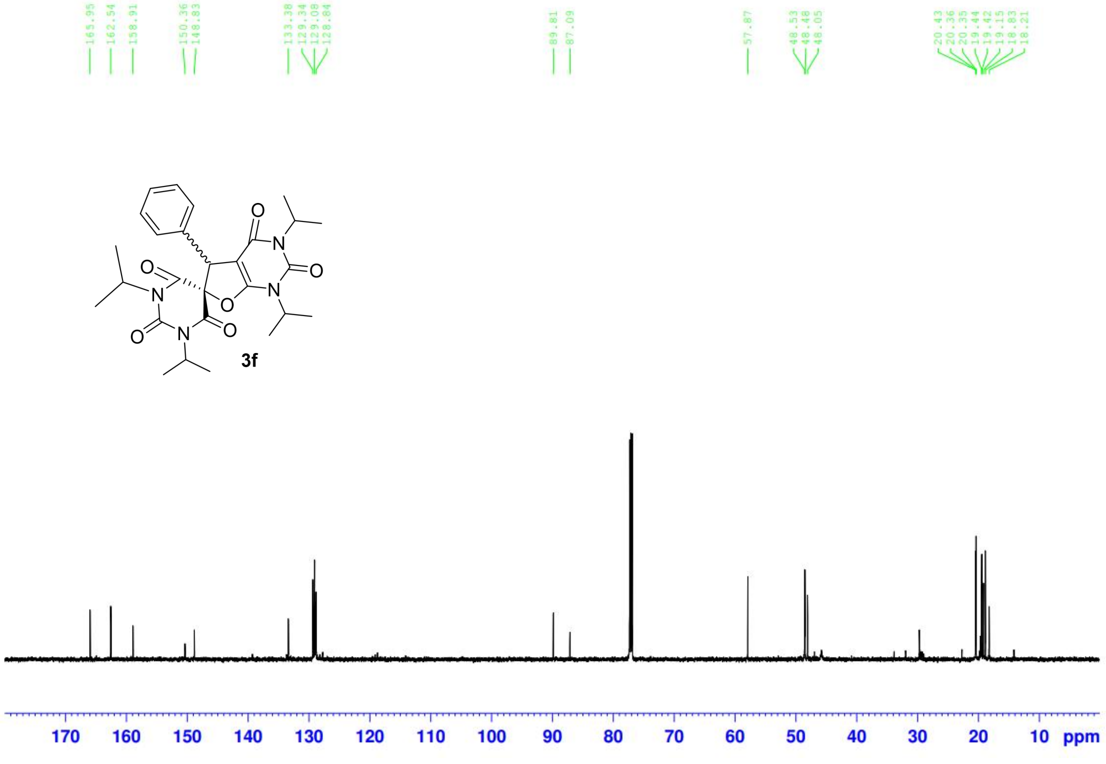

1,1',3,3'-tetramethyl-5-(p-tolyl)-1,5-dihydro-2H,2'H-spiro[furo[2,3-d]pyrimidine-6,5'-pyrimidine]-2,2',4,4',6'(1'H,3H,3'H)-pentaone(3g):

^{1}H NMR (400MHz,CDCl$_3$) of 3g

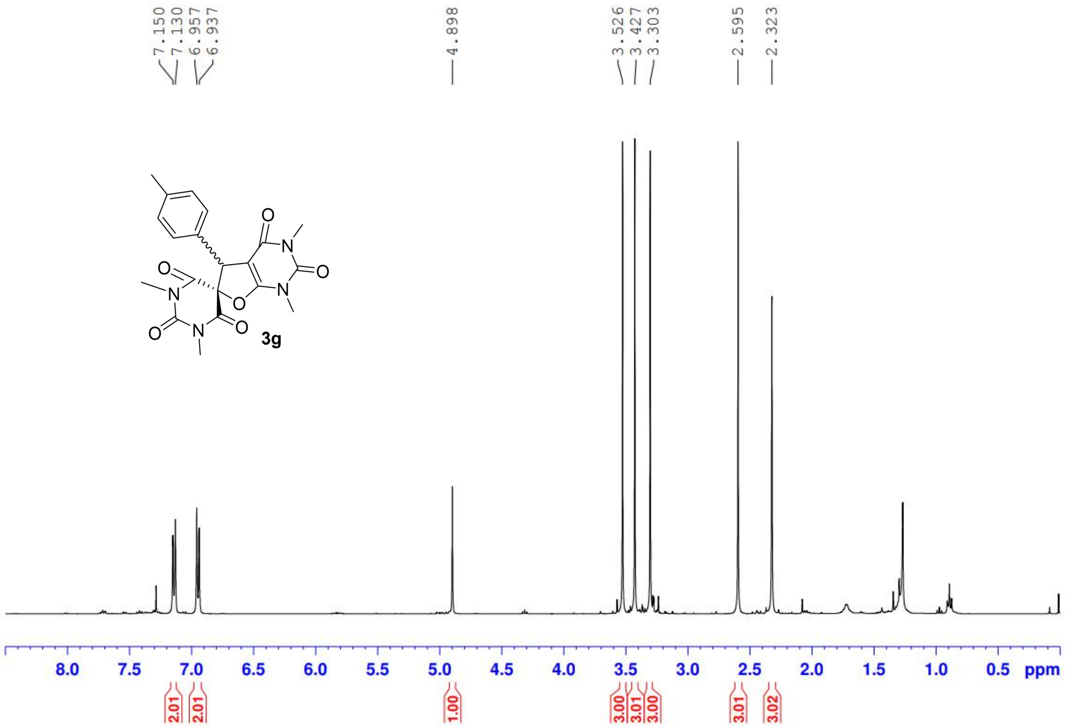

^{13}C NMR (100MHz,CDCl$_3$) of 3g

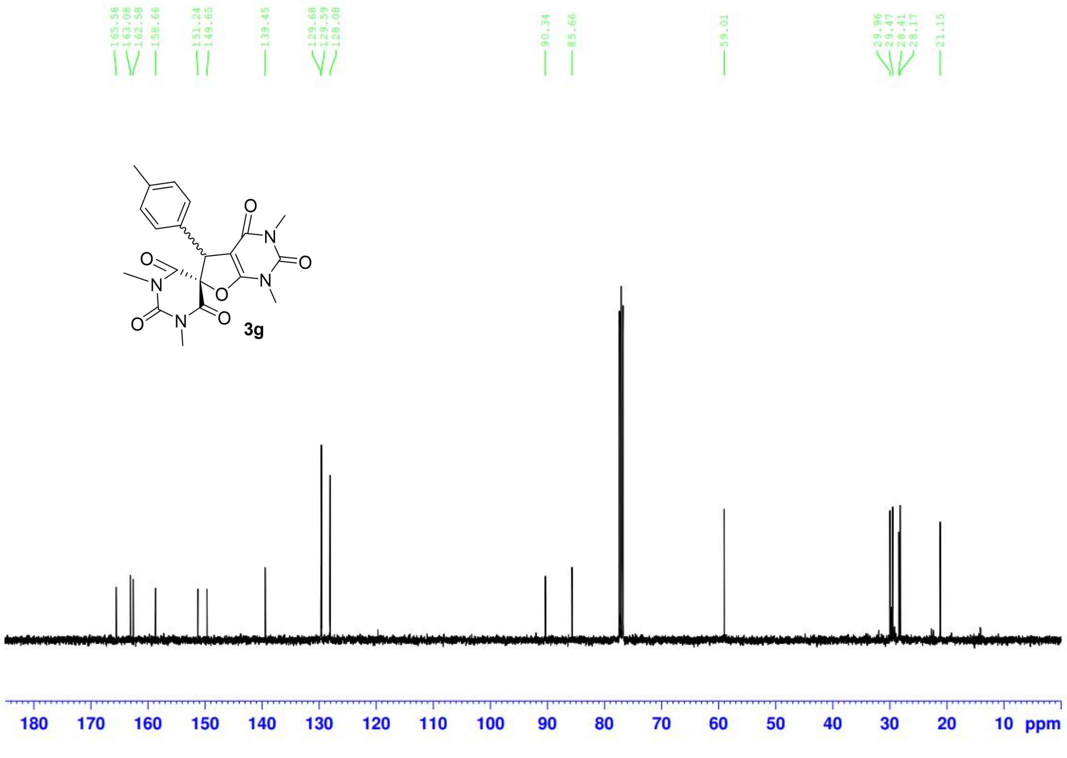

5-(4-bromophenyl)-1,1',3,3'-tetramethyl-1,5-dihydro-2H,2'H-spiro[furo[2,3-d]pyrimidine-6,5'-pyrimidine]-2,2',4,4',6'(1'H,3H,3'H)-pentaone(3h):

^{1}H NMR (400MHz,$CDCl_3$) of 3h

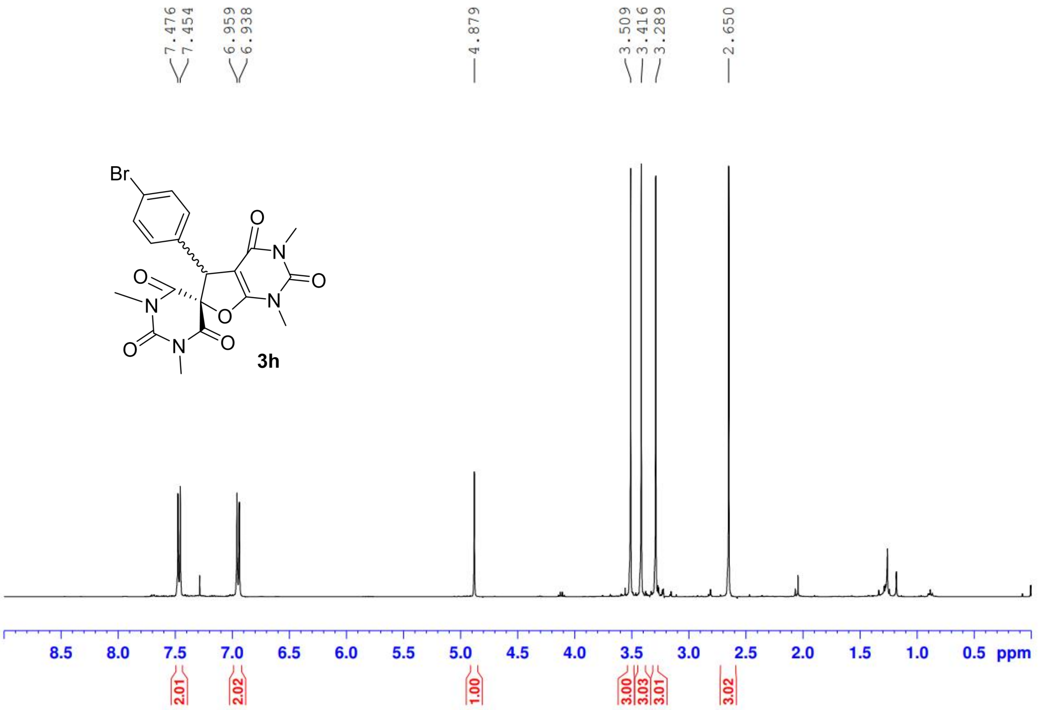

^{13}C NMR (100MHz,$CDCl_3$) of 3h

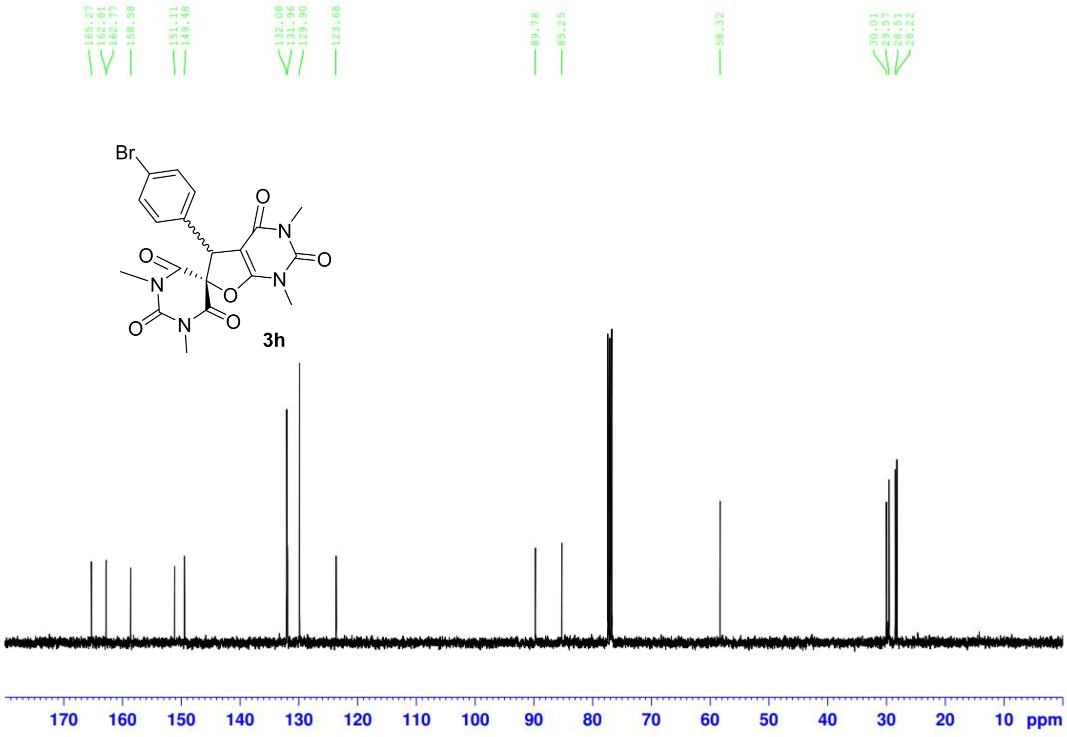

5-(3-chlorophenyl)-1,1',3,3'-tetramethyl-1,5-dihydro-2H,2'H-spiro[furo[2,3-d]pyrimidine-6,5'-pyrimidine]-2,2',4,4',6'(1'H,3H,3'H)-pentaone (3i):

^{1}H NMR (400MHz,CDCl$_3$) of 3i

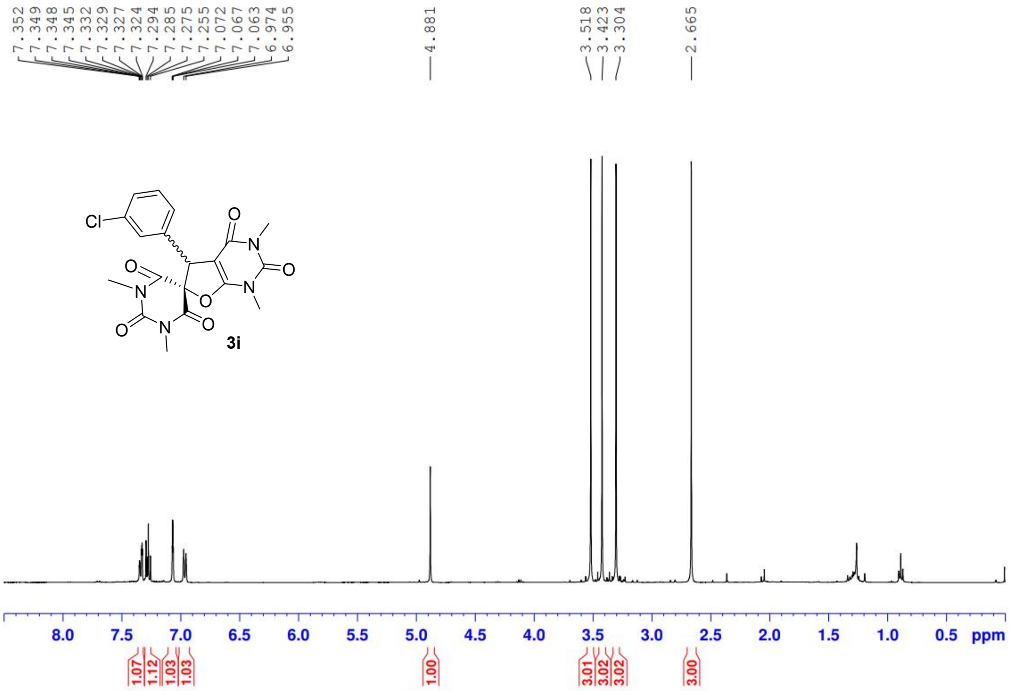

^{13}C NMR (100MHz,CDCl$_3$) of 3i

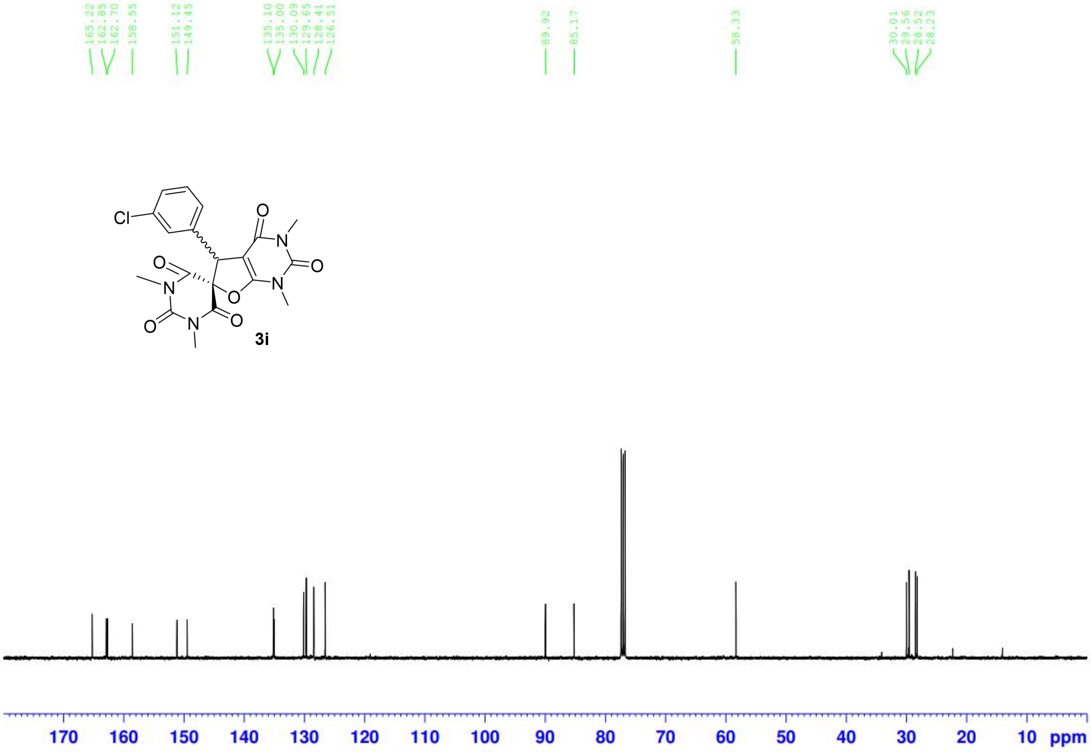

1,1',3,3'-tetramethyl-5-(4-(naphthalen-2-yl)phenyl)-1,5-dihydro-2H,2'H-spiro[furo[2,3-d]pyrimidine-6,5'-pyrimidine]-2,2',4,4',6'(1'H,3H,3'H)-pentaone(3j):

^{1}H NMR (400MHz,$CDCl_3$) of 3j

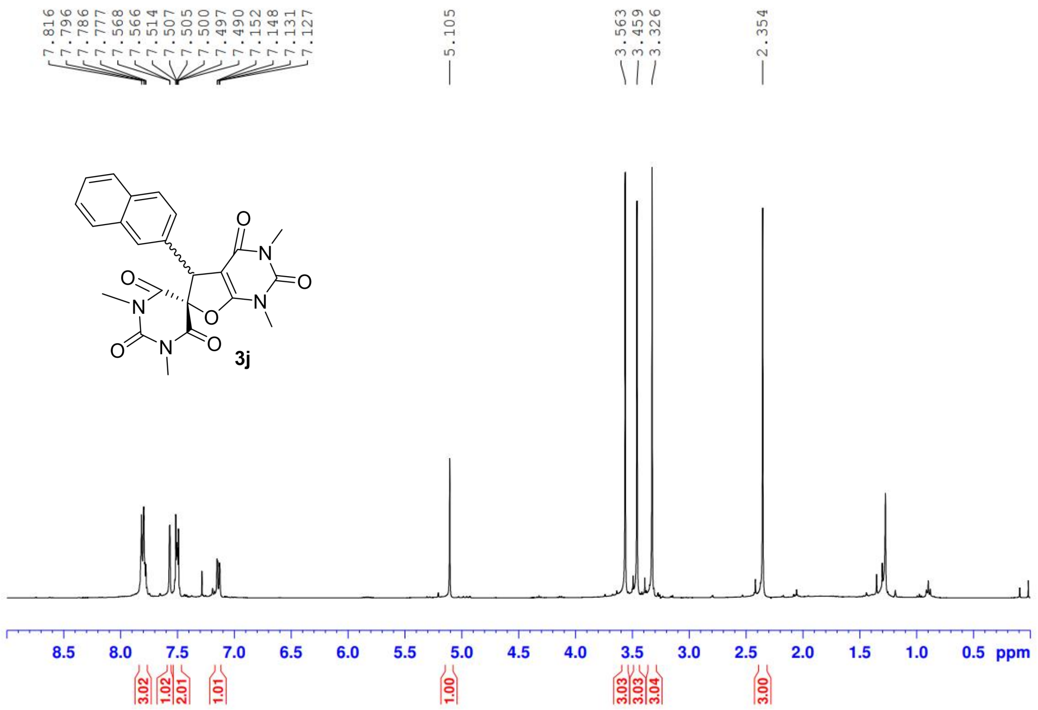

^{13}C NMR (100MHz,$CDCl_3$) of 3j

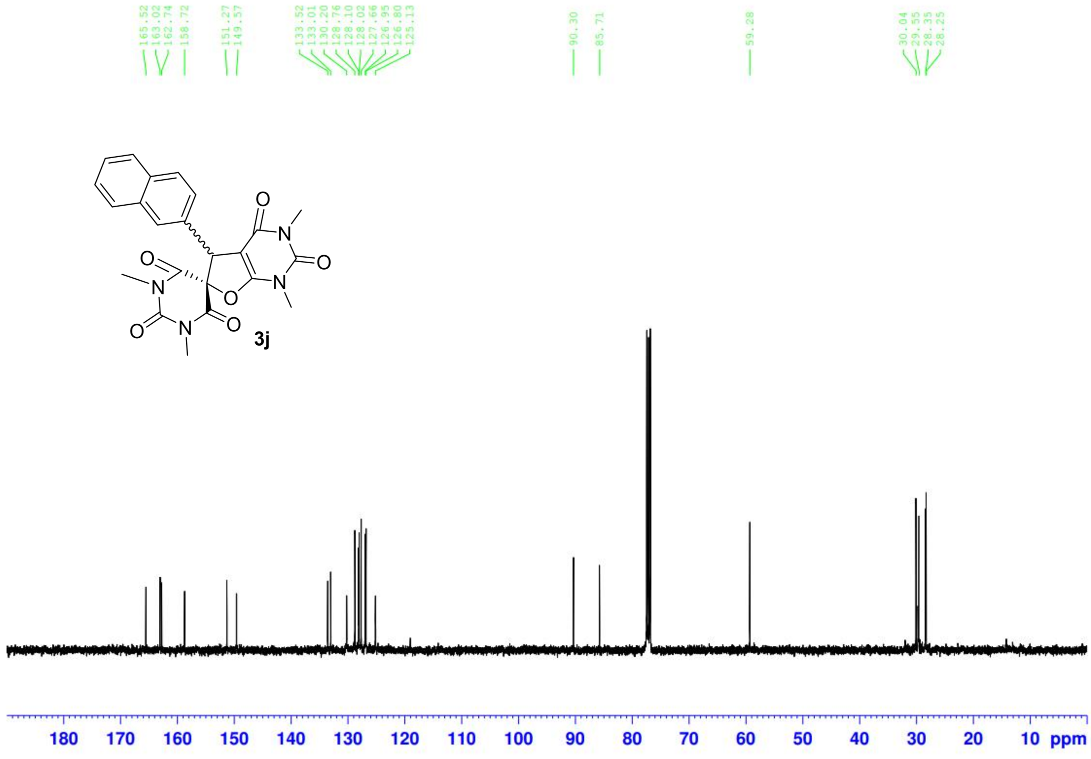

1,1',3,3'-tetramethyl-5-(4-(trifluoromethyl)phenyl)-1,5-dihydro-2H,2'H-spiro[furo[2,3-d]pyrimidine-6,5'-pyrimidine]-2,2',4,4',6'(1'H,3H,3'H)-pentaone(3k):

^{1}H NMR (400MHz,$CDCl_3$) of 3k

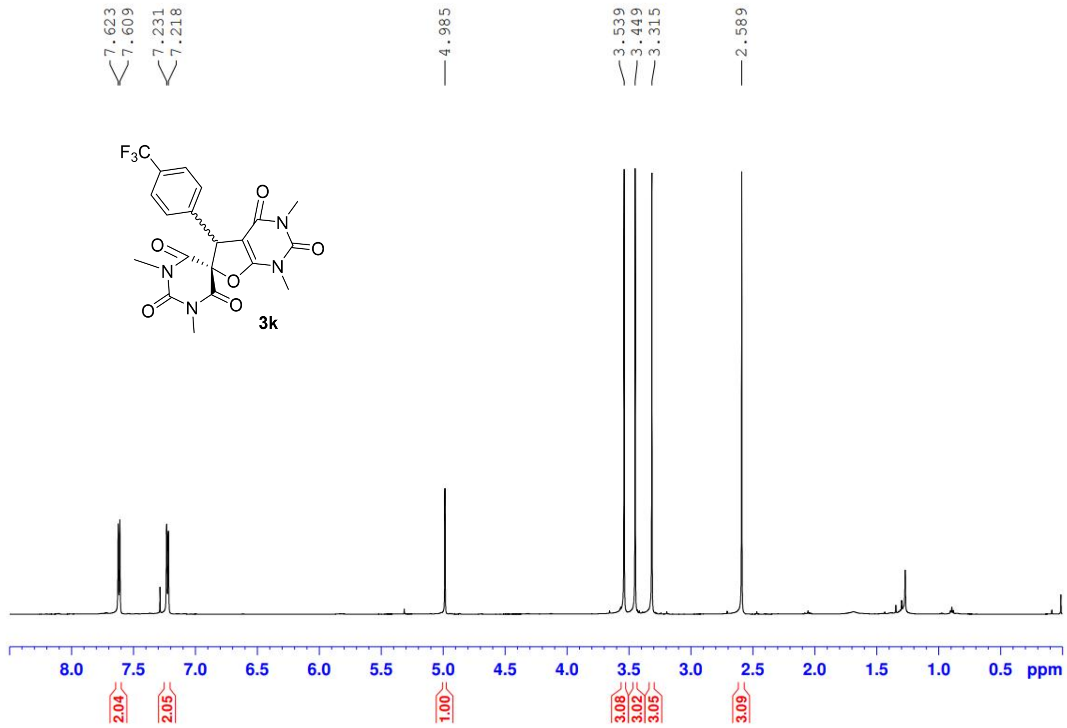

^{13}C NMR (100MHz,$CDCl_3$) of 3k

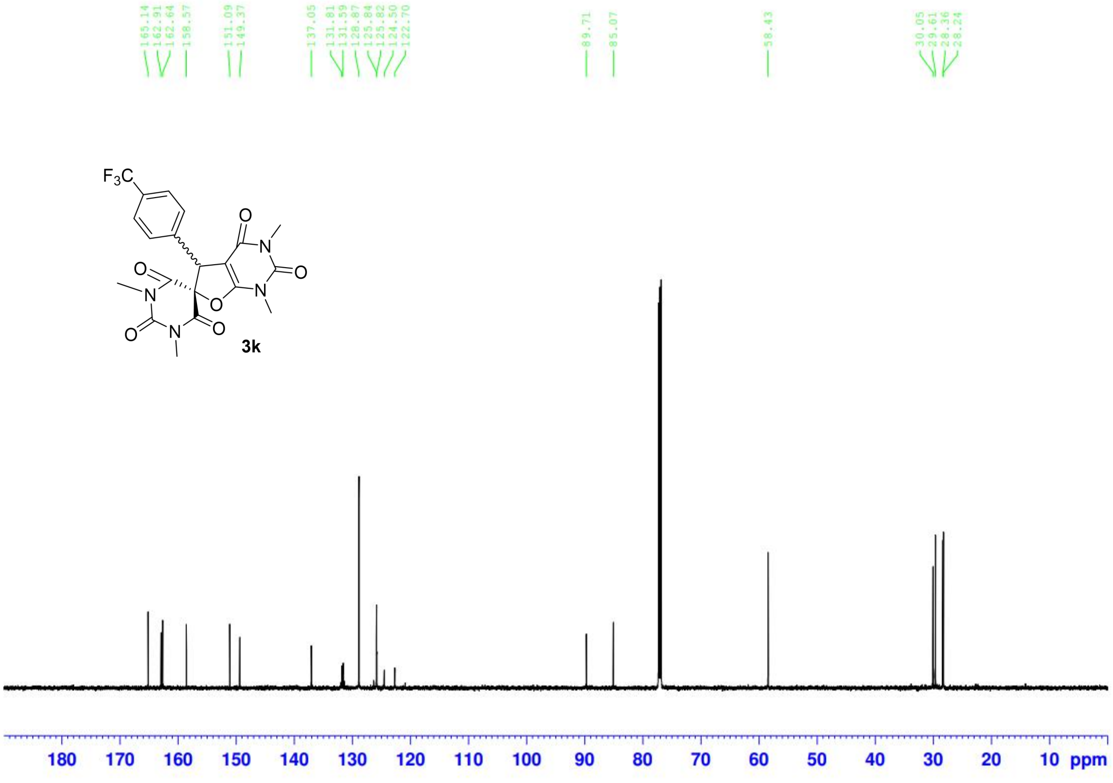

^{19}F NMR (376MHz,CDCl$_3$) of 3k

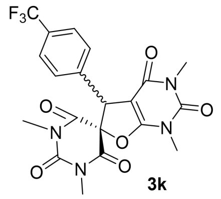

3k

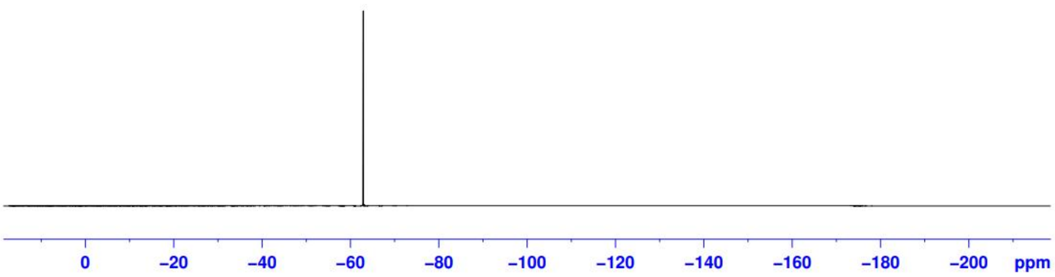

5-(4-methoxyphenyl)-1,1',3,3'-tetramethyl-1,5-dihydro-2H,2'H-spiro[furo[2,3-d]pyrimidine-6,5'-pyrimidine]-2,2',4,4',6'(1'H,3H,3'H)-pentaone(3l):

^{1}H NMR (400MHz,$CDCl_3$) of 3l

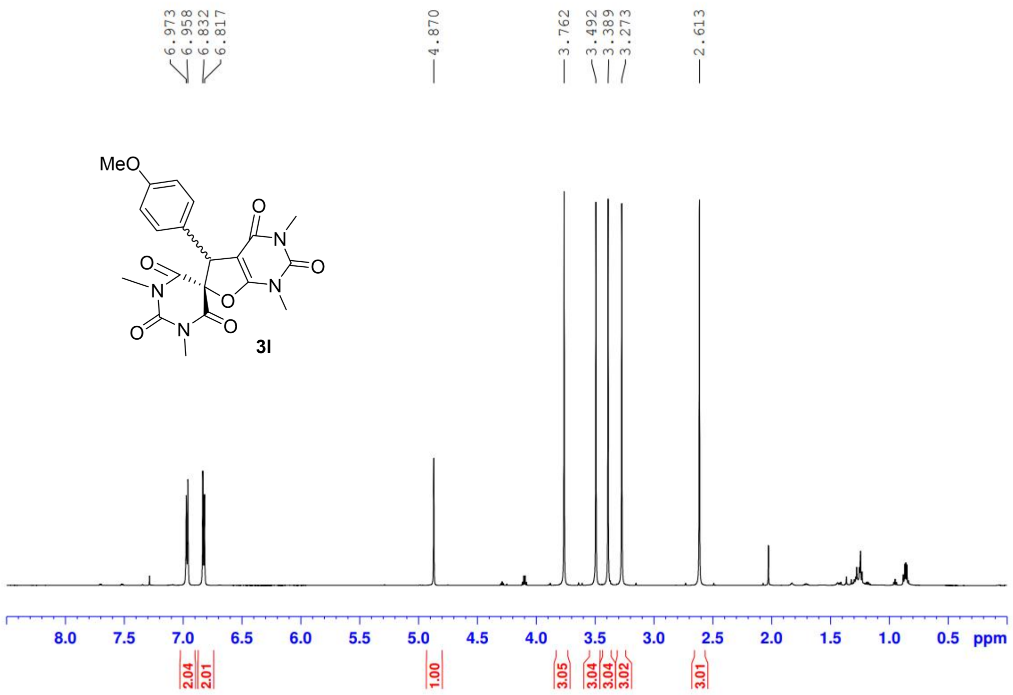

^{13}C NMR (100MHz,$CDCl_3$) of 3l

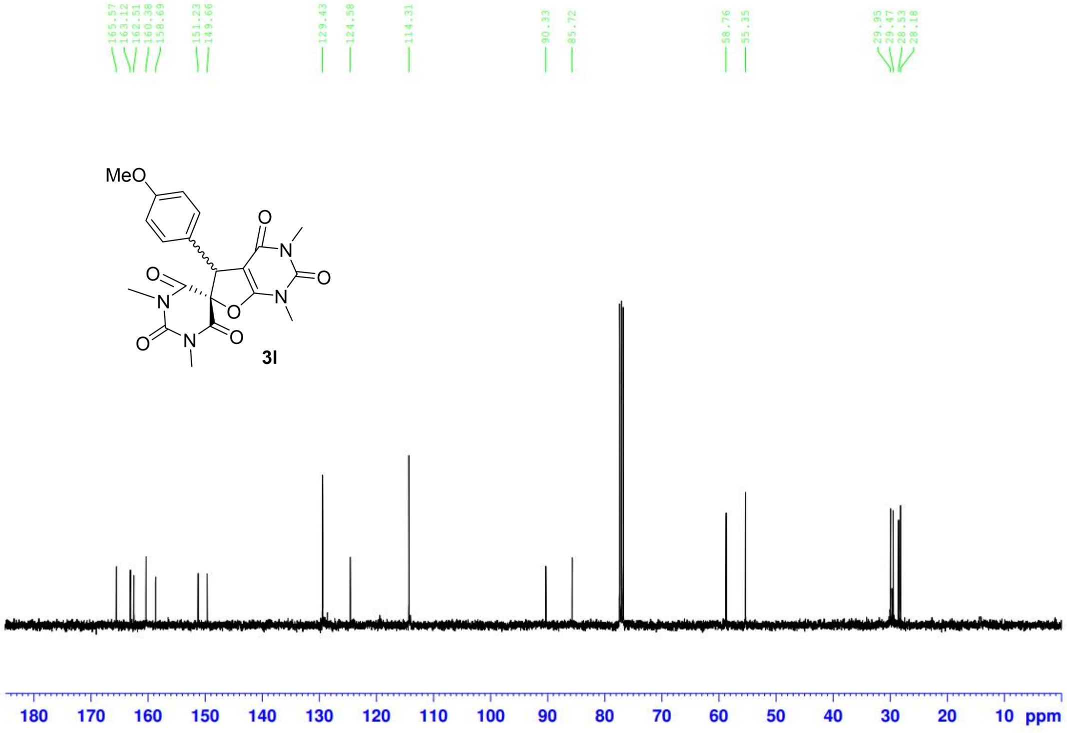

1,1',3,3'-tetramethyl-5-(4-nitrophenyl)-1,5-dihydro-2H,2'H-spiro[furo[2,3-d]pyrimidine-6,5'-pyrimidine]-2,2',4,4',6'(1'H,3H,3'H)-pentaone(3m):

^{1}H NMR (400MHz,DMSO) of 3m

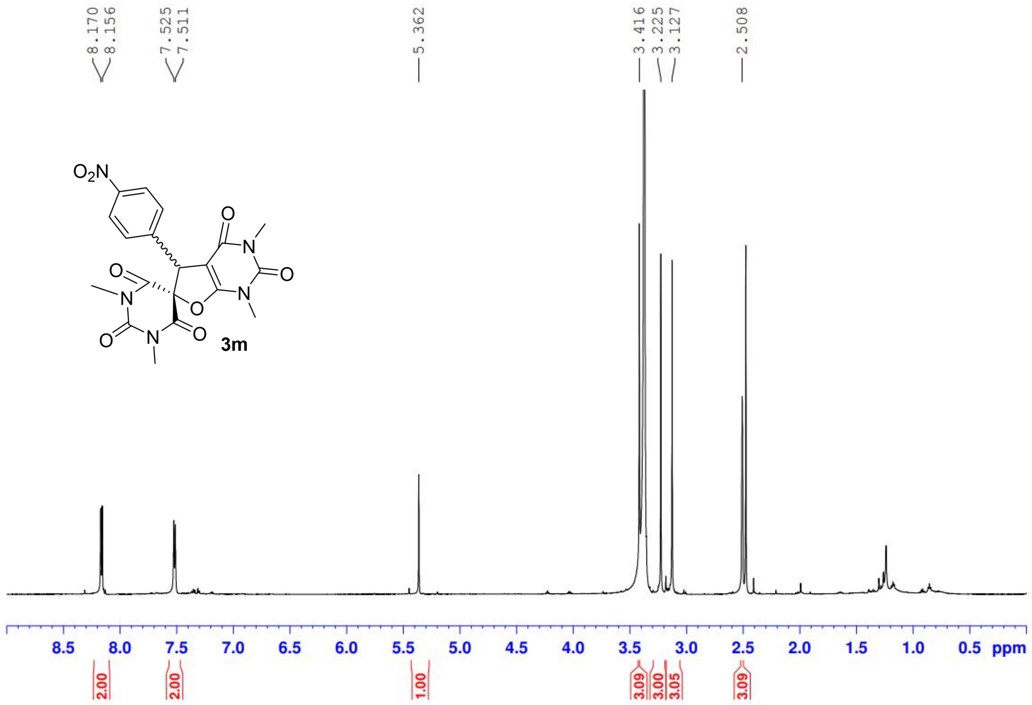

^{13}C NMR (100MHz,DMSO) of 3m

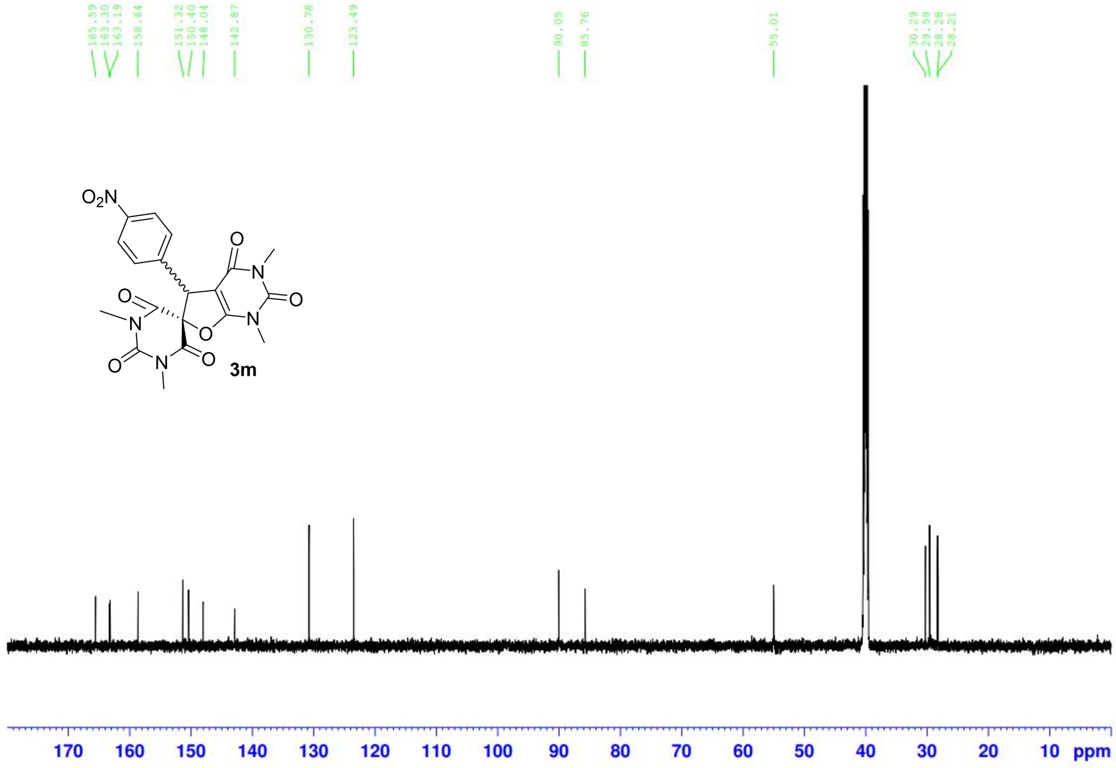

5-(2-bromophenyl)-1,1',3,3'-tetramethyl-1,5-dihydro-2H,2'H-spiro[furo[2,3-d]pyrimidine-6,5'-pyrimidine]-2,2',4,4',6'(1'H,3H,3'H)-pentaone(3n):

^{1}H NMR (400MHz,CDCl$_3$) of 3n

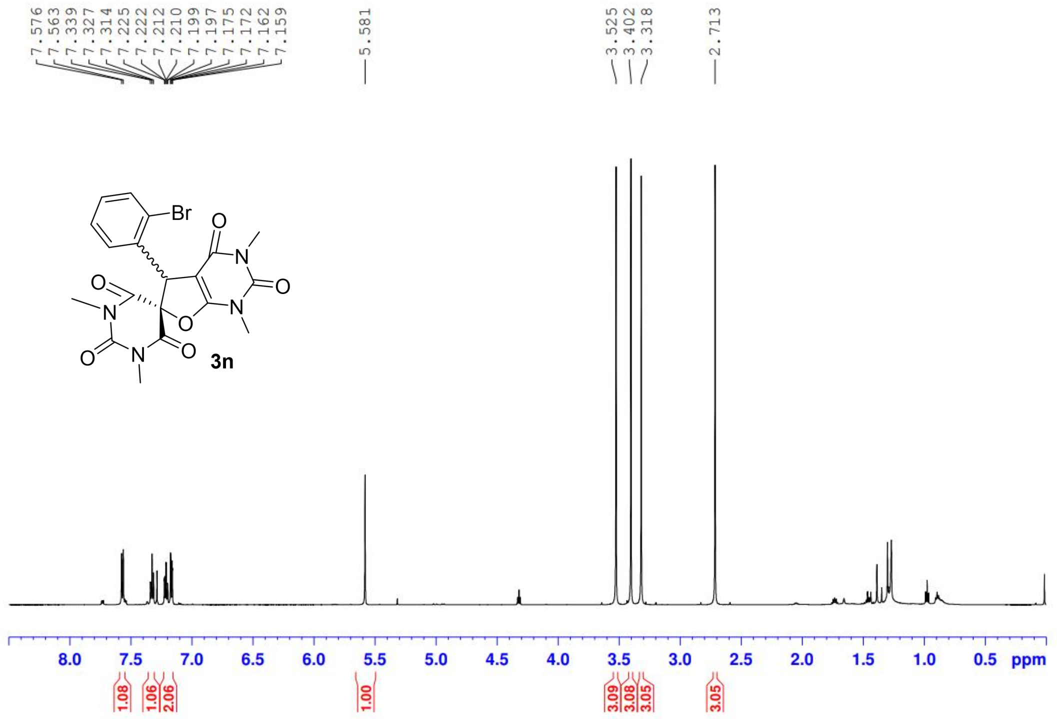

^{13}C NMR (100MHz,CDCl$_3$) of 3n

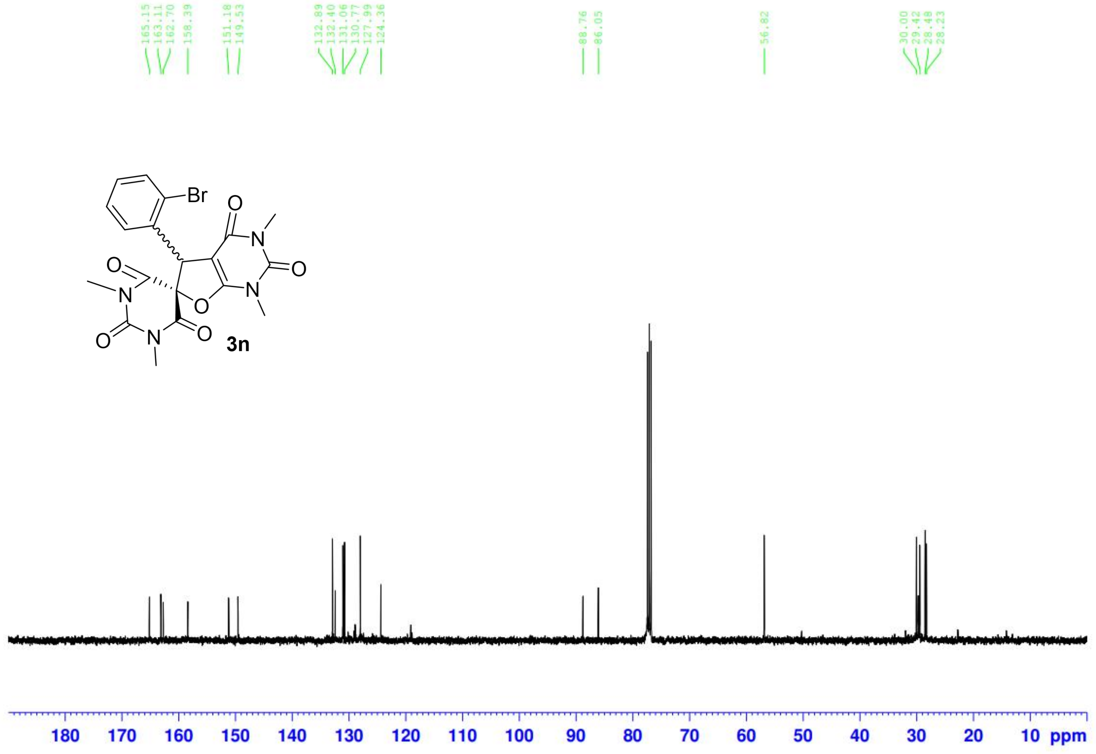

1,1',3,3'-tetramethyl-5-(m-tolyl)-1,5-dihydro-2H,2'H-spiro[furo[2,3-d]pyrimidine -6,5'-pyrimidine]-2,2',4,4',6'(1'H,3H,3'H)-pentaone (3o):

^{1}H NMR (400MHz,CDCl$_3$) of 3o

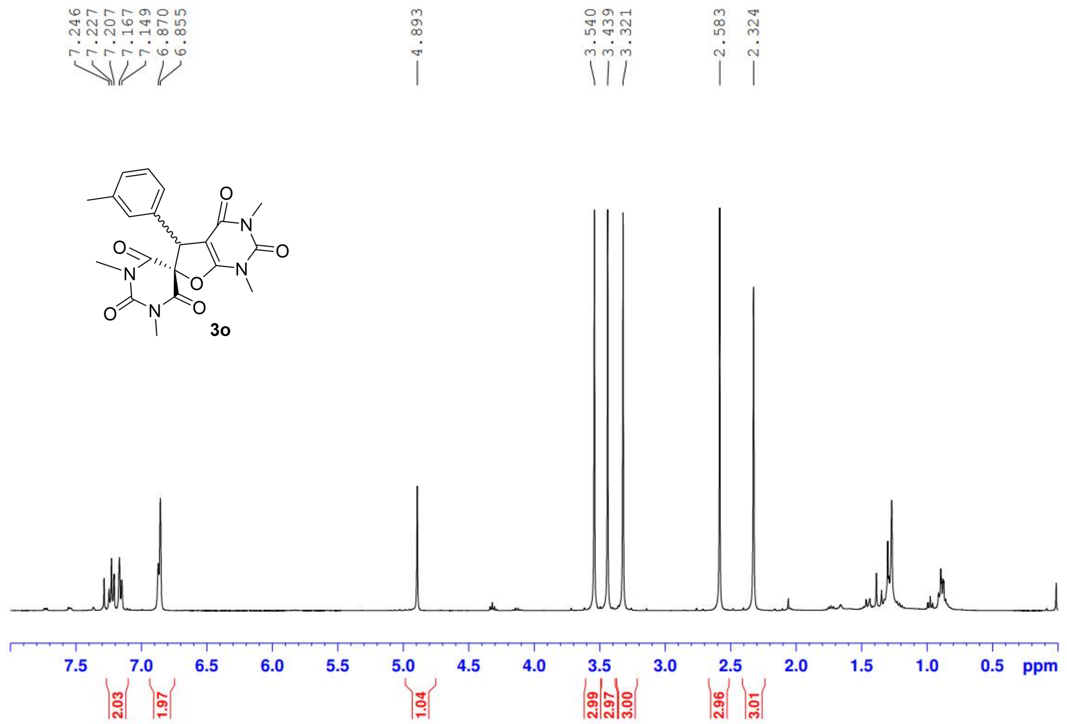

^{13}C NMR (100MHz,CDCl$_3$) of 3o

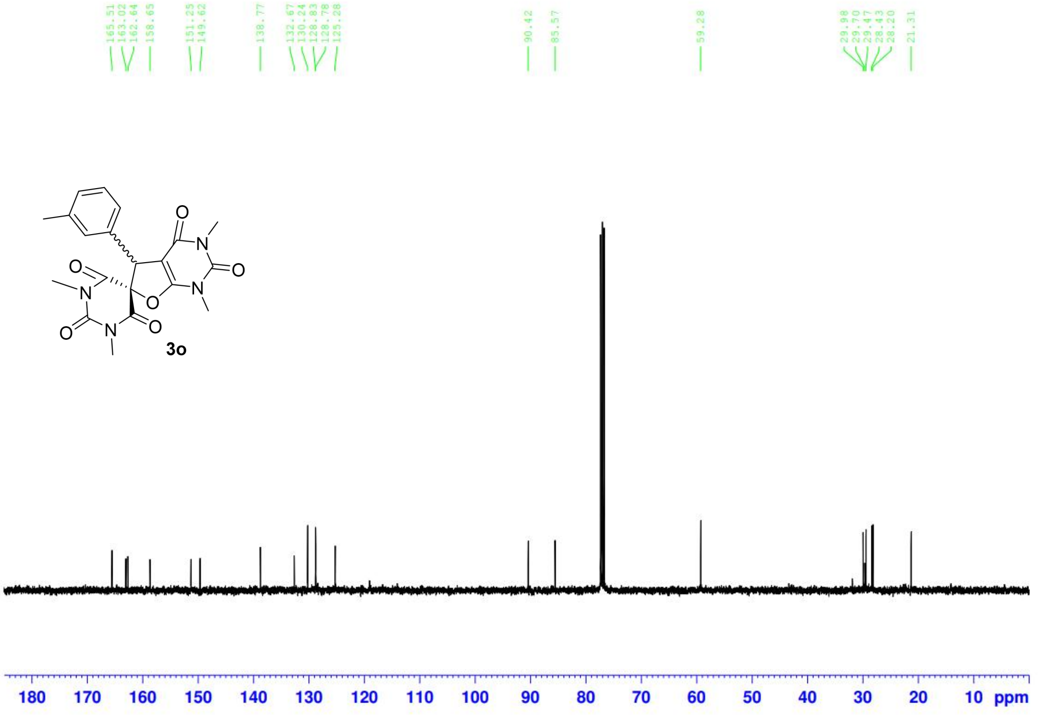

1,1',3,3'-tetraethyl-5-(p-tolyl)-1,5-dihydro-2H,2'H-spiro[furo[2,3-d]pyrimidine-6,5'-pyrimidine]-2,2',4,4',6'(1'H,3H,3'H)-pentaone (3p):

^{1}H NMR (400MHz,CDCl$_3$) of 3p

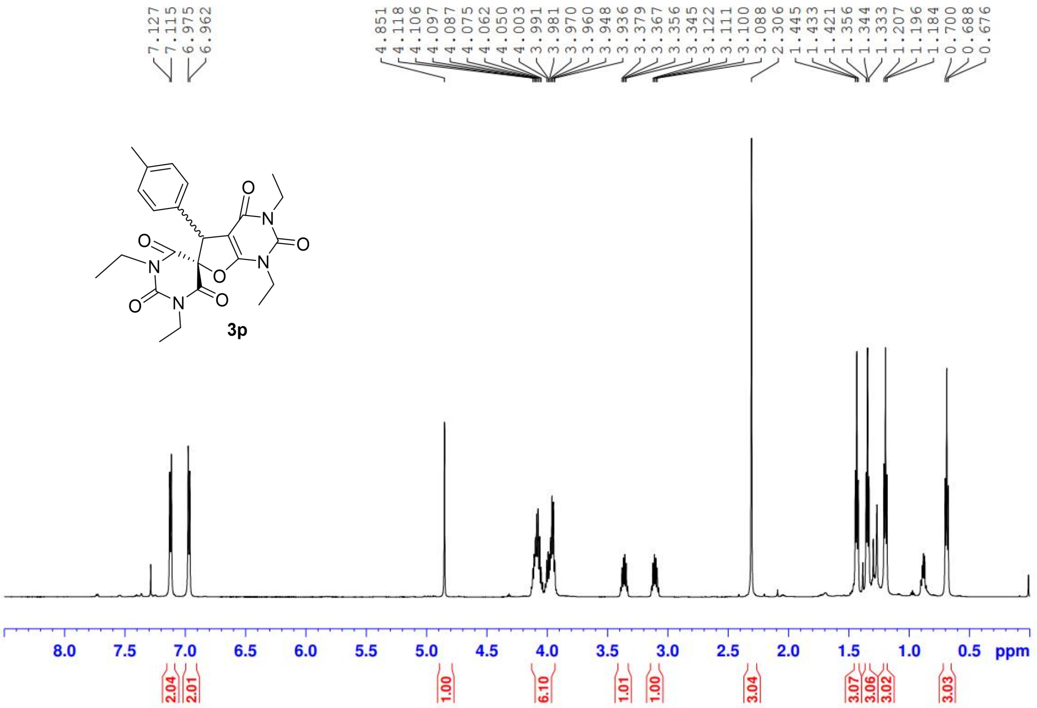

^{13}C NMR (100MHz,CDCl$_3$) of 3p

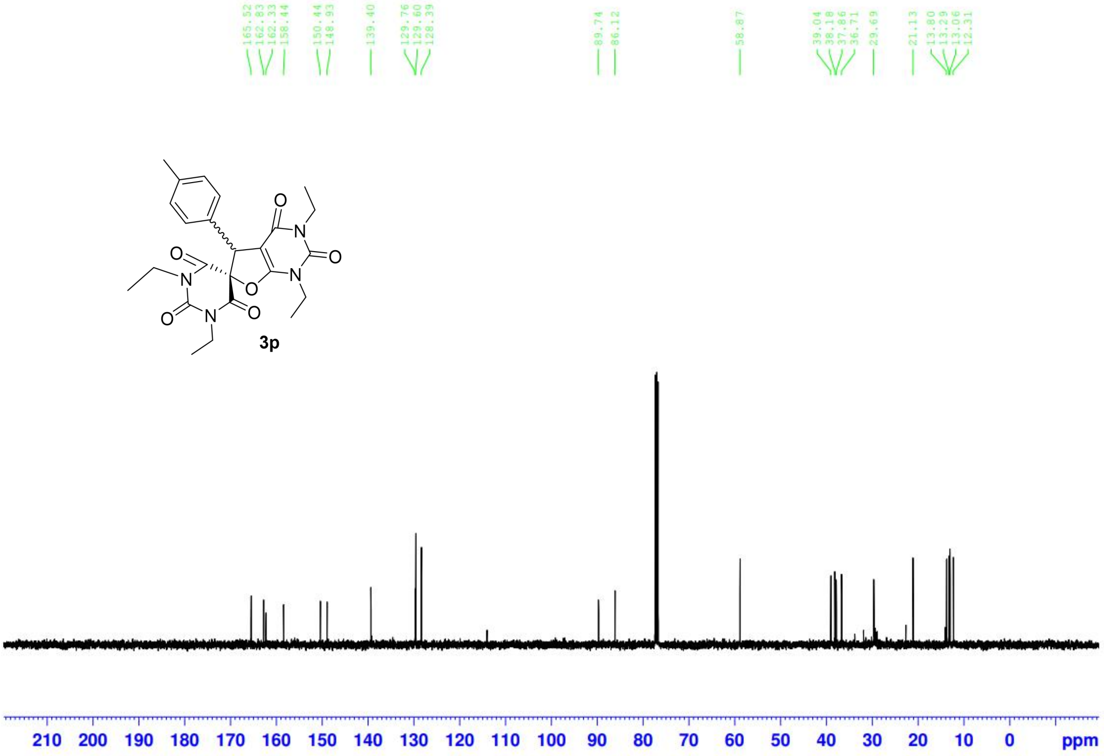

5-(4-bromophenyl)-1,1',3,3'-tetraethyl-1,5-dihydro-2H,2'H-spiro[furo[2,3-d]pyrimidine-6,5'-pyrimidine]-2,2',4,4',6'(1'H,3H,3'H)-pentaone(3q):

^{1}H NMR (400MHz,$CDCl_3$) of 3q

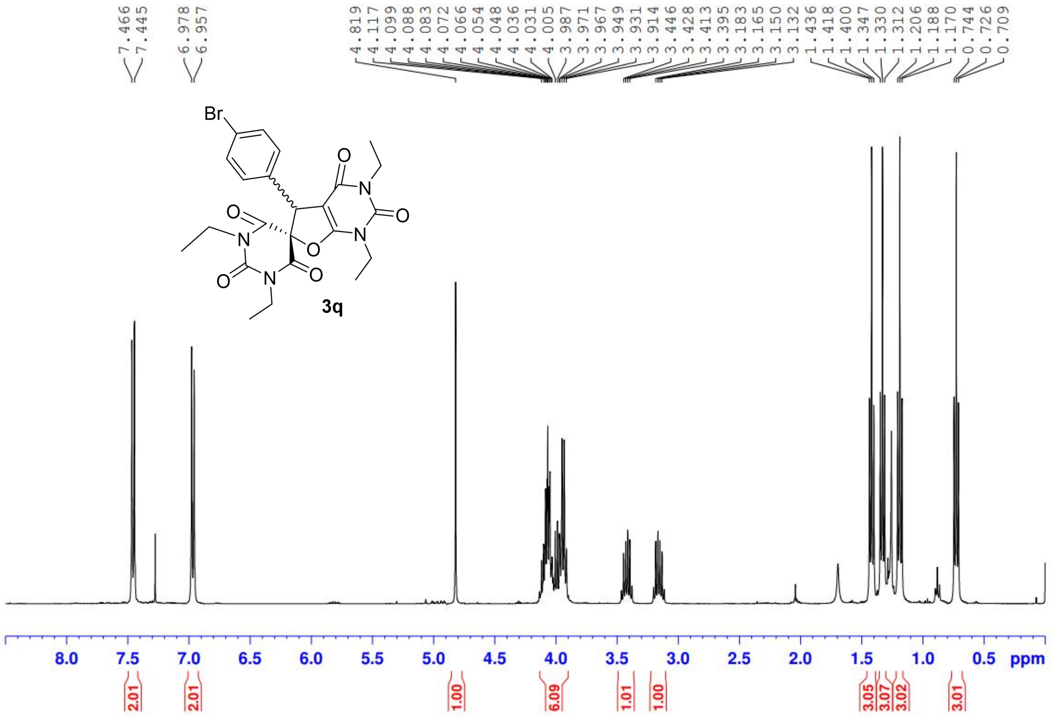

^{13}C NMR (100MHz,$CDCl_3$) of 3q

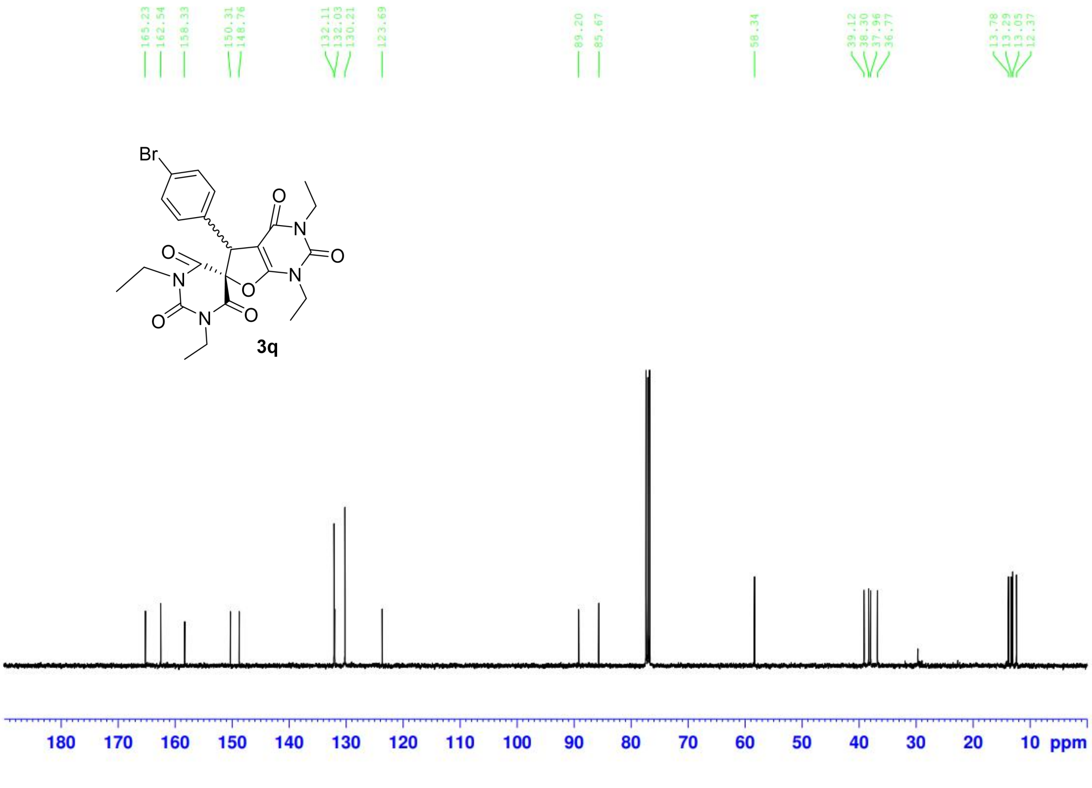

Dept-135 of 3q

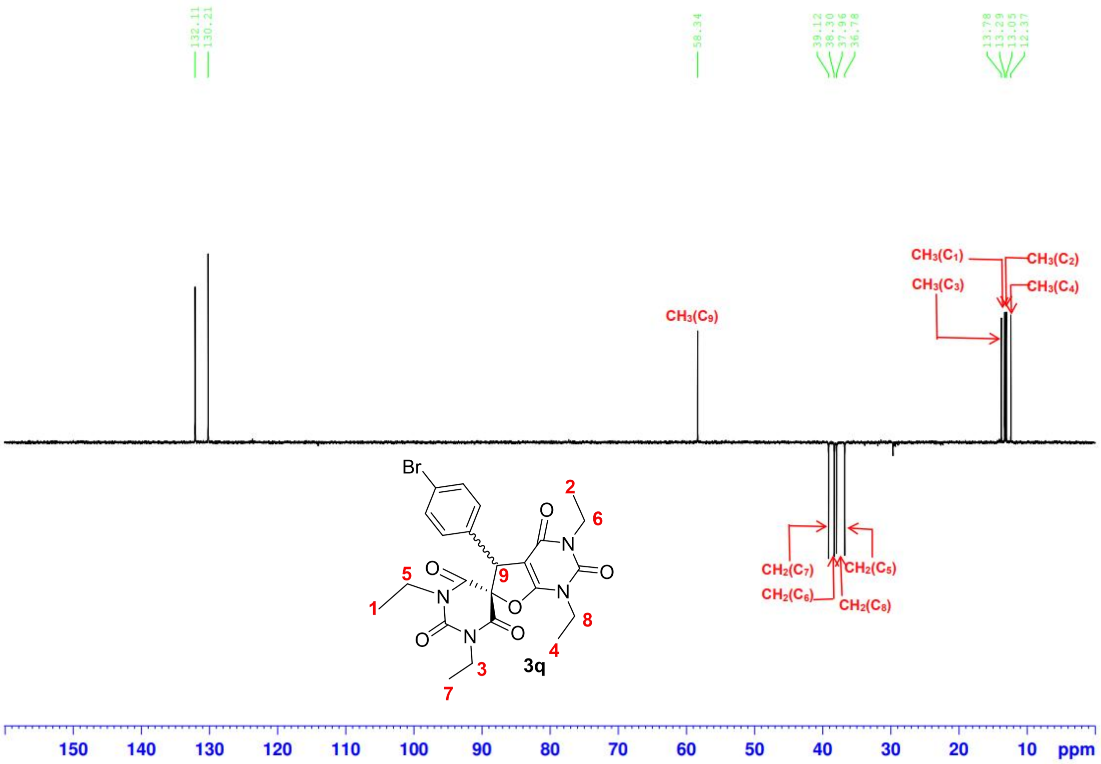

HSQC of 3q

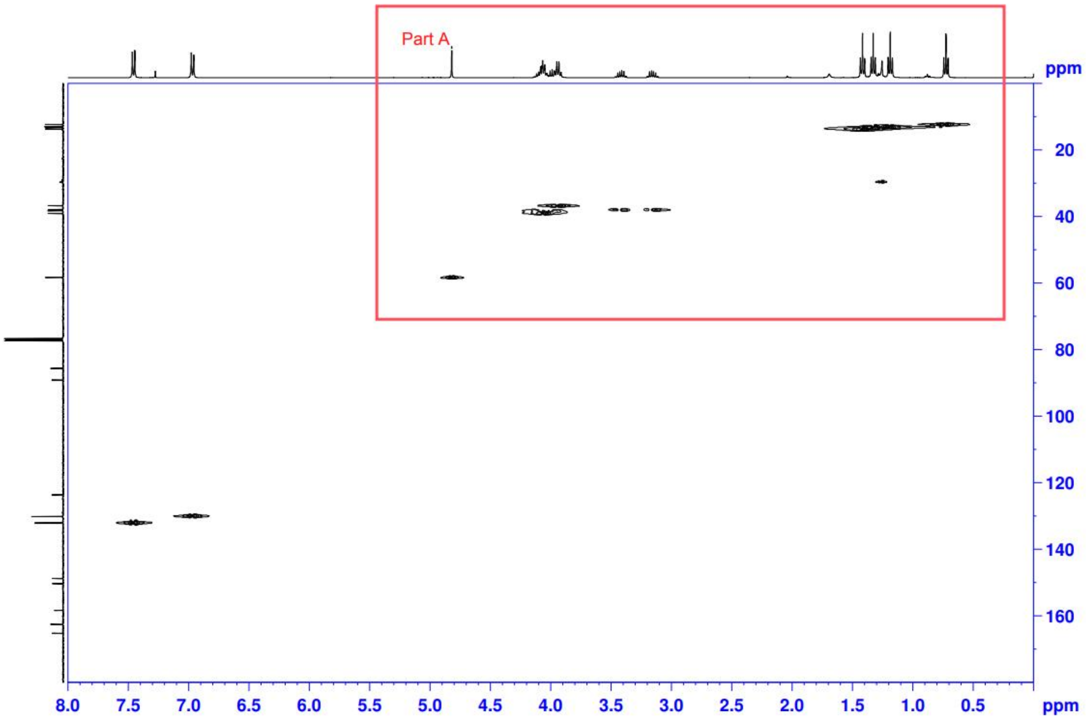

HSQC of Part A

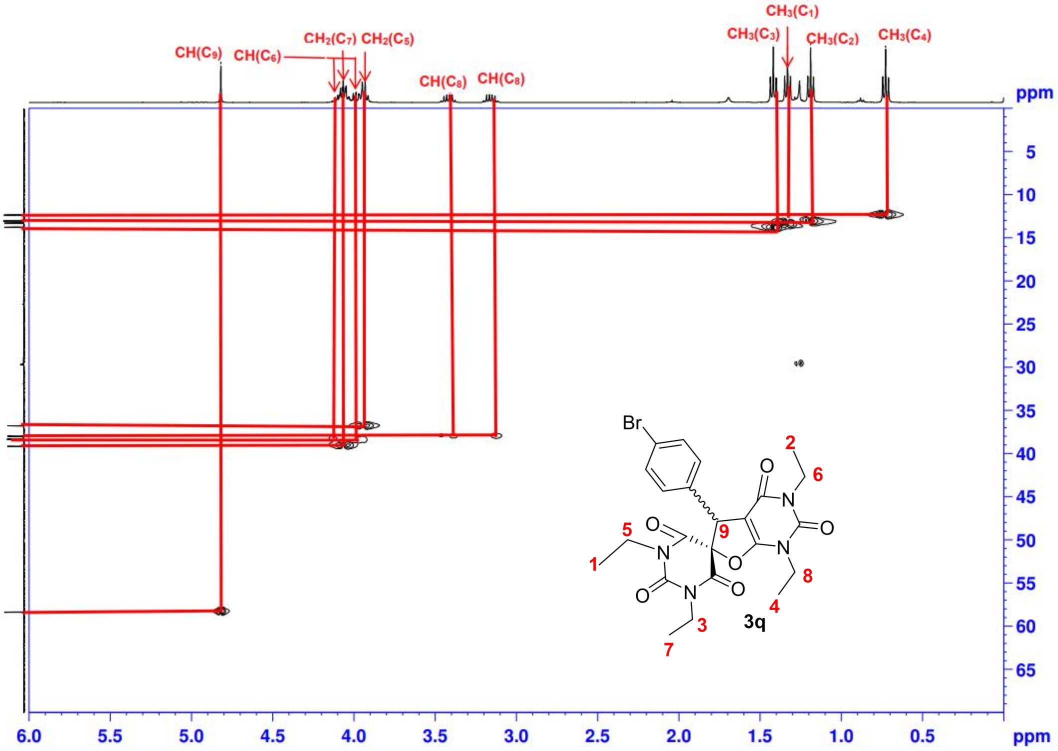

5-(3-chlorophenyl)-1,1',3,3'-tetraethyl-1,5-dihydro-2H,2'H-spiro[furo[2,3-d]pyrimidine-6,5'-pyrimidine]-2,2',4,4',6'(1'H,3H,3'H)-pentaone(3r):

^{1}H NMR (400MHz,$CDCl_3$) of 3r

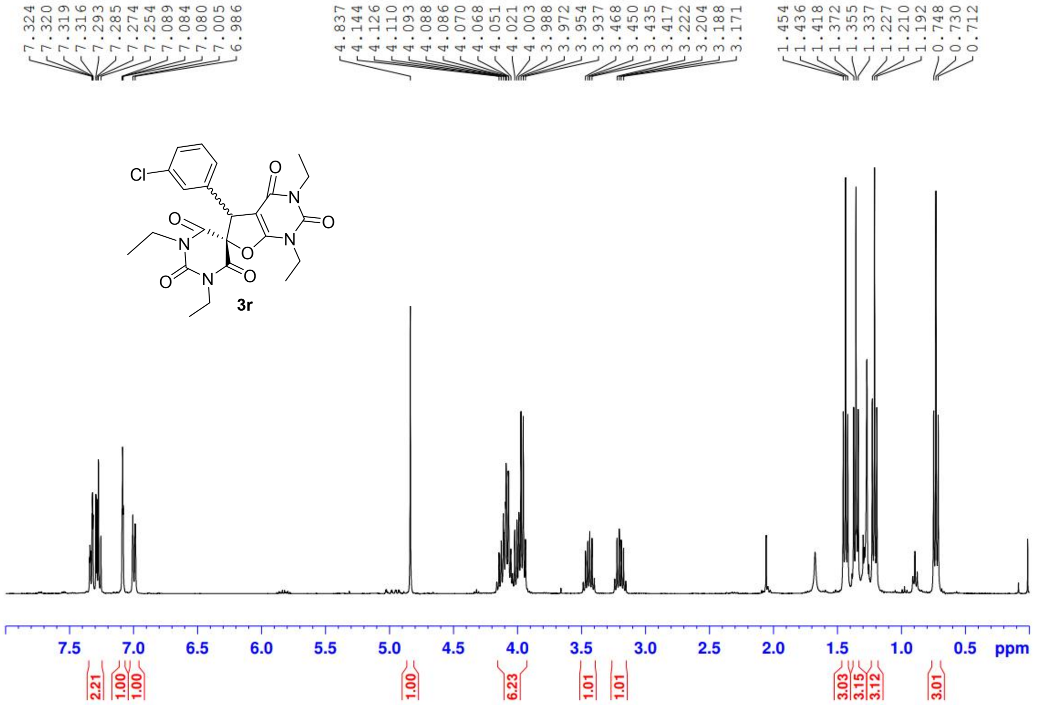

^{13}C NMR (100MHz,$CDCl_3$) of 3r

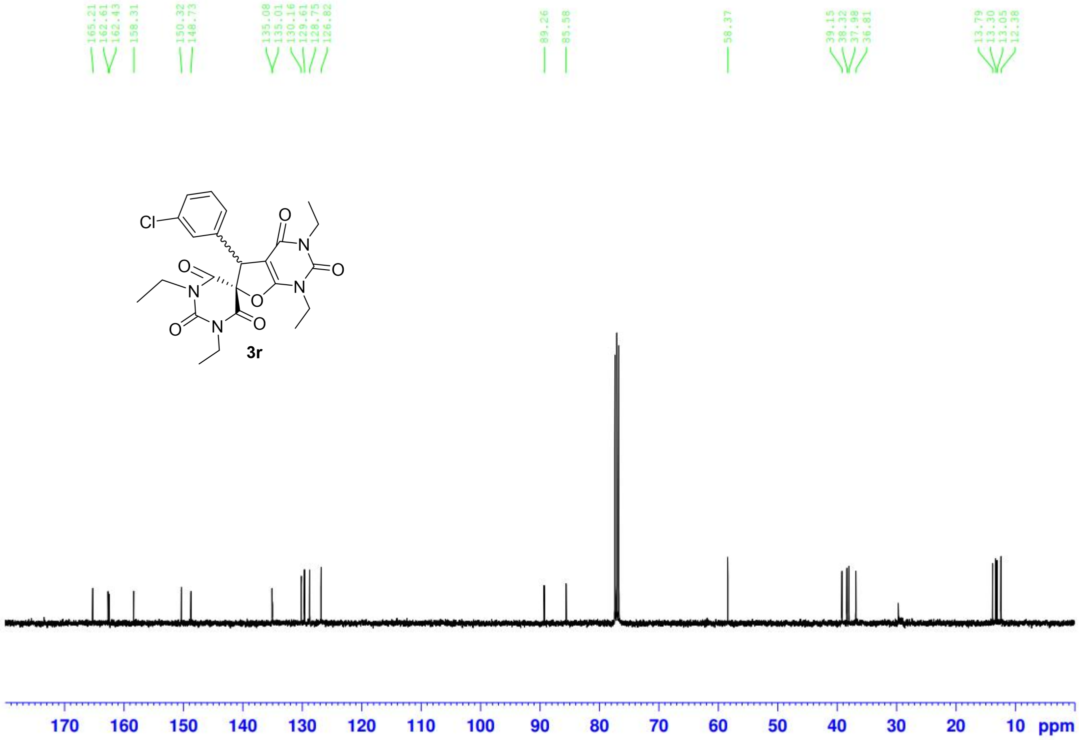

1,1',3,3'-tetraethyl-5-(4-(naphthalen-2-yl)phenyl)-1,5-dihydro-2H,2'H-spiro[furo[2,3-d]pyrimidine-6,5'-pyrimidine]-2,2',4,4',6'(1'H,3H,3'H)-pentaone (3s):

^{1}H NMR (400MHz,CDCl$_3$) of 3s

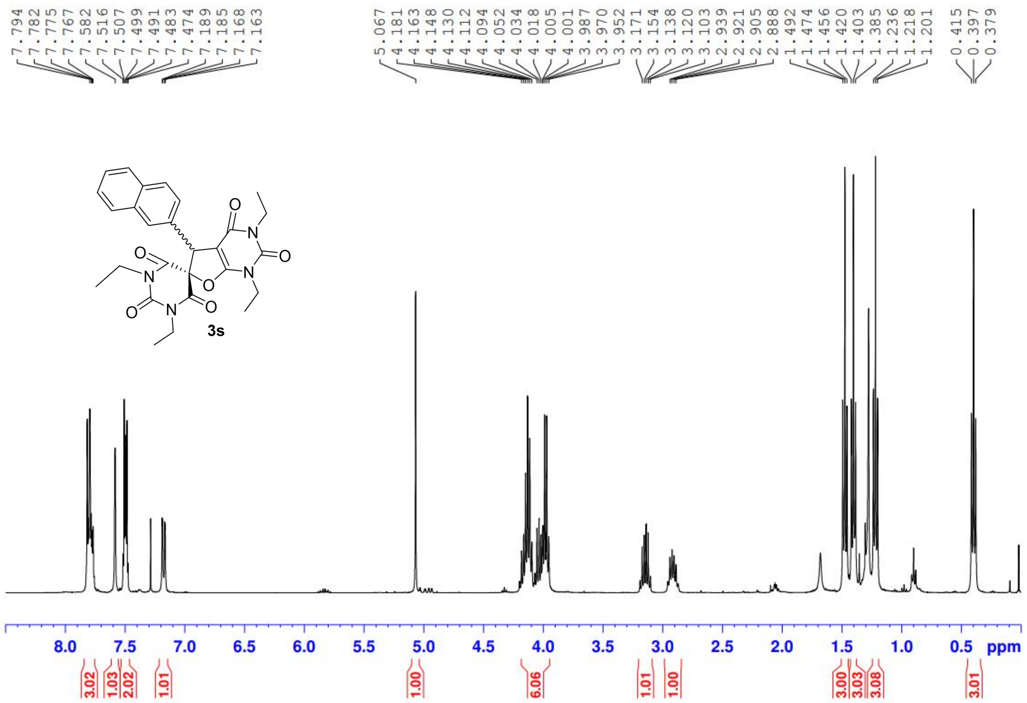

^{13}C NMR (100MHz,CDCl$_3$) of 3s

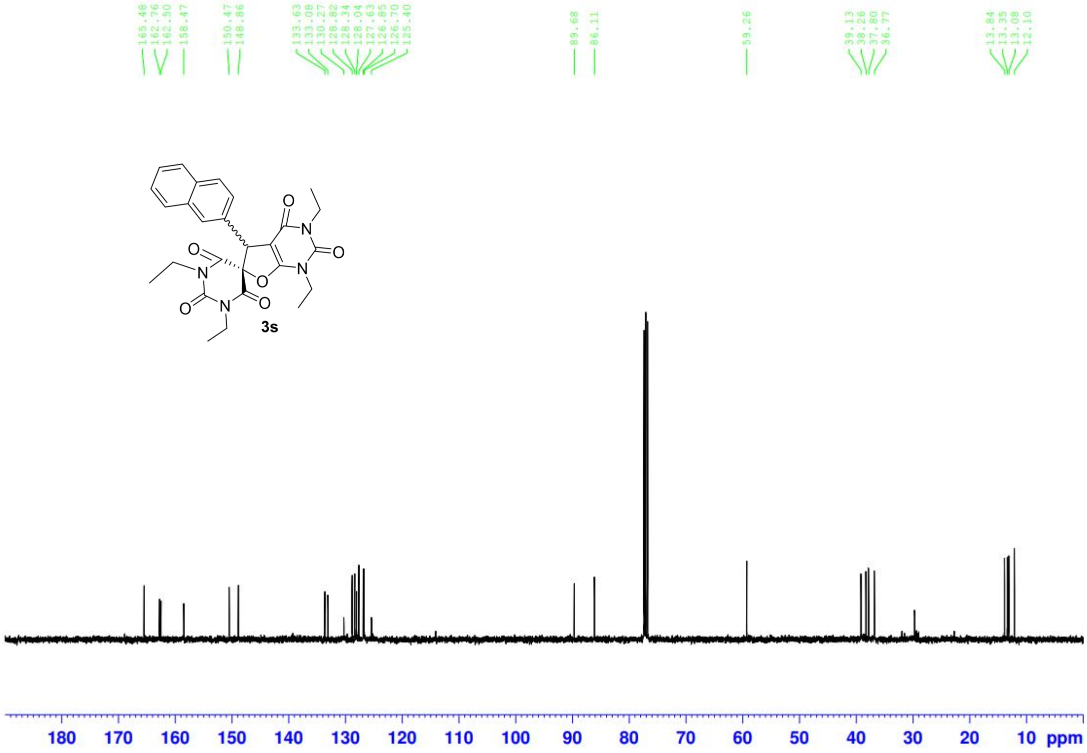

1,1',3,3'-tetraethyl-5-(4-methoxyphenyl)-1,5-dihydro-2H,2'H-spiro[furo[2,3-d]pyrimidine-6,5'-pyrimidine]-2,2',4,4',6'(1'H,3H,3'H)-pentaone(3t):

^{1}H NMR (400MHz,$(CD_3)_2CO$) of 3t

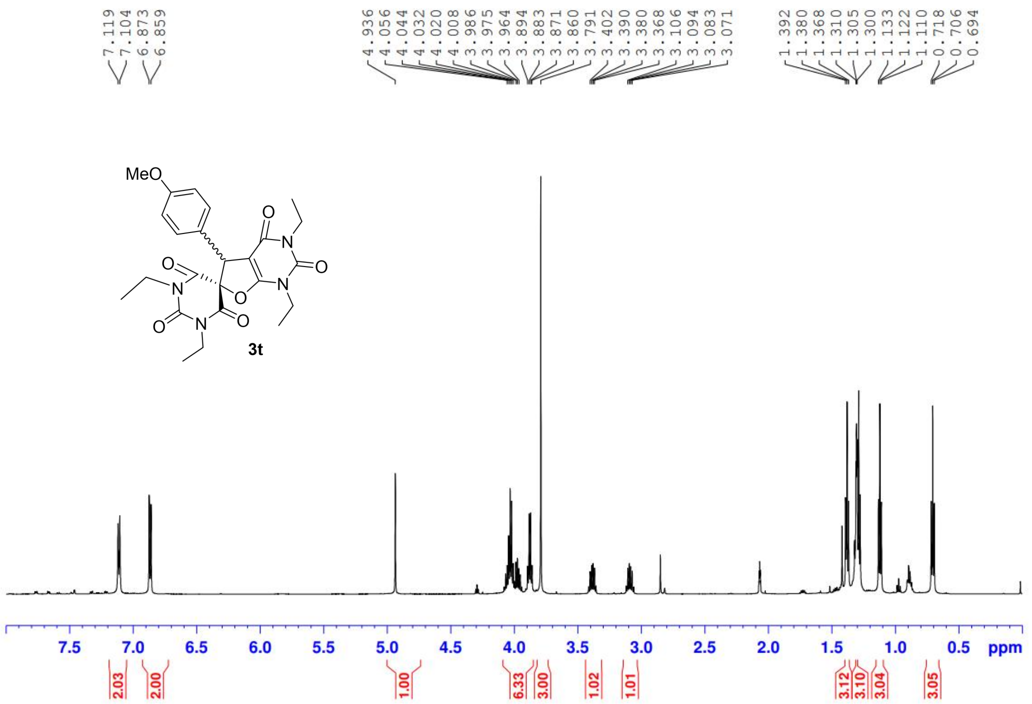

^{13}C NMR (100MHz,$CDCl_3$) of 3t

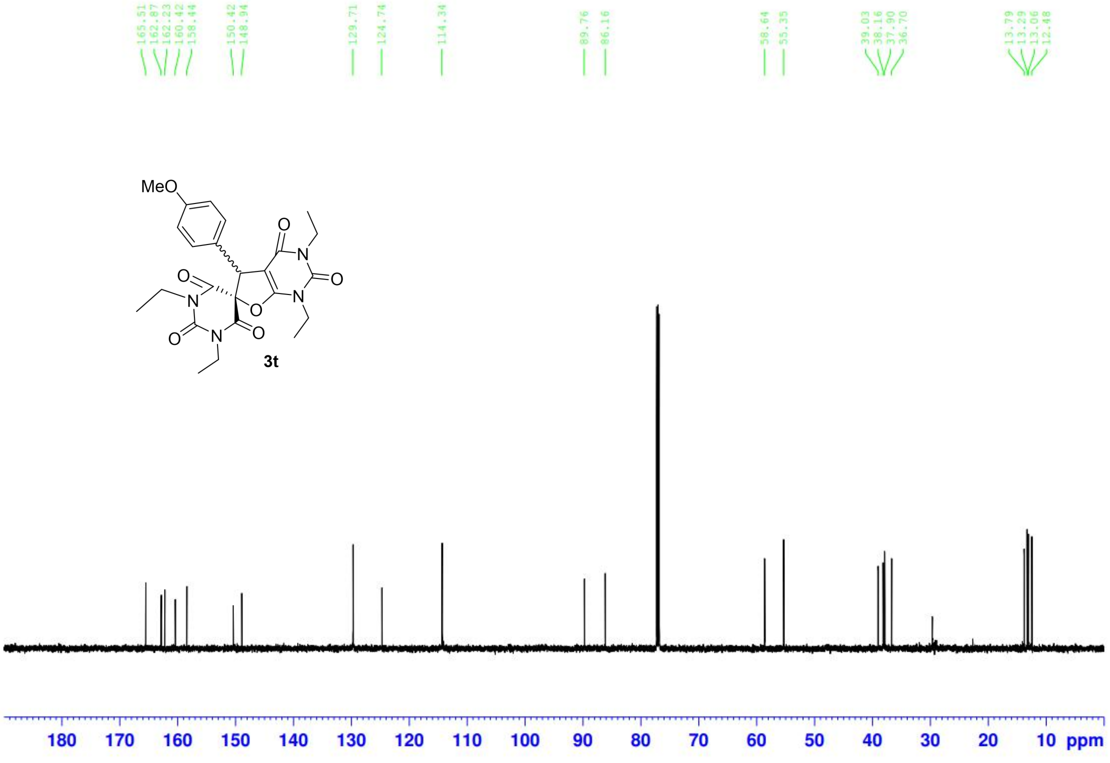

1,1',3,3'-tetraethyl-5-(4-nitrophenyl)-1,5-dihydro-2H,2'H-spiro[furo[2,3-d]pyrimidine-6,5'-pyrimidine]-2,2',4,4',6'(1'H,3H,3'H)-pentaone(3u):

^{1}H NMR (400MHz,CDCl$_3$) of 3u

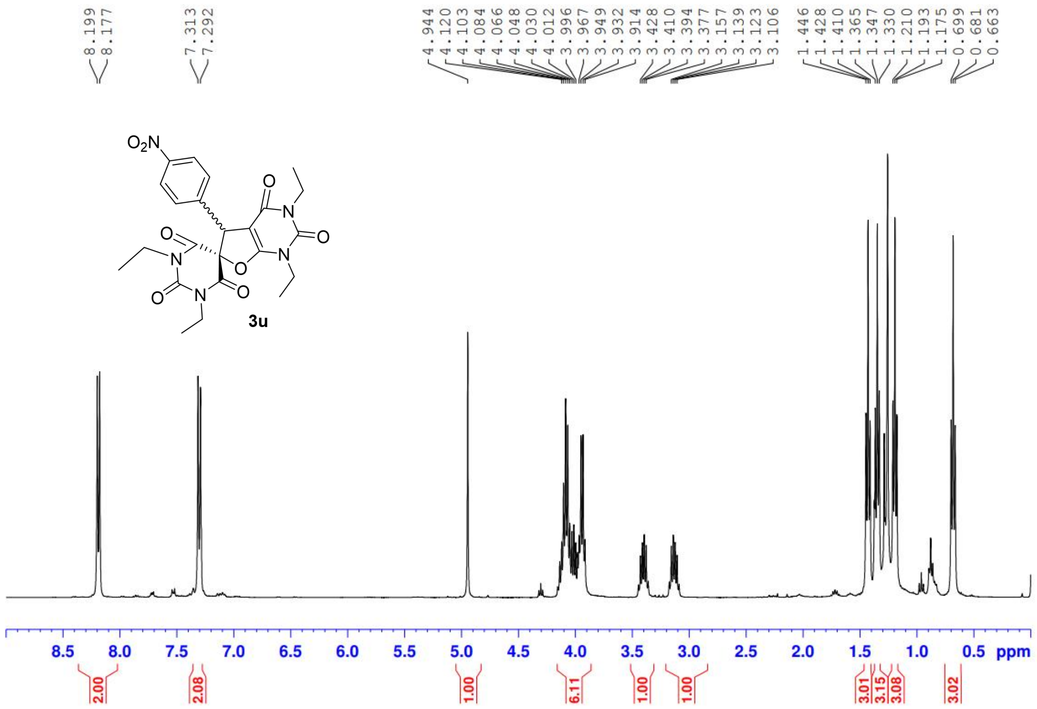

^{13}C NMR (100MHz,CDCl$_3$) of 3u

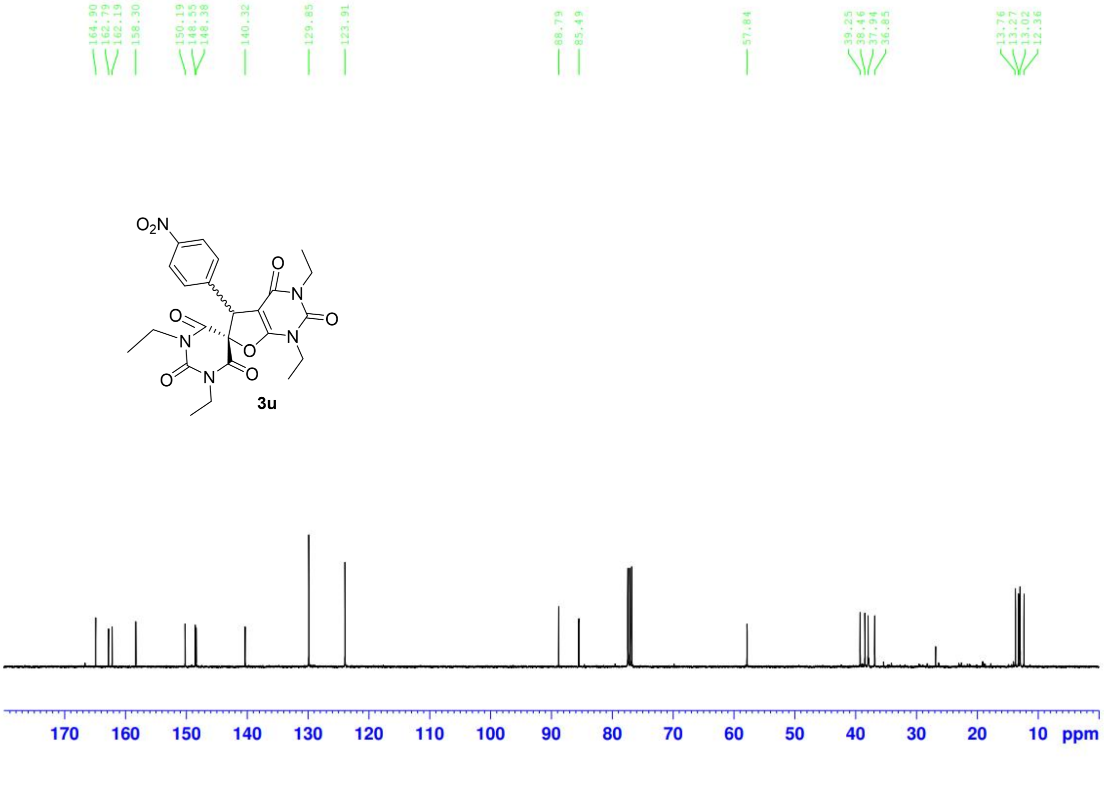

5-(2-bromophenyl)-1,1',3,3'-tetraethyl-1,5-dihydro-2H,2'H-spiro[furo[2,3-d]pyrimidine-6,5'-pyrimidine]-2,2',4,4',6'(1'H,3H,3'H)-pentaone (3v):

^{1}H NMR (400MHz,CDCl$_3$) of 3v

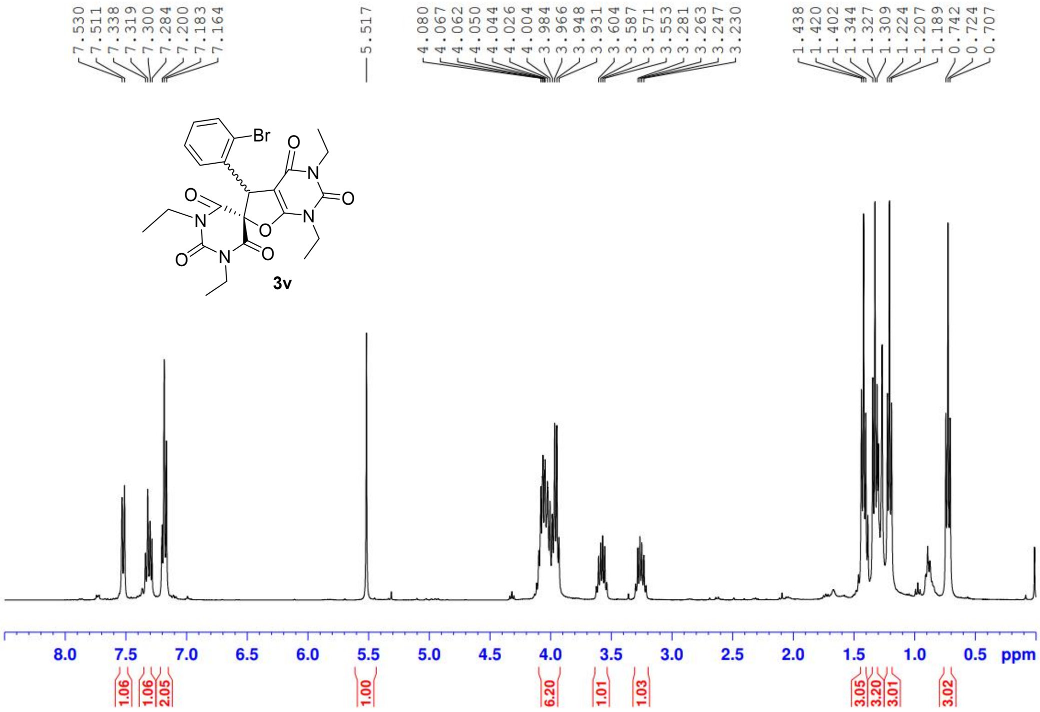

^{13}C NMR (100MHz,CDCl$_3$) of 3v

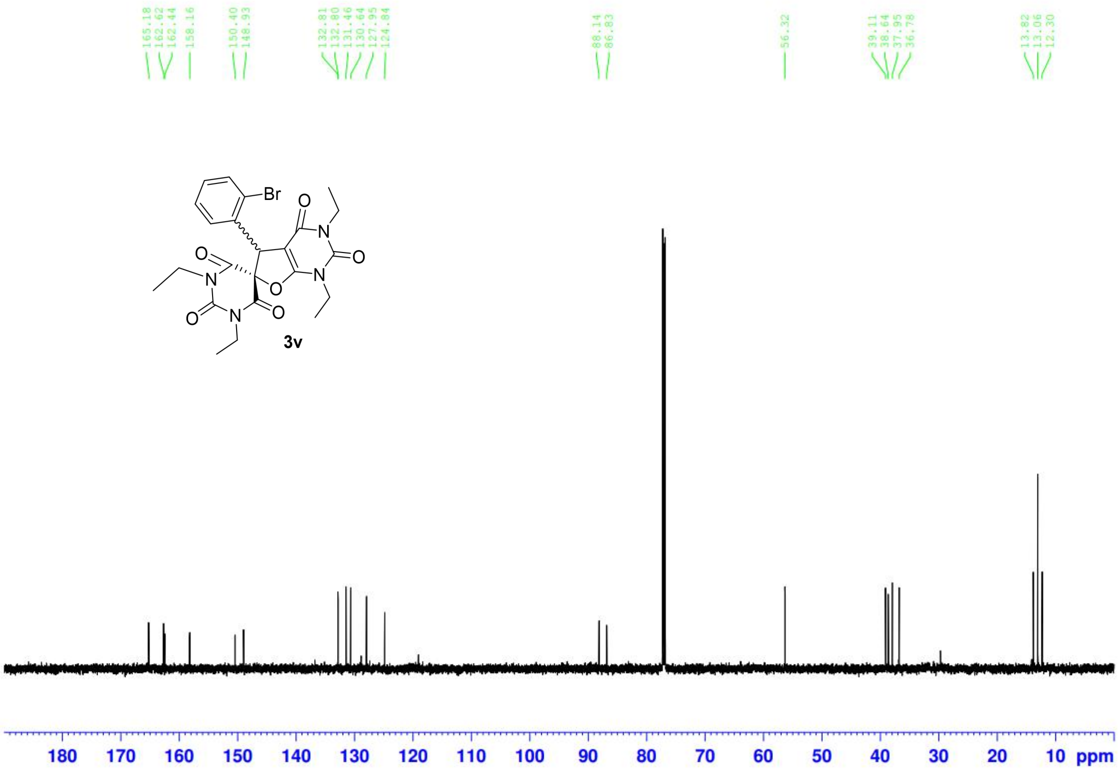

1,1',3,3'-tetraethyl-5-(m-tolyl)-1,5-dihydro-2H,2'H-spiro[furo[2,3-d]pyrimidine-6,5'-pyrimidine]-2,2',4,4',6'(1'H,3H,3'H)-pentaone (3w):

^{1}H NMR (400MHz,CDCl$_3$) of 3w

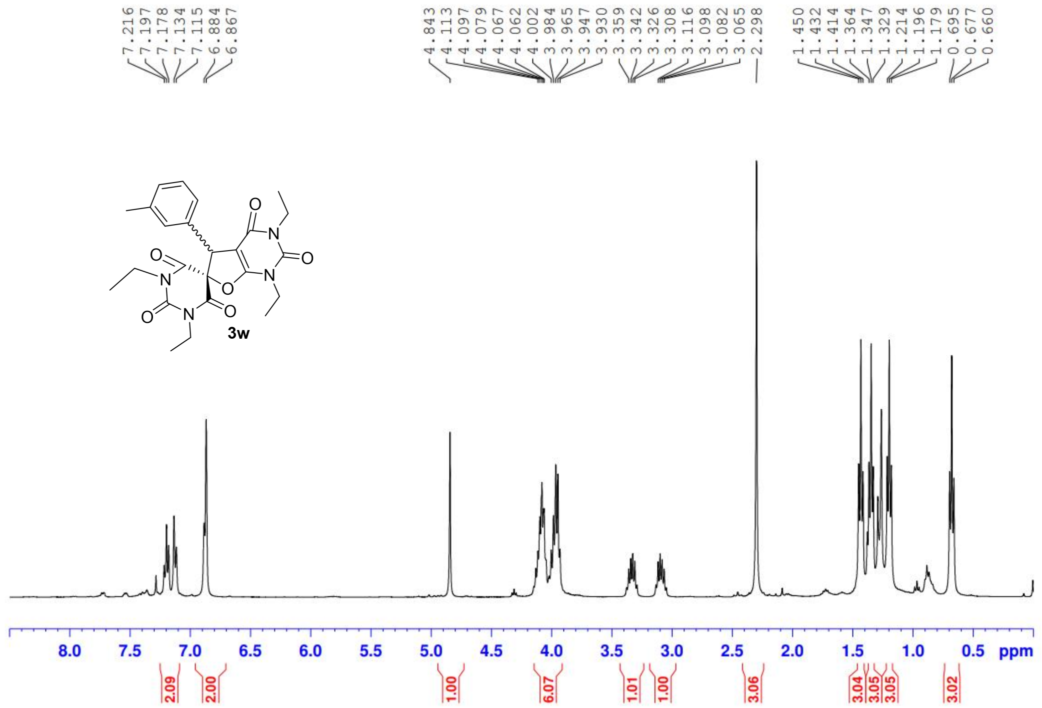

^{13}C NMR (100MHz,CDCl$_3$) of 3w

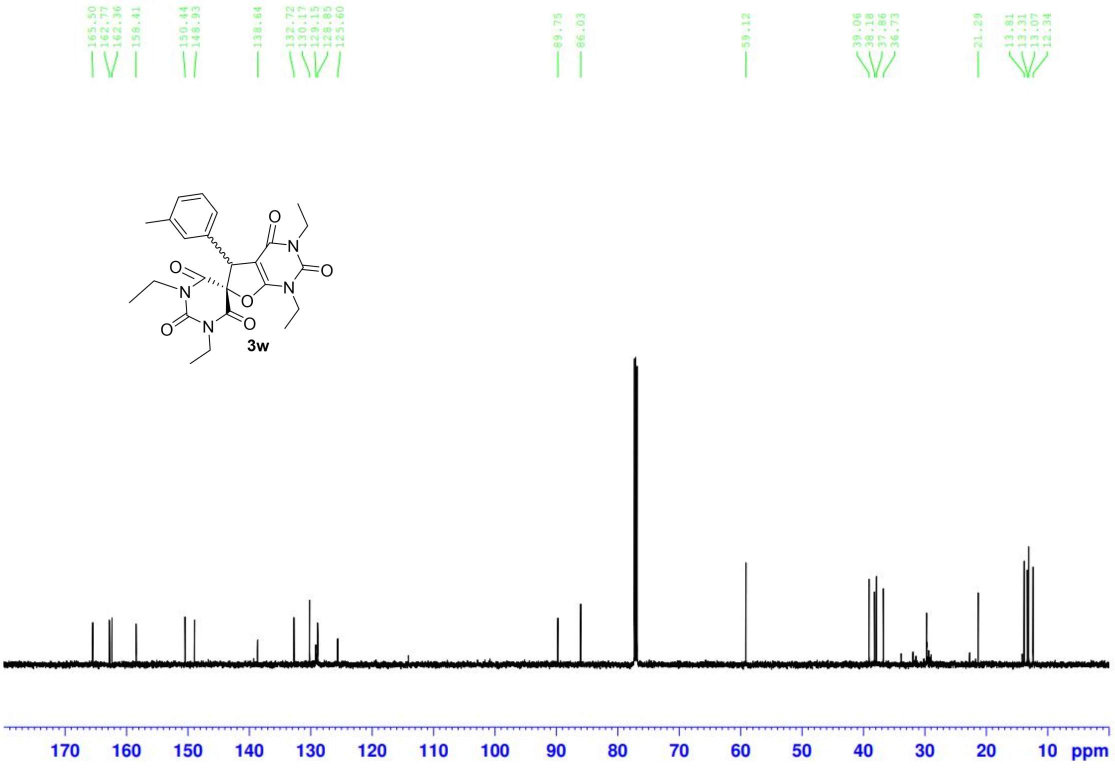

2. X-Ray Crystal Data of Product 3a

Single crystal of **3a** was obtained from the mixed solution of CH_2Cl_2 and petroleum ether via slow evaporation in a sample bottle. A suitable crystal was selected and its X-ray intensity data were measured at Rigaku-mm007 diffractometer with Mo Kα radiation. The crystal structure was solved by using the crysalispro and olex2 structure solution programs.

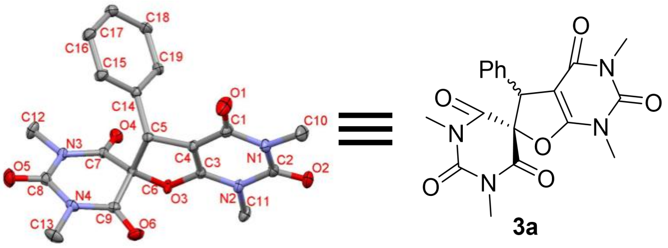

Figure S1. X-ray single crystal structure of **3a** (with thermal ellipsoils shown at the 50% probability level)

Table S1. Crystal data and structure refinement for 3a.

Identification code	**3a**
Empirical formula	$C_{19}H_{18}N_4O_6$
Formula weight	398.37
Temperature/K	113.15
Crystal system	triclinic
Space group	P1
a/Å	5.58300(10)
b/Å	8.7281(2)
c/Å	9.9792(2)
α /°	112.035(2)
β /°	93.0010 (10)
γ /°	98.9300(10)
Volume/Å^3	442.033(17)
Z	1
ρ calcg/cm^3	1.497
μ /mm^{-1}	0.114
F(000)	208.0
Crystal size/mm^3	0.29 × 0.25 × 0.2
Radiation	Mo Kα (λ = 0.71073)
2Θ range for data collection/°	5.128 to 69.374

Index ranges	-8 ≤ h ≤ 6, -13 ≤ k ≤ 13, -15 ≤ l ≤ 15
Reflections collected	11342
Independent reflections	5697 [R_{int} = 0.0257, R_{sigma} = 0.0335]
Data/restraints/parameters	5697/3/266
Goodness-of-fit on F^2	1.037
Final R indexes [I>=2σ (I)]	R1 = 0.0430, wR2 = 0.1105
Final R indexes [all data]	R1 = 0.0456, wR2 = 0.1134
Largest diff. peak/hole / e Å^{-3}	0.38/-0.24
Flack parameter	0.7(3)

3. Copies of HPLC Spectra of Products 3a

HPLC separation: Chiralcel OD-H, solvent: hexane/ethanol=80/20, flow rate=1.0 mL/min, λ=254 nm.

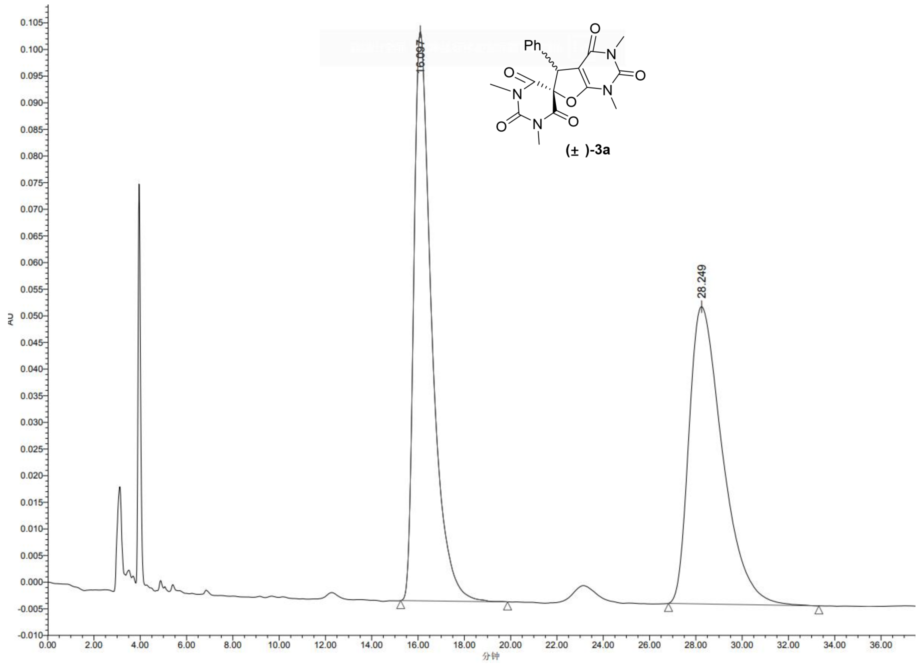

<Column Performance Report>

entry	R.T	Area	%Area	Height
1	16.097	5783726	50.84	106869
2	28.249	5593542	49.16	55795

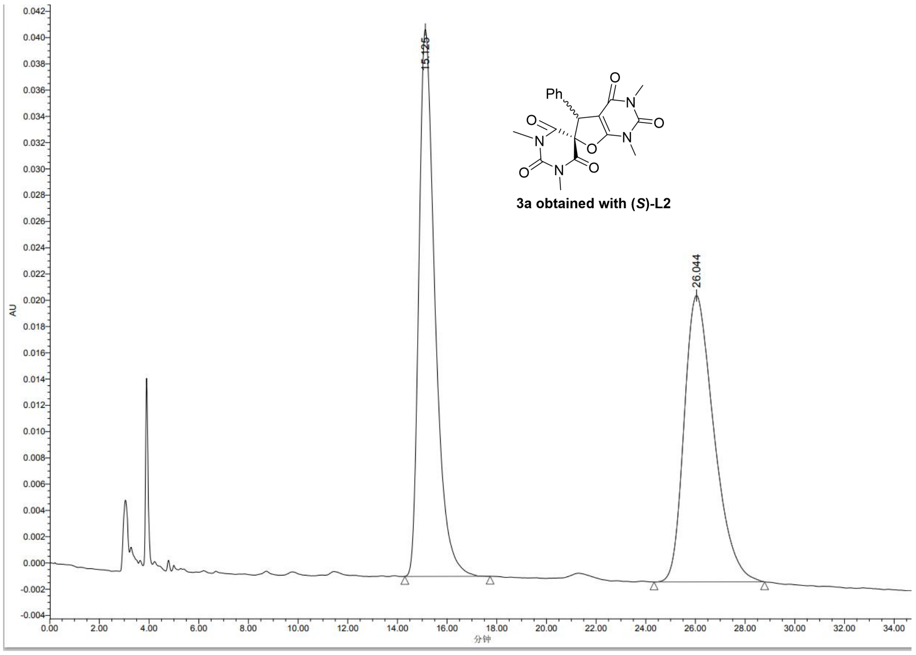

<Column Performance Report>

entry	R.T	Area	%Area	Height
1	15.125	1880625	50.85	41659
2	26.044	1817752	49.15	21810

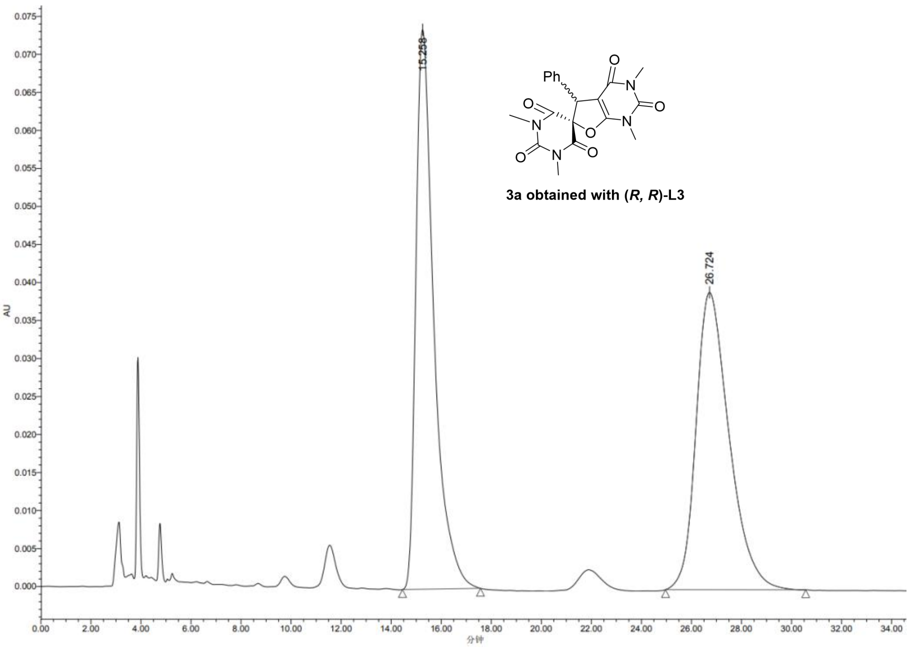

<Column Performance Report>

entry	R.T	Area	%Area	Height
1	15.258	3675491	50.55	73621
2	26.724	3595180	49.45	39107

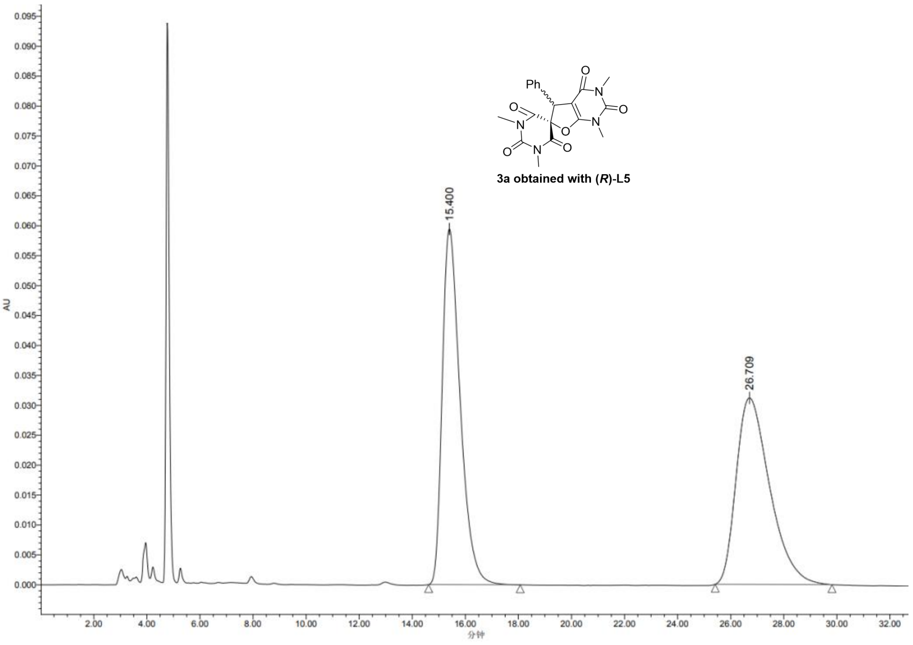

<Column Performance Report>

entry	R.T	Area	%Area	Height
1	15.400	2784942	50.43	59438
2	26.709	2737853	49.57	31167

4. Reaction Mechanism Investigation by LC-HRMS Analysis

In order to clarify the reaction mechanism, we took out a sample from the reaction mixture of substrates **1a** and **2b**, and performed its LC-HRMS analysis as shown below. The LC-HRMS analysis indicated the existence of the key species **2b**, **3a**, and **5** in the reaction mixture. On the basis of the discovery **5**, we assumed the reaction mechanism for the cascade assembly of **1a** with **2b**.

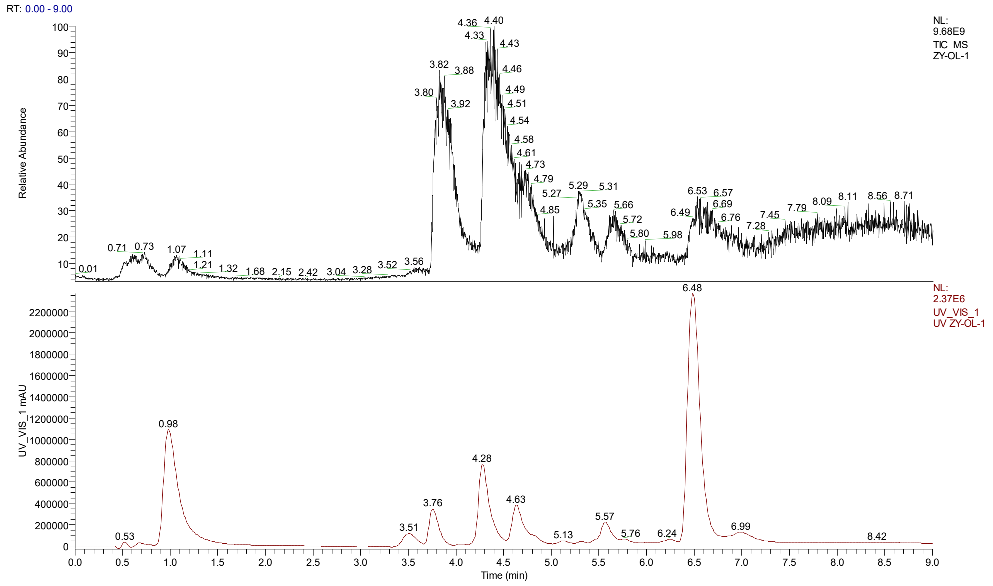

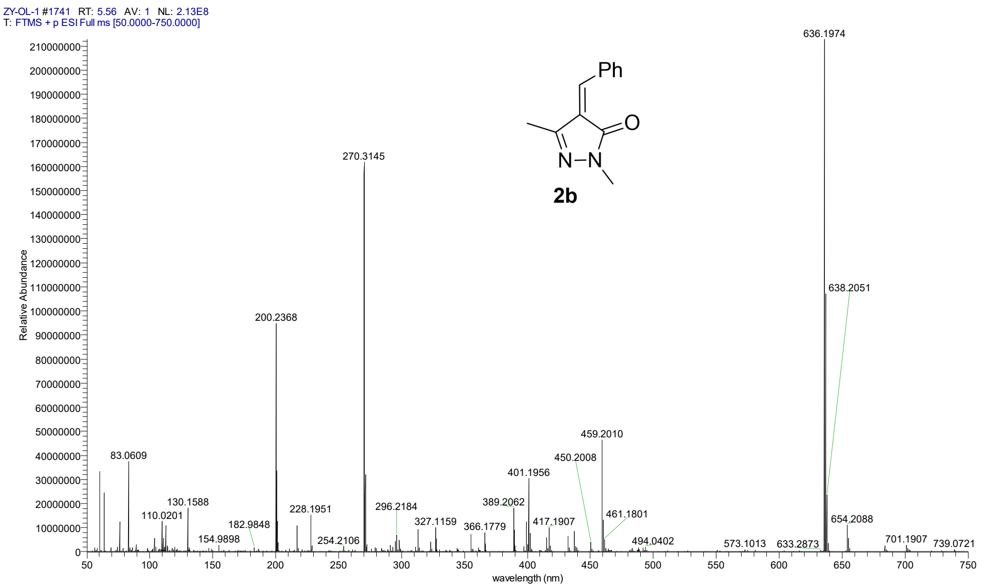

m/z	Theo. Mass	Delta (ppm)	RDB equiv.	Composition
201.10162	201.10224	-3.08	7.5	$C_{12}H_{13}ON_2$

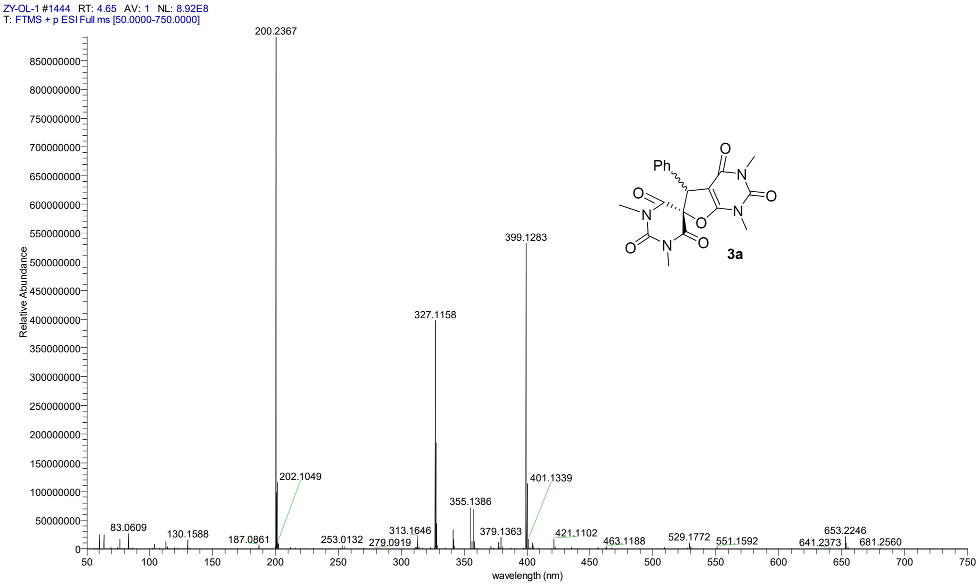

m/z	Theo. Mass	Delta (ppm)	RDB equiv.	Composition
399.12830	399.12991	-1.61	12.5	$C_{19}H_{19}O_6N_4$

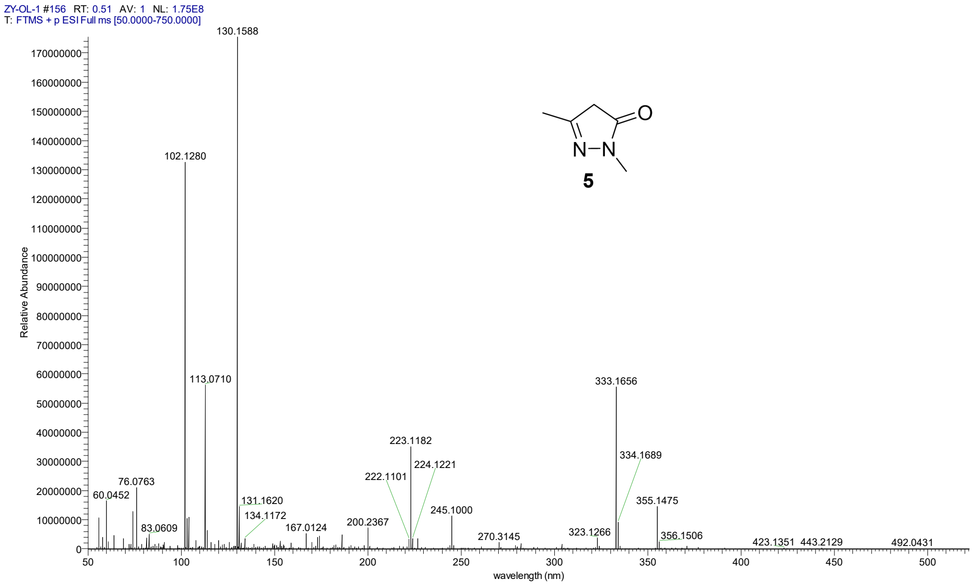

m/z	Theo. Mass	Delta (ppm)	RDB equiv.	Composition
113.07100	113.07094	0.54	2.5	$C_5H_9ON_2$
